# Expedient Discovery of a Metallaphotoredox Cyanomethylation for Synthesizing α‐Aryl Nitriles

**DOI:** 10.1002/chem.70946

**Published:** 2026-04-01

**Authors:** Gemma C. Cook, Blandine McKay, Craig Jamieson, Lee J. Edwards, Charlotte Harriman, Zackary S. Read, Simon T. Bate

**Affiliations:** ^1^ Molecular Modalities Capabilities (MMC) GSK Medicines Research Centre Stevenage Hertfordshire UK; ^2^ Department of Pure and Applied Chemistry WestCHEM University of Strathclyde Glasgow UK; ^3^ Drug Substance Development, Process Engineering & PAT GSK Medicines Research Centre Stevenage Hertfordshire UK; ^4^ CMC Statistics GSK Medicines Research Centre Stevenage Hertfordshire UK

**Keywords:** C‐C coupling, cross‐coupling, photocatalysis, photochemistry, synthetic methods

## Abstract

The α‐aryl nitrile motif is a valuable pharmacophoric feature in medicinal chemistry. However, current synthetic methods to prepare this moiety frequently employ harsh reaction conditions, highly toxic cyanide reagents, or have a limited substrate scope. To address this, a mild photocatalytic cyanomethylation reaction was developed, employing an iridium/nickel metallaphotoredox system in conjunction with supersilanol, cyclopropyl bromide and a mild base, and blue light. To identify a robust set of conditions to efficiently couple acetonitrile to a range of aryl bromides, extensive High‐Throughput Experimentation (HTE) was first applied. Subsequently, Design‐of‐Experiments (DoE) scoping and then screening experiments were performed to identify significant factors and interactions. Due to evidence of non‐linear effects, this was followed by an optimization experiment using a central composite design. Finally, to confirm the conditions identified, a robustness DoE study was performed. The methodology demonstrated tolerance to a variety of functional groups, and was applicable to a range of medicinally‐relevant building blocks through library synthesis. The reaction was also applied to the multigram preparation of a key anti‐cancer Senexin intermediate, which shortened the synthetic route and obviated the need for a cyanide reagent.

## Graphical abstract

A metallaphotoredox cyanomethylation reaction has been developed for the synthesis of valuable α‐aryl nitrile intermediates. The methodology was successfully applied to diverse medicinally‐relevant substrates, providing an expedient route to anti‐cancer Senexin compounds, while also removing the need for toxic cyanide reagents. The mechanism was probed through deuteration and fluorescence quenching experiments.

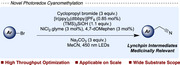

## 1 Introduction

**FIGURE 1:**
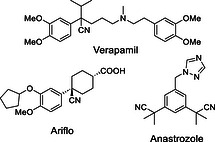
Marketed drugs featuring the α‐aryl nitrile motif.

Nitriles are valuable functional groups in the field of medicinal chemistry; they can form interactions with proteins of interest through hydrogen bonding, dipole‐dipole or hydrophobic interactions, or through covalent bonds [1, 2, 3]. The relatively small size of the nitrile group can facilitate interactions within tight binding pockets, furnishing unique potency and selectivity [1]. A number of marketed drugs, including Verapamil, Ariflo, and Anastrozole, contain the α‐aryl nitrile motif (Figure 1).

**SCHEME 1:**
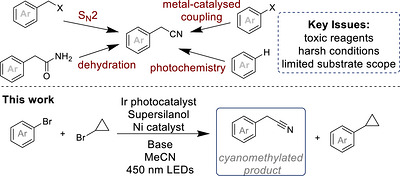
Methods for the formation of α‐aryl nitriles.

In addition to their presence in medicines, α‐aryl nitriles are useful synthetic intermediates. From this versatile handle, a wide range of other functional groups can be accessed, such as imines, ketones, esters, and tetrazoles, as well as the privileged pharmacophores, β‐aryl amines and carboxylic acids [4, 5]. Many of the existing methods for the formation of α‐aryl nitriles require the use of highly toxic cyanide reagents, which are unsuitable for use in an industrial setting [6, 7, 8, 9, 10]. Other reactions require harsh conditions such as strong bases or high temperatures [11, 12, 13, 14, 15, 16, 17, 18], which limit the functional group tolerance. In other cases, pre‐functionalized starting materials are required, such as cyanohydrins [19], cyanoacetates [21, 22, 23], silyl and zinc nitrile reagents [24], or isoxazole‐4‐boronic acid pinacol ester [25]. These limitations prevent the aforementioned methodologies from being applied to library synthesis for rapid and diverse Structure Activity Relationship (SAR) expansion. Although there are reports of visible‐light‐mediated methods to generate the cyanomethyl radical from either a haloacetonitrile [26, 27, 28, 29] or acetonitrile itself [30, 31, 32], the incipient radical has only been demonstrated to react with a very limited range of substrates, and due to these limitations, could not be applied to the synthesis of α‐aryl nitriles. Related to this, and to the best of our knowledge, the cyanomethyl radical has not been shown to undergo cross‐coupling with aryl halides under a metallaphotoredox manifold (Scheme 1).

## 2 Results and Discussion

**SCHEME 2:**
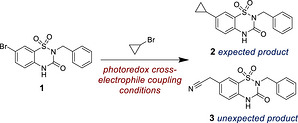
Photoredox cross‐electrophile coupling of an aryl bromide (**1**) with alkyl bromide, to form the expected cross‐coupled product (**2**), as well as the unexpected cyanomethylated product (**3**).

Our initial work focused on the application of the metallaphotoredox cross‐electrophile coupling (XEC) conditions reported by MacMillan [33] to couple cyclopropyl bromide to an aryl bromide, during which we observed coupling of the acetonitrile solvent to the aryl bromide scaffold (Scheme 2).

**FIGURE 2:**
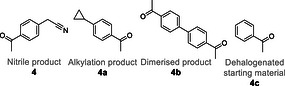
Four key species monitored throughout the optimization.

Given the interest in the application of the α‐aryl nitrile unit in both synthetic and medicinal chemistry described above, we sought to further develop this nascent transformation. Using the XEC reaction conditions shown above as a starting point, and bromoacetophenone as a model substrate, this unanticipated cyanomethylation reaction was optimized using high‐throughput experimentation (HTE). HTE is an integral step towards reaction optimization in an industrial setting, allowing the chemist to effectively route scout and find suitable reaction conditions, while minimizing the quantities of materials needed, and maximizing time efficiency [34, 35]. In our standardized HTE process, the relative success of each reaction was determined from the LCMS peak area of the desired product relative to that of an internal standard (IS), *N*,*N*‐dibenzylaniline. The extent of dehalogenation (**4c**), XEC product formation (**4a**), dimerized byproduct (**4b**), and the amount of starting material remaining were also monitored throughout the optimization trajectory (Figure 2).

Initial control reactions demonstrated the requirement for light, photocatalyst, nickel catalyst, and supersilanol reagent (Table 1, entries 2–5). In the absence of either base (entry 6) or cyclopropyl bromide (entry 7), formation of the nitrile product was reduced, indicating that these components have a beneficial, but not essential, role in the reaction mechanism.

**TABLE 1:** Ratio of nitrile product (**4**) formed to the internal standard (IS) for each control reaction.

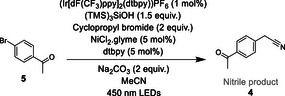		
Entry	Conditions	4/IS
1	Control	2.8
2	Without light	0.0
3	Without iridium	0.0
4	Without nickel	0.0
5	Without silanol	0.0
6	Without base	0.3
7	Without cyclopropyl bromide	0.8

When nickel was excluded from the reaction, significant dehalogenation occurred. This is reasoned to be a light‐driven process, whereby the iridium photocatalyst may act as a photosensitizer (see Table S2). Upon investigation into the role of the cyclopropyl bromide, a variety of alternative alkyl halides were examined (see Table S4), but did not offer an increase in product formation. The unique increase in conversion gained with cyclopropyl bromide is attributed to the ability of cyclopropyl radicals to abstract a hydrogen atom from acetonitrile [36], generating a radical species (*vide infra*). Next, a selection of photocatalysts with a wide range of redox potentials and triplet energies was tested, alongside both inorganic and organic bases. This process led to the identification of [Ir(ppy)_2_(dtbpy)]PF_6_ and sodium acetate as an alternative photocatalyst and base, respectively (see Table S5).

Seven nickel catalysts and six ligands were assessed (Table 2). It was found that by replacing the 4,4’‐di‐*tert*‐butyl‐2,2’‐dipyridyl (**L1**) ligand with a more electron‐rich dimethoxy‐phenanthroline ligand (**L4**), a substantial improvement in desired product formation was realized. This is attributable to increased selectivity for cyanomethylation over the cyclopropyl alkylation. Furthermore, nickel(II) bromide trihydrate was also shown to be a competent catalyst for this reaction, which has the potential to offer cost savings when performing this reaction on an industrial scale.

**TABLE 2:** Ratio of product (**4**) to internal standard when various nickel sources and ligands are employed to the coupling of 4‐bromoacetophenone (**5**) with acetonitrile.

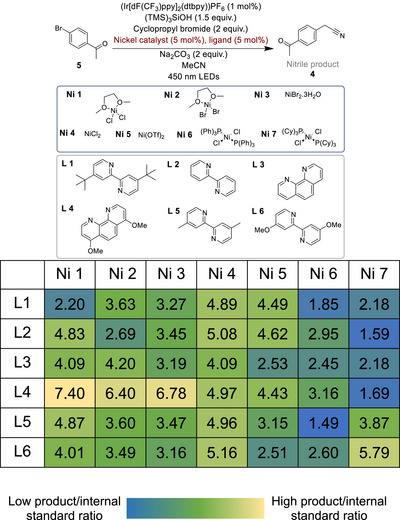

**SCHEME 3:**
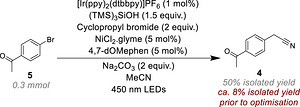
Preferred conditions following high‐throughput optimization of photocatalyst, alkylating agent, nickel catalyst, base, and wavelength.

In order to ensure the chosen reaction conditions could be used across a wide range of substrates in a parallel format, Library Validation Experiments (LVEs) were performed. These experiments tested different combinations of the preferred conditions from each screen across a diverse set of substrates (see Tables S7–S9), which led to the broadly applicable conditions shown in Scheme 3. Use of these conditions with the model substrate resulted in an isolated yield of 50%, a significant increase compared to the 8% yield achieved prior to optimization efforts.

**FIGURE 3:**
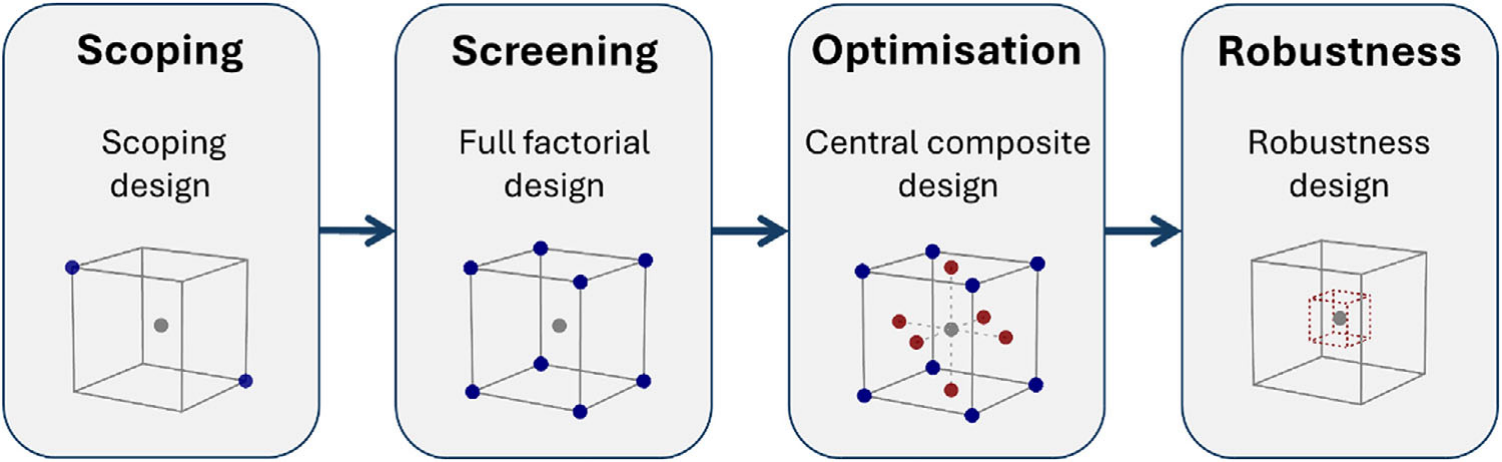
Design of Experiments (DoE) campaign.

Having optimized the discrete variables, a Design of Experiments (DoE) approach was applied to determine the optimal stoichiometry of the catalysts and reagents [37, 38, 39, 40]. The four‐stage DoE campaign started with a scoping design to assess factor ranges, followed by a screening design to identify which factors and their interactions influence the process (Figure 3). Along with the factorial points (at the low and high levels of the individual factors), center points were also included to assess whether the relationships were linear or if there was evidence of nonlinear trends. Due to evidence of nonlinearity (curvature), the design was augmented to generate a central composite design by the inclusion of axial points. This allowed non‐linearity to be explored, thus identifying the factors causing curvature. Once a model was selected and a target set of conditions that maximized product formation was identified, a robustness study was performed to confirm how robust the process was to small changes in the factors.

**FIGURE 4:**
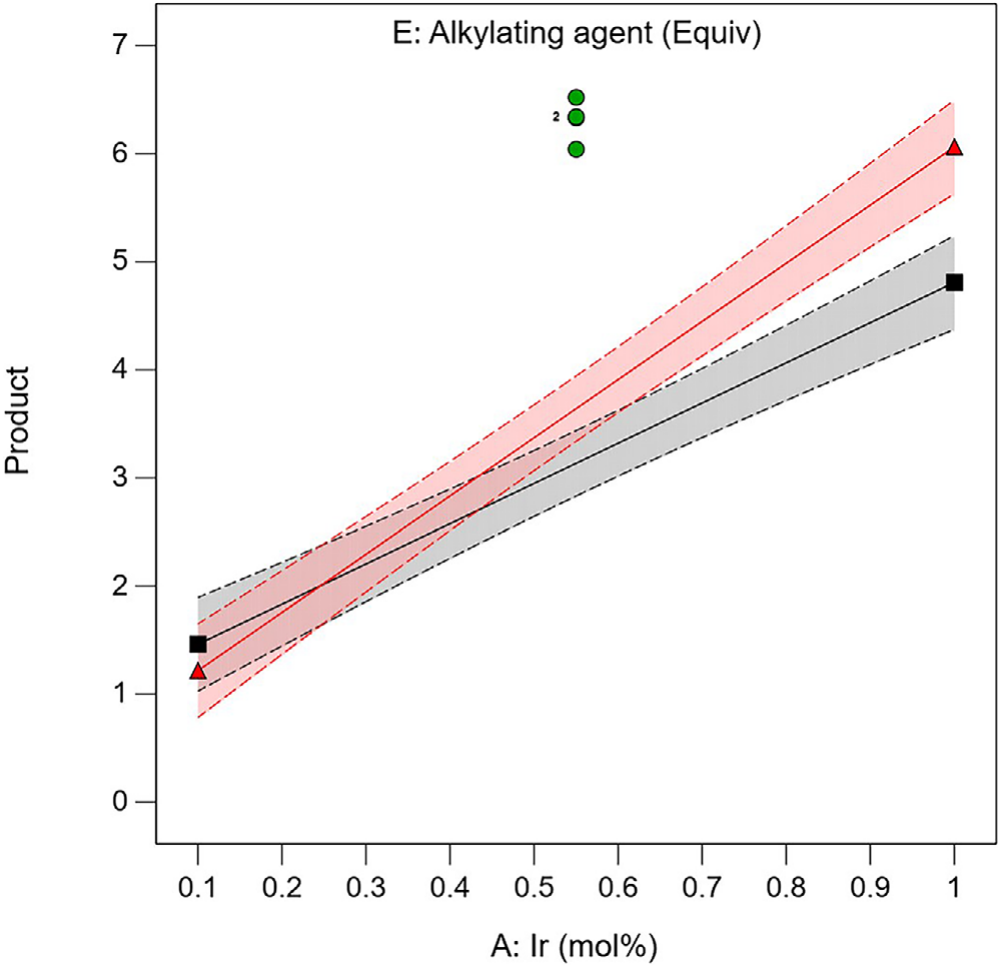
The effect of increasing iridium concentration on the P/IS ratio, at low (black line) and high (red line) equivalents of alkylating agent. Center points are shown as green circles.

Following the scoping DoE, a full‐factorial design with 4 center points was performed, which examined five factors (the stoichiometry of iridium, nickel, supersilanol, base, and alkylating agent). This screening, DoE revealed a strong positive correlation between iridium stoichiometry and product formation (Figure 4).

Significant curvature was observed in these results, as inferred from the position of the center points in Figure 4; therefore, the design was augmented with 10 axial points, as well as additional factorial and center point runs. From the augmented face‐centered central composite design, a backwards selection approach was employed to select the final model, where all main effects, two and three‐factor interactions, and quadratics were considered for inclusion (see Supporting Information for more details). From this analysis, the curvature was identified to be caused by non‐linear effects of iridium photocatalyst and nickel catalyst stoichiometry (see Table S18).

**FIGURE 5:**
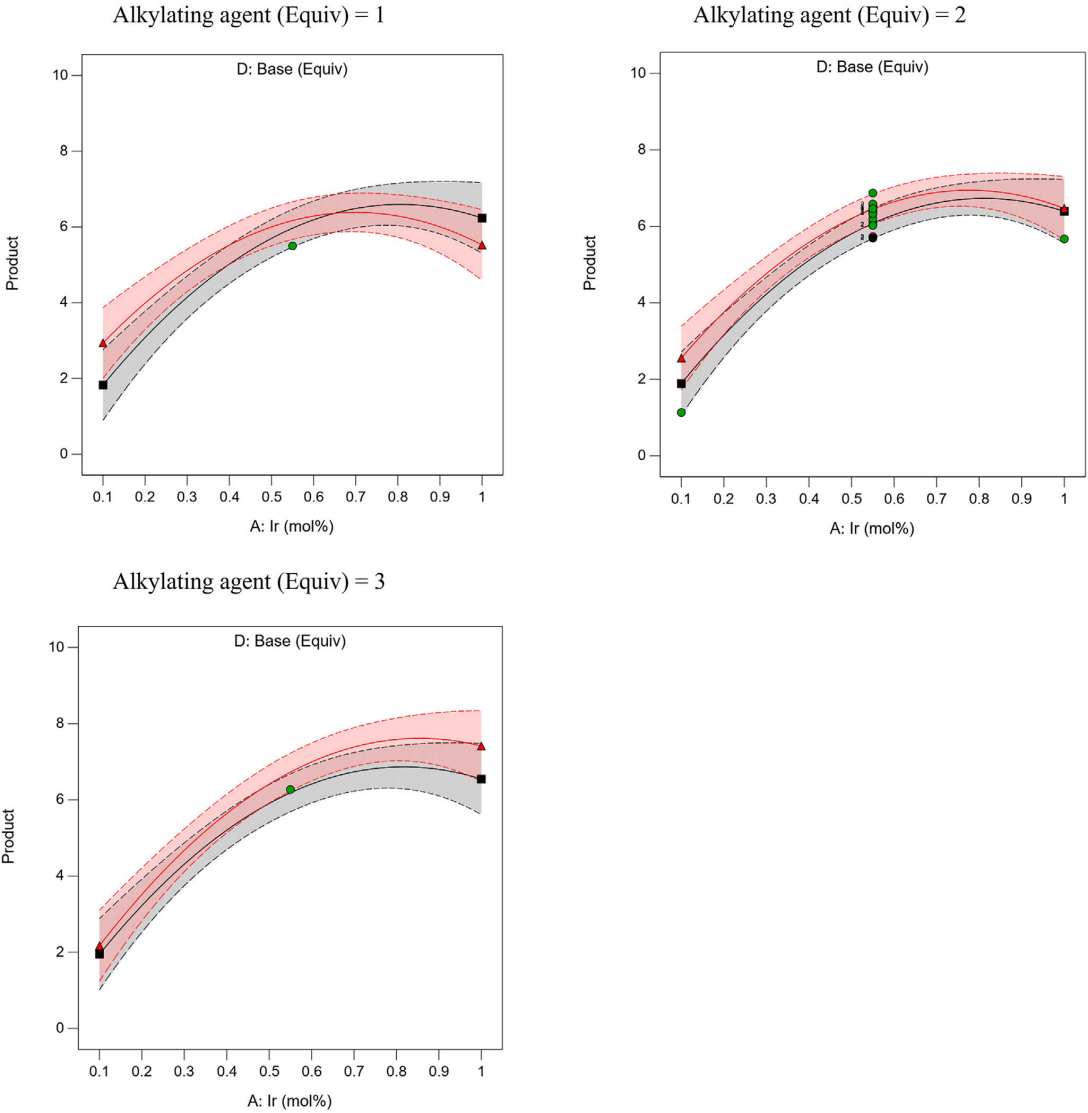
The effect of iridium loading on the product/internal standard ratio at low, center point, and high levels of alkylating agent equivalents, at low (black line) and high (red line) equivalents of base. Center points (green circles) are also included.

Increasing the quantity of iridium photocatalyst was found to increase product formation, up to approximately 0.85 mol% catalyst loading. Increasing the stoichiometry of the iridium photocatalyst was also found to increase the undesired dehalogenation and alkylation pathways, and, consequently, drives consumption of starting material (see Supporting Information for all DoE data). It can be seen from Figure 5 that with only one equivalent of alkylating agent (cyclopropyl bromide), the increase in product formation upon increasing iridium loading is lowered when a higher amount of base is added. However, when three equivalents of alkylating agent are used in the reaction, the increase in product formation upon increasing iridium loading is amplified by higher quantities of base. We hypothesize that high quantities of both iridium photocatalyst and alkylating agent would result in increased formation of HBr; an increase in stoichiometry of base can therefore assist in buffering the reaction mixture.

**FIGURE 6:**
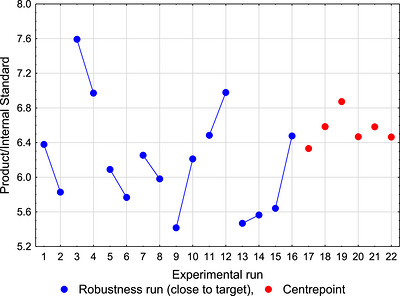
Variation in product/internal standard across robustness reactions (blue), relative to center point reactions (red). Dots linked together depict reactions with identical reaction conditions, where variation is due to well‐to‐well variability, or plate‐to‐plate variability, rather than deliberate variation in the amount of reagent dosed.

Finally, the robustness of the reaction was assessed by testing the effect of small variations of the factors from the center point conditions (Figure 6).

In Figure 6, a greater variation in the P/IS ratio can be noted across the robustness reactions (blue), compared with the center point reactions (red), which indicates that the effect of varying the factors is greater than the standard reaction‐to‐reaction variation. Reactions with high levels of iridium present (e.g., 3 and 4) had P/IS values significantly greater than the center point reactions, while wells with lower iridium loadings (e.g., 1 and 2) had P/IS values below the center point reactions. From consideration of the above, iridium stoichiometry must be tightly controlled in order to achieve high reaction robustness, as this factor is the largest contributor to variability in the process. In some cases, there was a prominent difference in the P/IS values resulting from identical experiments (which are joined together with a blue line on the figure); this was due to either well‐to‐well variation or plate‐to‐plate variation.

Although a nickel‐supersilanol interaction was found to be significant when considering the *p*‐value, changing the stoichiometry of the supersilanol had little practical effect on the product formation (see Figure S5). Therefore, although the optimum conditions suggested by the model would recommend high levels of supersilanol, it was reasoned that using such an excess of a high‐cost reagent may not be justified. To confirm this, a range of supersilanol stoichiometries were screened. These results showed that the equivalents of supersilanol could be reduced to 1, which makes the reaction more economical and improves atom economy. Within this high‐throughput experiment, different concentrations were also tested. No significant impact on reaction outcome was seen; the highest concentration tested (0.1 M) was used going forward, to allow higher quantities of material to be synthesized within the library synthesis format.

Taken as a whole, the DoE campaign resulted in a reduction in the stoichiometry of both metal catalysts with no appreciable reduction in yield, thus improving the suitability of this reaction for industrial applications. Furthermore, an elevated understanding of which factors affect the levels of dehalogenated and alkylated side products allows for greater control over the formation of these species, leading to a carefully designed optimum, which maximizes product and minimizes the formation of side products. Further insights can be found in Section S3.3.

**FIGURE 7:**
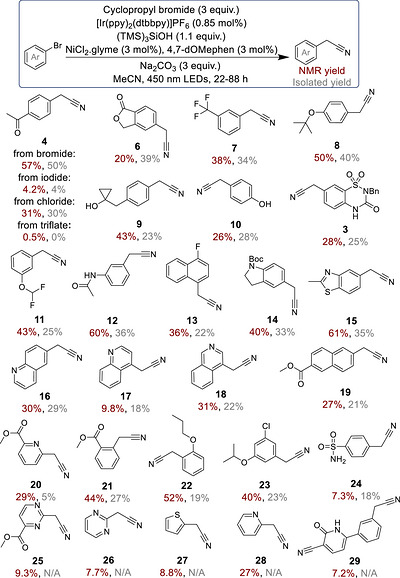
Photoredox cyanomethylation substrate scope.

The fully optimized conditions were subsequently applied to a range of substrates, which were selected based on their relevance to medicinal chemistry applications (Figure 7). In addition to isolated yields, NMR yields were measured using maleic acid as an internal standard.

Pleasingly, a wide range of functional groups were tolerated, including amides (**12**), alcohols (**9**), phenols (**10**), ketones (**4**), sulfonamides (**24**), and ethers (**8**, **11**, **22**, and **23**). Steric hindrance around the aryl bromide did not appear to be a limitation, as high conversion was also achieved for *ortho*‐substituted systems (**21** and **22**). It was found that **4** could not be prepared from the aryl triflate, and using the iodide led to significant dehalogenation, presumably due to the weaker carbon‐halogen bond strength and lower reduction potential [41, 42]. However, the chloride proved to be a suitable coupling partner, with minimal dehalogenation occurring, albeit requiring a longer reaction time. Due to the difference in reaction rates between the halides, it was shown that the bromide could be selectively functionalized in the presence of a chloride (**23**), providing a useful chemical handle for subsequent derivatization.

Numerous pharmacologically relevant heterocycles proved to be competent substrates, including quinolines (**16**, **17**, and **18**) and a benzothiazole (**15**), although substantial homocoupling of 2‐bromopyridines was observed (**20**). Unprotected and tertiary amines were shown to be incompatible with this reaction, likely due to reductive quenching of the photocatalyst (for unsuccessful substrates, see Section S4.4) [43]. In some cases, the NMR yield was noted to be lower than the isolated yield. As the NMR analysis was conducted on the crude reaction mixtures, it is reasoned that this was likely due to an interfering signal from an impurity or another reaction component. Differences in relaxation times between different molecules can also affect the measured integrals.

Initial investigations into the mechanism of the transformation began by identifying a reagent that may abstract a hydrogen atom from acetonitrile. To this end, reactions were performed in MeCN‐d_3_ in order to detect deuterated species in the reaction milieu. It was anticipated that the cyclopropyl radical may form following halogen abstraction by a silyl radical; phenyl‐substituted cyclopropyl bromide was used in place of cyclopropyl bromide to reduce volatility and facilitate detection. These investigations were carried out concurrently with optimization of reaction conditions, hence they do not reflect the final conditions that were developed.

**SCHEME 4:**
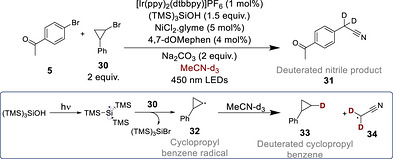
Model reaction performed in MeCN‐d_3_, in order to detect a deuterated hydrogen atom abstraction reagent.

The reaction depicted in Scheme 4 formed the deuterated nitrile product (**31**) in 11% conversion; deuteration was confirmed by GCMS and NMR. Deuterated cyclopropyl compound **33** was detected by GCMS, which suggests that a cyclopropyl radical is formed, which acts as a hydrogen atom abstraction reagent. It is proposed that the cyclopropyl radical is generated from the corresponding bromide *via* silyl radical‐mediated halogen abstraction [33]. Although supersilanol was used in these reactions, supersilyl bromide was generated as a by‐product; this may suggest formation of the supersilyl radical, rather than the expected oxygen‐linked species (see Scheme S1). It is noted that the reaction does occur, albeit to a lesser extent, when no cyclopropyl bromide reagent is present, and hence it is likely that another species is capable of abstracting the hydrogen atom in this case, which we propose could be the silyl radical itself.

Stern‐Volmer quenching experiments revealed that the nickel catalyst, when complexed to the 4,7‐diOMephen ligand (**L4**), rapidly quenches the excited state of the photocatalyst (see Section S5.2). The rate constant for the quenching process was calculated to be 3.24 × 10^9^ M^−1^s^−1^, which is comparable to a recent literature value for the quenching of an excited state iridium catalyst by NiCl_2_(dtbpy) [44]. Interestingly, while the reactions thus far were prepared under an atmosphere of nitrogen as per our standard practice for parallel photoredox chemistry, an attempt to prepare **4** under air led to no reduction in yield. Although oxygen is a known quencher of the excited state of iridium photocatalysts, it is proposed that the catalyst is quenched more rapidly by the nickel complex, which is supported by the relatively large Stern‐Volmer and quenching constants measured [45]. These experiments, along with redox potentials of the catalysts and literature precedent for metallaphotoredox couplings, were used to postulate possible mechanisms for the reaction (see Scheme S2).

**SCHEME 5:**
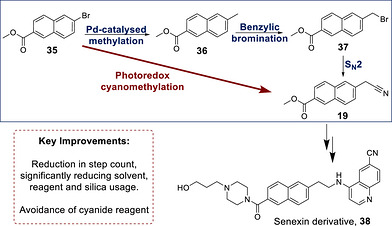
Route to Senexin derivative **38**.

Having explored the substrate scope of the reaction and aspects of the underpinning mechanism, the emerging process was applied to the synthesis of a Senexin derivative (**38**) (Scheme 5). Senexins are known inhibitors of mediator kinases CDK8 and CDK19 [46] and, therefore, Senexin‐based compounds are of interest in oncology research. The previously reported route to the Senexin compounds involves methylation of aryl bromide **35**, benzylic bromination to **37**, before S_N_2 displacement of bromide with cyanide to deliver key intermediate **19**. Direct cyanomethylation of **35** removes two steps from the synthetic route and obviates the use of a highly toxic cyanide reagent.

**SCHEME 6:**
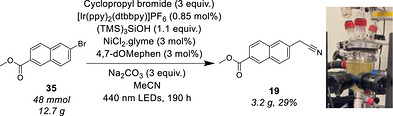
Cyanomethylation of bromide **35** on a 48 mmol scale to deliver intermediate **19** in 29% yield.

Cyanomethylation of **35** was carried out on a 48 mmol scale in a 500 mL jacketed lab reactor (Scheme 6). The reaction was externally irradiated by six 440 nm Kessil lamps, with mechanical overhead stirring and temperature control provided by a heater/chiller unit.

Although the reaction time significantly increased, likely due to limited light penetration, the desired product was prepared in 29% yield. The published route delivers **19** in 28% yield across three steps; application of this cyanomethylation reaction has achieved a comparable yield in a single step, significantly minimizing the waste profile, which is particularly substantial on larger scales. These benefits, alongside the avoidance of a toxic cyanide reagent, clearly demonstrate the value of this synthetic methodology. In addition, we gained further understanding around scaling heterogeneous batch photochemical systems, building on our previous work in this area [35, 47]. Mixing of liquid/solid/light systems is a key factor to be explored when developing a photochemical batch process for pilot plant scale.

## 3 Conclusion

In summary, we have developed a novel metallaphotoredox coupling for the synthesis of a valuable pharmacophore, the α‐aryl nitrile motif. Substantial optimization of the reaction was performed using HTE methods, resulting in a transformation which is tolerant of a range of functional groups and applicable to medicinally relevant substrates. The synthesis of these compounds was performed in a parallel manner, demonstrating the applicability of this methodology to library synthesis in an industrial context. The reaction has proved to be effective on scales from 30 µmol to 48 mmol, and was shown to offer a significant improvement to the synthesis of Senexin derivatives, in terms of both the length and safety profile of the route. Initial investigations into the mechanism of this reaction, alongside consideration of literature findings for similar metallaphotoredox couplings, have led to initial mechanistic hypotheses, with further elucidation ongoing in our laboratories.

## 4 Experimental Section

### 4.1 General Procedure for Cyanomethylation Reactions

Inside a nitrogen‐filled purge box, sodium carbonate (95.4 mg, 900 µmol, and 3.0 equiv.) was weighed under nitrogen into a 4 mL screw‐top borosilicate vial with a PTFE septum. Aryl halide (300 µmol and 1.0 equiv.) in acetonitrile (1 mL), and [Ir(dtbpy)(ppy)_2_]PF_6_ (2.3 mg, 2.55 µmol, and 0.0085 equiv.) in acetonitrile (0.2 mL), followed by tris(trimethylsilyl)silanol (102 µL, 330 µmol, and 1.1 equiv.) were then added to the vial. A solution of NiCl_2_.glyme (2.0 mg, 9.00 µmol, and 0.03 equiv.) and 4,7‐dimethoxy‐1,10‐phenanthroline (2.2 mg, 9.00 µmol, and 0.03 equiv.) in acetonitrile (1 mL) was pre‐mixed for 15 mins, then added to the vial, followed by cyclopropyl bromide (72 µL, 900 µmol, 3.0 equiv.). The vial was sealed, removed from the purge box and irradiated with 450 nm LEDs using a Lucent360 photochemical reactor. A Julabo F25 refrigerated/heating circulator containing Slytherm, set to 40°C, was used to maintain the temperature of the water bath. Stirring was set to 1000 rpm, and all LED panels were set to 100% intensity. Reactions were irradiated for 22–88 h.

### 4.2 Synthesis of 2‐(4‐acetylphenyl)acetonitrile, 4

The general procedure for reactions in Lucent360 was followed, irradiating for 22 h. The reaction mixture was diluted in DCM, adsorbed onto Florisil, and purified by column chromatography (10%–40% EtOAc in cyclohexane over 50 CV, 12 g RediSep Silica column). 1% EtOAc (10 CV) was flowed through the column prior to the gradient starting, in order to remove silanol‐related species. Fractions containing product were combined and solvent removed under reduced pressure at 30°C to give product as a pale yellow solid (25.8 mg, 50%). This compound was prepared from the corresponding aryl iodide, using the same general procedure (irradiating for 44 h) and purification process described above, to give the product as a yellow solid (2.1 mg, 4%). This compound was also prepared from the corresponding aryl chloride, using the same general procedure (irradiating for 88 h) and purification process described above, to give the product as a white powder (14.4 mg, 30%). LCMS (2 min high pH method): *t*_R_ = 0.74 min, [M‐H]^−^ 158, (93% purity). ^1^H NMR (400 MHz, CDCl_3_) δ ppm 7.98 (d, *J* = 8.5 Hz, 2H), 7.45 (d, *J* = 8.5 Hz, 2H), 3.82 (s, 2H), 2.61 (s, 3H). ^13^C NMR (101 MHz, CDCl_3_) δ ppm 197.2, 137.0, 135.0, 129.1, 128.2, 117.0, 26.6, and 23.7. Characterization data are consistent with literature reports [22].

### 4.3 Synthesis of 2‐(1‐oxo‐1,3‐dihydroisobenzofuran‐5‐yl)acetonitrile, 6

The general procedure for reactions in Lucent360 was followed, irradiating for 22 h. The reaction mixture was diluted in DCM, adsorbed onto Florisil, and purified by column chromatography (10%–50% EtOAc in cyclohexane over 60 CV, 12 g RediSep Silica column). Fractions containing product were combined and solvent removed under reduced pressure at 30°C to give a white solid (20.5 mg, 39%). LCMS (2 min high pH method): *t*_R_ = 0.63 mins [M‐H]^−^ 172, (98% purity). ^1^H NMR (400 MHz, CDCl_3_) δ ppm 7.93 (d, *J* = 7.9 Hz, 1H), 7.54–7.56 (m, 1H), 7.49–7.53 (m, 1H), 5.34 (s, 2H), 3.93 (s, 2H). ^13^C NMR (101 MHz, CDCl_3_) δ ppm 170.1, 147.7, 136.6, 129.1, 126.5, 125.9, 121.8, 116.8, 69.4, and 24.0. ṽ_max_ (CDCl_3_ solution) = 3063, 2950, 2914, 2252, 1752, 1621, 1410, 1052, 1001, 767, and 676 cm^−1^. HRMS (10 min formic acid): (C_10_H_6_NO_2_) [M‐H]^−^ requires 172.0404, found [M‐H]^−^ 172.0403 (error ‐0.58 ppm).

### 4.4 Synthesis of 2‐(3‐(trifluoromethyl)phenyl)acetonitrile, 7

The general procedure for reactions in Lucent360 was followed, irradiating for 22 h. The reaction mixture was diluted in DCM, adsorbed onto Florisil, and purified by column chromatography (0%–50% EtOAc in cyclohexane over 50 CV, 12 g RediSep Silica column). Fractions containing product were combined and solvent removed under reduced pressure at 30°C to give product as a clear, colorless liquid (20.1 mg, 34%). LCMS (2 min high pH method): *t*_R_ = 1.02 min, [M‐H]^−^ 184, (95% purity). ^1^H NMR (400 MHz, CDCl_3_) δ 7.63–7.51 (m, 4H), 3.82 (s, 2H). ^13^C NMR (101 MHz, CDCl_3_) δ 131.9, 131.3, 131.0, 129.8, and 125.04–125.17 (m), 124.8 (q, *J* = 3.1 Hz), 122.3, 116.9, 23.5. ^19^F NMR (376 MHz, CDCl_3_) δ ‐62.85. Characterization data are consistent with literature reports [22].

### 4.5 Synthesis of 2‐(4‐(*tert*‐butoxy)phenyl)acetonitrile, 8

The general procedure for reactions in Lucent360 was followed, irradiating for 88 h. The reaction mixture was diluted in DCM, adsorbed onto Florisil, and purified by column chromatography (1%–10% EtOAc in cyclohexane over 60 CV, 12 g RediSep Silica column, solid loaded). Fractions containing product were combined and solvent removed under reduced pressure at 30°C to give crude product. Crude product was purified by reverse‐phase column chromatography (30%–85% acetonitrile in 10 mM ammonium bicarbonate in water (adjusted to pH 10 with ammonia solution) over 10 mins at 40 mL/min, Waters XSelect CSH Prep C18 5 µm, 30 × 100 mm). Fractions containing product were combined, and acetonitrile was removed under reduced pressure at 30°C. Product was extracted into DCM and solvent removed under reduced pressure at 30°C to give product as a yellow oil (22.6 mg, 40%). LCMS (2 min high pH method): *t*_R_ = 1.07 min, [M+H]^+^ 190, (100% purity). ^1^H NMR (400 MHz, CDCl_3_) δ ppm 7.24 (d, *J *= 8.6 Hz, 2H), 7.01 (d, *J* = 8.6 Hz, 2H), 3.72 (s, 2H), 1.37 (s, 9H). ^13^C NMR (101 MHz, CDCl_3_) δ ppm 155.3, 128.5, 124.6, 124.5, 118.1, 78.8, 28.8, and 23.0. ṽ_max_ (CDCl_3_ solution) = 2978, 2933, 2250, 1610, 1507, 1238, 1160, and 894 cm^−1^. HRMS (10 min formic acid): (C_12_H_16_NO) [M+H]+ requires 190.1226, found [M+H]^+^ 190.1224 (error ‐1.05 ppm).

### 4.6 Synthesis of 2‐(4‐((1‐hydroxycyclopropyl)methyl)phenyl)acetonitrile, 9

The general procedure for reactions in Lucent360 was followed, irradiating for 66 h. The reaction mixture was diluted in DCM, adsorbed onto Florisil, and purified by column chromatography (5%–25% EtOAc in cyclohexane over 50 CV, 12 g RediSep Silica column, solid loaded). 1% EtOAc (10 CV) was flowed through the column prior to the gradient starting, in order to remove silanol‐related species. Fractions containing product were combined and solvent removed under reduced pressure at 30°C to give crude product. Crude product was purified by reverse‐phase column chromatography (15%–55% acetonitrile in 10 mM ammonium bicarbonate in water (adjusted to pH 10 with ammonia solution) over 10 mins at 40 mL/min, Waters XSelect CSH Prep C18 5 µm, 30 × 100 mm). Fractions containing product were combined, and acetonitrile was removed under reduced pressure at 30°C. Product was extracted into DCM and solvent removed under reduced pressure at 30°C to give product as off‐white crystals (13.0 mg, 23%). LCMS (2 min high pH method): *t*_R_ = 0.84 min, no mass ion observed, (99% purity). ^1^H NMR (400 MHz, CDCl_3_) δ ppm 7.26–7.34 (m, 4H), 3.73 (s, 2H), 2.88 (s, 2H), 1.84–1.91 (m, 1H), 0.76–0.90 (m, 2H), 0.58–0.70 (m, 2H). ^13^C NMR (101 MHz, CDCl_3_) δ ppm 138.8, 130.2, 128.2, 128.1, 117.9, 56.0, 43.7, 23.3, and 13.4. ṽ_max_ (CDCl_3_ solution) = 3306, 3215, 2910, 2247, 1515, 1436, 1288, 1014, and 740 cm^−1^. HRMS (10 min formic acid): (C_12_H_14_NO) [M+H]^+^ requires 188.1070, found [M+H]^+^ 188.1077 (error 3.72 ppm).

### 4.7 Synthesis of 2‐(4‐hydroxyphenyl)acetonitrile, 10

The general procedure for reactions in Lucent360 was followed, irradiating for 88 h. The reaction mixture was diluted in DCM, adsorbed onto Florisil, and purified by column chromatography (10%–25% EtOAc in cyclohexane over 50 CV, 12 g RediSep Silica column, solid loaded). Fractions containing product were combined and solvent removed under reduced pressure at 30°C to give product as a white powder (11.1 mg, 28%). LCMS (2 min high pH method): *t*_R_ = 0.62 min, [M‐H]^−^ 132 (100% purity). ^1^H NMR (400 MHz, CDCl_3_) δ ppm 7.15–7.20 (m, 2H), 6.81–6.86 (m, 2H), 5.21 (br s, 1H), 3.67 (s, 2H). ^13^C NMR (101 MHz, CDCl_3_) δ ppm 155.5, 129.3, 121.8, 118.2, 116.1, and 22.9. Characterization data are consistent with literature reports [45].

### 4.8 Synthesis of 2‐(2‐benzyl‐1,1‐dioxido‐3‐oxo‐3,4‐dihydro‐2*H*‐benzo[*e*][1,2,4]thiadiazin‐7‐yl)acetonitrile, 3

The general procedure for reactions in Lucent360 was followed, irradiating for 22 h. The reaction mixture was diluted in DCM, adsorbed onto Florisil, and purified by column chromatography (10%–50% EtOAc in cyclohexane over 50 CV, 12 g RediSep Silica column). Fractions containing product were combined and solvent removed under reduced pressure at 30°C to give product as a white powder (25.1 mg, 25%). LCMS (2 min high pH method): *t*_R_ = 0.70 min, [M‐H]^−^ 326, (98% purity). ^1^H NMR (400 MHz, DMSO‐d_6_) δ ppm 11.24–11.61 (br s, 1H), 7.76 (d, *J* = 2.0 Hz, 1H), 7.59 (dd, *J* = 8.4, 2.0 Hz, 1H), 7.12–7.23 (m, 6H), 4.86 (s, 2H), 4.01 (s, 2H). ^13^C NMR (101 MHz, DMSO‐d_6_) δ ppm 150.0, 136.9, 135.2, 134.5, 128.9, 128.2, 128.0, 127.4, 122.7, 122.0, 119.2, 118.5, 44.1, and 22.1. ṽ_max_ (CDCl_3_ solution) = 3257, 3067, 2925, 2256, 1699, 1507, 1334, 1169, 826, and 731 cm^−1^. HRMS (20 min high pH): (C_16_H_14_N_3_O_3_S) [M+H]^+^ requires 328.0750, found [M+H]^+^ 328.0741 (error ‐2.74 ppm).

### 4.9 Synthesis of 2‐(3‐(difluoromethoxy)phenyl)acetonitrile, 11

The general procedure for reactions in Lucent360 was followed, irradiating for 22 h. The reaction mixture was diluted in DCM, adsorbed onto Florisil, and purified by column chromatography (0%–30% EtOAc in cyclohexane over 50 CV, 12 g RediSep Silica column, solid loaded). Fractions containing product were combined and solvent removed under reduced pressure at 30°C to give product as a clear, colorless liquid (14.1 mg, 25%). LCMS (2 min high pH method): *t*_R_ = 0.91 min, [M‐H]‐ 182, (95% purity). ^1^H NMR (400 MHz, CDCl3) δ ppm 7.39–7.44 (m, 1H), 7.21–7.25 (m, 1H), 7.11–7.15 (m, 2H), 6.55 (t, *J* = 72.9 Hz, 1H), 3.79 (s, 2H). ^13^C NMR (151 MHz, CDCl_3_) δ ppm 151.5 (br d, *J *= 5.5 Hz), 131.9, 130.6, 124.9, 119.4, 119.3, 117.2, and 115.6 (t, *J* = 260.9 Hz), 23.5. ^19^F NMR (376 MHz, CDCl_3_) δ ppm ‐81.11 (d, *J* = 73.2 Hz, 1F). ṽ_max_ (CDCl_3_ solution) = 2923, 2254, 1613, 1592, 1490, 1382, 1244, 1116, 1037, 774, and 690 cm^−1^. HRMS (10 min formic acid): (C_10_H_9_N_2_O) [M‐H^−^] requires 173.0720, found [M‐H]^−^ 173.0415 (error ‐4.39 ppm).

### 4.10 Synthesis of 3‐(cyanomethyl)‐*N*‐methylbenzamide, 12

The general procedure for reactions in Lucent360 was followed, irradiating for 66 h. The reaction mixture was diluted in DCM, adsorbed onto Florisil, and purified by column chromatography (5%–50% EtOAc in cyclohexane over 50 CV, 12 g RediSep Silica column, solid loaded). Fractions containing product were combined and solvent removed under reduced pressure at 30°C to give product as a white solid (19.0 mg, 36% yield, 100% purity by LCMS). *t*_R_ = 0.64 min [M+H]^+^ 175, [M‐H]^−^ 173. ^1^H NMR (400 MHz, CDCl_3_) δ ppm 7.67 (br s, 1H), 7.56 (s, 1H), 7.44 (br d, *J* = 7.8 Hz, 1H), 7.30 (t, *J* = 7.8 Hz, 1H), 7.06 (br d, *J* = 7.8 Hz, 1H), 3.71 (s, 2H), 2.17 (s, 3H). ^13^C NMR (101 MHz, CDCl_3_) δ ppm 168.7, 138.8, 130.8, 129.8, 123.6, 119.4, 119.3, 117.8, 24.5, and 23.6. ṽ_max_ (CDCl_3_ solution) = 3265, 3106, 2920, 2247, 1662, 1596, 1559, 1439, 1371, 788, and 764, 690 cm^−1^. HRMS (10 min formic acid): (C_10_H_9_N_2_O) [M‐H]^−^ requires 173.0720, found [M‐H]^−^ 173.0718 (error ‐1.16 ppm).

### 4.11 Synthesis of 2‐(4‐fluoronaphthalen‐1‐yl)acetonitrile, 13

The general procedure for reactions in Lucent360 was followed, irradiating for 66 h. The reaction mixture was diluted in DCM, adsorbed onto Florisil, and purified by column chromatography (1%–5% EtOAc in cyclohexane over 50 CV, 12 g RediSep Silica column, solid loaded). Fractions containing product were combined and solvent removed under reduced pressure at 30°C to give crude product. Crude product was purified by reverse‐phase column chromatography (30%–85% acetonitrile in 10 mM ammonium bicarbonate in water (adjusted to pH 10 with ammonia solution) over 10 min at 40 mL/min, Waters XSelect CSH Prep C18 5 µm, 30 × 100 mm). Fractions containing product were combined, and acetonitrile was removed under reduced pressure at 30°C. Product was extracted into DCM and solvent removed under reduced pressure at 30°C to give product as off‐white crystals (12.3 mg, 22%). LCMS (2 min high pH method): *t*_R_ = 1.08 min, [M‐H]^−^ 184 (100% purity). ^1^H NMR (400 MHz, CDCl_3_) δ ppm 8.19 (dd, *J *= 8.2, 1.6 Hz, 1H), 7.87 (d, *J *= 8.4 Hz, 1H), 7.60–7.70 (m, 2H), 7.51 (dd, *J *= 8.0, 5.0 Hz, 1H), 7.13 (dd, *J *= 10.0, 8.0 Hz, 1H), 4.08 (s, 2H). ^13^C NMR (101 MHz, CDCl_3_) δ ppm 159.1 (d, *J *= 254.8 Hz), 132.0 (d, *J *= 4.6 Hz), 128.1, 126.7, 126.4 (d, *J *= 9.2 Hz), 124.1 (d, *J *= 16.8 Hz), 122.5 (d, *J *= 3.1 Hz), 121.7, 121.6, 117.5, 109.0 (d, *J *= 21.4 Hz), 21.4. ^19^F NMR (376 MHz, CDCl_3_) δ ‐121.72–‐121.63 (m). ṽ_max_ (CDCl_3_ solution) = 3069, 2913, 2254, 1604, 1466, 1397, 1260, 1053, 826, and 755 cm^−1^. HRMS (10 min formic acid): (C_12_H_7_FN) [M‐H]^−^ requires 184.0568, found [M‐H]^−^ 184.0602 (error 18.47 ppm).

### 4.12 Synthesis of *tert*‐butyl 5‐(cyanomethyl)isoindoline‐2‐carboxylate, 14

The general procedure for reactions in Lucent360 was followed, irradiating for 88 h. The reaction mixture was diluted in DCM, adsorbed onto Florisil, and purified by column chromatography (1%–10% EtOAc in cyclohexane over 60 CV, 12 g RediSep Silica column, solid loaded). Fractions containing product were combined and solvent removed under reduced pressure at 30°C to give product as an off‐white crystalline solid (26.1 mg, 33%). LCMS (2 min high pH method): *t*_R_ = 1.23 min, [M‐H]^−^ 257, [M‐Boc+H]^+^ 159 (98% purity). ^1^H NMR (600 MHz, DMSO‐*d*_6_, 298 K) δ ppm 7.67 (br s, 1H), 7.16 (br s, 1H), 7.11 (d, *J *= 8.4 Hz, 1H), 3.92 (s, 2H), 3.89 (t, *J *= 8.8 Hz, 2H), 3.05 (t, *J *= 8.8 Hz, 2H), 1.50 (br s, 9H). ^1^H NMR (600 MHz, DMSO‐*d*_6_, 373 K) δ ppm 7.59 (br d, *J *= 8.1 Hz, 1H), 7.17 (br s, 1H), 7.12 (br d, *J *= 7.3 Hz, 1H), 3.94 (t, *J *= 9.0 Hz, 2H), 3.88 (s, 2H), 3.08 (t, *J *= 8.6 Hz, 2H), 1.54 (s, 9H).^13^C NMR (101 MHz, DMSO‐*d*_6_) δ ppm 152.3, 142.5, 132.9, 127.3, 125.1, 125.0, 119.4, 114.7, 80.9, 48.1, 28.6, 27.2, and 22.4. ṽ_max_ (CDCl_3_ solution) = 2976, 2931, 2250, 1694, 1492, 1389, 1142, 1020, 821, and 765 cm^−1^. HRMS (10 min formic acid): (C_15_H_19_N_2_O_2_) [M‐*^t^* Bu+H]^+^ requires 203.0815, found [M‐*^t^* Bu+H]^+^ 203.0814 (error ‐0.49 ppm).

### 4.13 Synthesis of 2‐(2‐methylbenzo[*d*]thiazol‐5‐yl)acetonitrile, 15

The general procedure for reactions in Lucent360 was followed, irradiating for 22 h. The reaction mixture was diluted in DCM, adsorbed onto Florisil, and purified by column chromatography (10%–30% EtOAc in cyclohexane over 50 CV, 12 g RediSep Silica column). Fractions containing product were combined and solvent removed under reduced pressure at 30°C to give product as a white powder (20.2 mg, 35%). LCMS (2 min high pH method): *t*_R_ = 0.79 min, [M+H]^+^ 189, (99% purity). ^1^H NMR (400 MHz, CDCl_3_) δ ppm 7.89 (br s, 1H), 7.82 (d, *J* = 8.1 Hz, 1H), 7.32 (dd, *J* = 8.2, 1.6 Hz, 1H), 3.88 (s, 2H), 2.84 (s, 3H). ^13^C NMR (101 MHz, CDCl_3_) δ ppm 168.5, 153.9, 135.5, 128.0, 124.4, 122.1, 121.9, 117.7, 23.6, and 20.2. Characterization data are consistent with literature reports [22].

### 4.14 Synthesis of 2‐(quinolin‐6‐yl)acetonitrile, 16

The general procedure for reactions in Lucent360 was followed, irradiating for 22 h. The reaction mixture was dissolved in DCM, adsorbed onto Florisil, and purified by column chromatography (10%–100% EtOAc in cyclohexane over 50 CV, 12 g RediSep Silica column). 1% EtOAc (10 CV) was flowed through the column prior to the gradient starting, in order to remove silanol‐related species. Fractions containing product were combined and solvent removed under reduced pressure at 30°C to give product as a yellow gummy solid (15.3 mg, 29%). LCMS (2 min high pH method): *t*_R_ = 0.70 min, [M+H]^+^ 169, (97% purity). ^1^H NMR (400 MHz, CDCl_3_) δ ppm 8.92–8.97 (m, 1H), 8.10–8.20 (m, 2H), 7.84 (br s, 1H), 7.59–7.64 (m, 1H), 7.42–7.48 (m, 1H), 3.94–3.97 (m, 2H). ^13^C NMR (101 MHz, CDCl_3_) δ ppm 151.0, 147.7, 135.9, 130.6, 129.1, 128.3, 128.2, 126.7, 121.9, 117.4, and 23.7. Characterization data are consistent with literature reports [22].

### 4.15 Synthesis of 2‐(quinolin‐4‐yl)acetonitrile, 17

The general procedure for reactions in Lucent360 was followed, irradiating for 22 h. The reaction mixture was diluted in DCM, adsorbed onto Florisil, and purified by column chromatography (20%–30% EtOAc in cyclohexane over 50 CV, 12 g RediSep Silica column, solid loaded). 1% EtOAc (10 CV) was flowed through the column prior to the gradient starting, in order to remove silanol‐related species. Fractions containing product were combined and solvent removed under reduced pressure at 30°C to give crude product. Crude product was purified by reverse‐phase column chromatography (15%–55% acetonitrile in 10 mM ammonium bicarbonate in water (adjusted to pH 10 with ammonia solution) over 10 min at 40 mL/min, Waters XSelect CSH Prep C18 5 µm, 30 × 100 mm). Fractions containing product were combined, and acetonitrile was removed under reduced pressure at 30°C. Product was extracted into DCM and solvent removed under reduced pressure at 30°C to give product as a yellow solid (9.2 mg, 18%). LCMS (2 min high pH method): *t*_R_ = 0.76 min, [M+H]^+^ 169, (98% purity). ^1^H NMR (400 MHz, CDCl_3_) δ ppm 8.94 (d, *J* = 4.4 Hz, 1H), 8.19 (dd, *J* = 8.4, 1.9 Hz, 1H), 7.87 (dd, *J* = 8.4, 2.0 Hz, 1H), 7.88–7.82 (m, 1H), 7.63–7.70 (m, 1H), 7.56 (dt, *J* = 4.4, 1.0 Hz, 1H), 4.18 (d, *J* = 1.0 Hz, 2H). ^13^C NMR (101 MHz, CDCl_3_) δ ppm 150.3, 148.2, 135.5, 130.7, 130.0, 127.7, 125.9, 122.0, 120.7, 116.2, and 21.2. ṽ_max_ (CDCl_3_ solution) = 2931, 2253, 1511, 1395, 825, and 758 cm^−1^. HRMS (10 min formic acid): (C_11_H_7_N_2_) [M‐H]^−^ requires 167.0615, found [M‐H]^−^ 167.0614 (error ‐0.60 ppm).

### 4.16 Synthesis of 2‐(isoquinolin‐4‐yl)acetonitrile, 18

The general procedure for reactions in Lucent360 was followed, irradiating for 22 h. The reaction mixture was diluted in DCM, adsorbed onto Florisil and purified by column chromatography (20%–30% EtOAc in cyclohexane over 50 CV, 12 g RediSep Silica column, solid loaded). 1% EtOAc (10 CV) was flowed through the column prior to the gradient starting, in order to remove silanol‐related species. Fractions containing product were combined and solvent removed under reduced pressure at 30°C to give crude product. Crude product was purified by reverse‐phase column chromatography (15%–55% acetonitrile in 10 mM ammonium bicarbonate in water (adjusted to pH 10 with ammonia solution) over 10 min at 40 mL/min, Waters XSelect CSH Prep C18 5 µm, 30 × 100 mm). Fractions containing product were combined, and acetonitrile was removed under reduced pressure at 30°C. Product was extracted into DCM and solvent removed under reduced pressure at 30°C to give product as a yellow solid (11.4 mg, 22%). LCMS (2 min high pH method): *t*_R_ = 0.77 min, [M+H]^+^ 169, (98% purity). ^1^H NMR (400 MHz, CDCl_3_) δ ppm 9.27 (s, 1H), 8.58 (s, 1H), 8.05–8.08 (m, 1H), 7.92–7.96 (m, 1H), 7.83–7.88 (m, 1H), 7.69–7.74 (m, 1H), 4.08 (s, 2H). ^13^C NMR (101 MHz, CDCl_3_) δ ppm 153.9, 142.9, 133.5, 131.6, 128.7, 128.3, 127.9, 121.8, 119.9, 116.8, and 19.1. ṽ_max_ (CDCl_3_ solution) = 2934, 2904, 2242, and 748 cm^−1^. HRMS (10 min formic acid): (C_11_H_7_N_2_) [M‐H]^−^ requires 167.0615, found [M‐H]^−^ 167.0615 (error 0.00 ppm).

### 4.17 Synthesis of methyl 6‐(cyanomethyl)‐2‐naphthoate, 19

The general procedure for reactions in Lucent360 was followed, irradiating for 44 h. The reaction mixture was diluted in DCM, adsorbed onto Florisil, and purified by column chromatography (5%–15% EtOAc in cyclohexane over 50 CV, 12 g RediSep Silica column, solid loaded). 1% EtOAc (10 CV) was flowed through the column prior to the gradient starting, in order to remove silanol‐related species. Fractions containing product were combined and solvent removed under reduced pressure at 30°C to give crude product. Crude product was purified by reverse‐phase column chromatography (30%–85% acetonitrile in 10 mM ammonium bicarbonate in water (adjusted to pH 10 with ammonia solution) over 10 min at 40 mL/min, Waters XSelect CSH Prep C18 5 µm, 30 × 100 mm). Fractions containing product were combined, and acetonitrile was removed under reduced pressure at 30°C. Product was extracted into DCM and solvent removed under reduced pressure at 30°C to give product as an off‐white solid (14.5 mg, 21%). LCMS (2 min high pH method): *t*_R_ = 1.04 min, [M‐H]^−^ 224, (100% purity). ^1^H NMR (400 MHz, CDCl_3_) δ ppm 8.60 (br s, 1H), 8.10 (dd, *J* = 8.5, 1.7 Hz, 1H), 7.96 (d, *J* = 8.5 Hz, 1H), 7.85–7.89 (m, 2H), 7.45 (dd, *J* = 8.5, 1.8 Hz, 1H), 3.99 (s, 3H), 3.94 (s, 2H). ^13^C NMR (101 MHz, CDCl_3_) δ ppm 166.9, 135.4, 131.9, 130.8, 130.5, 129.8, 128.2, 128.0, 126.7, 126.3, 126.2, 117.4, 52.3, and 24.0. ṽ_max_ (CDCl_3_ solution) = 2961, 2933, 2257, 1725, 1294, 1180, and 804 cm^−1^. HRMS (10 min formic acid): (C_14_H_10_NO_2_) [M‐H]^−^ requires 224.0717, found [M‐H]^−^ 224.0726 (error 4.02 ppm).

### 4.18 Synthesis of methyl 6‐(cyanomethyl)picolinate, 20

The general procedure for reactions in Lucent360 was followed, irradiating for 22 h. The reaction mixture was diluted in DCM, adsorbed onto Florisil, and purified by column chromatography (10%–30% EtOAc in cyclohexane over 60 CV, 12 g RediSep Silica column, solid loaded). Fractions containing product were combined and solvent removed under reduced pressure at 30°C to give crude product. Crude product was purified by reverse‐phase column chromatography (30%–85% acetonitrile in 10 mM ammonium bicarbonate in water (adjusted to pH 10 with ammonia solution) over 10 min at 40 mL/min, Waters XSelect CSH Prep C18 5 µm, 30 × 100 mm). Fractions containing product were combined, and acetonitrile was removed under reduced pressure at 30°C. Product was extracted into DCM and solvent removed under reduced pressure at 30°C to give product as a colorless liquid (2.9 mg, 5.4%). LCMS (2 min high pH method): *t*_R_ = 0.59 min, [M+H]^+^ 177, (98% purity). ^1^H NMR (400 MHz, CDCl_3_) δ ppm 8.14 (d, *J *= 7.9 Hz, 1H), 7.95 (t, *J *= 7.9 Hz, 1H), 7.74 (d, *J *= 7.9 Hz, 1H), 4.09 (s, 2H), 4.04 (s, 3H). ^13^C NMR (101 MHz, CDCl_3_) δ ppm 165.0, 151.1, 148.3, 138.6, 125.4, 124.5, 116.6, 53.1, and 26.7. ṽ_max_ (CDCl_3_ solution) = 2955, 2924, 2253, 1724, 1590, 1321, 1138, 995, and 760 cm^−1^. HRMS (10 min formic acid): (C_9_H_9_N_2_O_2_) [M+H]^+^ requires 177.0659, found [M+H]^+^ 177.0664 (error 2.82 ppm).

### 4.19 Synthesis of methyl 2‐(cyanomethyl)benzoate, 21

The general procedure for reactions in Lucent360 was followed, irradiating for 22 h. The reaction mixture was diluted in DCM, adsorbed onto Florisil, and purified by column chromatography (1%–10% EtOAc in cyclohexane over 50 CV, 12 g RediSep Silica column, solid loaded). 1% EtOAc (10 CV) was flowed through the column prior to the gradient starting, to remove silanol‐related species. Fractions containing product were combined and solvent removed under reduced pressure at 30°C to give crude product. Crude product was purified by reverse‐phase column chromatography (30%–85% acetonitrile in 10 mM ammonium bicarbonate in water (adjusted to pH 10 with ammonia solution) over 12 min at 40 mL/min, Waters XSelect CSH Prep C18 5 µm, 30 × 100 mm). Fractions containing product were combined, and acetonitrile was removed under reduced pressure at 30°C. Product was extracted into DCM and solvent removed under reduced pressure at 30°C to give product as a white solid (14.1 mg, 27%). LCMS (2 min high pH method): *t*_R_ = 0.92 min, [M+H]^+^ 175, (99% purity). ^1^H NMR (400 MHz, CDCl_3_) δ ppm 8.05–8.09 (m, 1H), 7.58–7.59 (m, 1H), 7.57–7.58 (m, 1H), 7.40–7.46 (m, 1H), 4.22 (s, 2H), 3.93 (s, 3H). ^13^C NMR (101 MHz, CDCl_3_) δ ppm 166.7, 133.1, 132.1, 131.6, 130.2, 128.3 (2C), 117.9, 52.3, and 23.2. Characterization data are consistent with literature reports [46].

### 4.20 Synthesis of 2‐(2‐propoxyphenyl)acetonitrile, 22

The general procedure for reactions in Lucent360 was followed, irradiating for 22 h. The reaction mixture was diluted in DCM, adsorbed onto Florisil, and purified by column chromatography (0%–20% EtOAc in cyclohexane over 50 CV, 12 g RediSep Silica column, solid loaded). Fractions containing product were combined and solvent removed under reduced pressure at 30°C. The resulting material was dissolved in DMSO and purified by reverse‐phase column chromatography (30%–85% acetonitrile in 10 mM ammonium bicarbonate in water (adjusted to pH 10 with ammonia solution) over 50 CV, 13 g Biotage C18 column). Fractions containing product were combined, and acetonitrile was removed under reduced pressure at 30°C, leaving an aqueous solution. The product was extracted in DCM, and the DCM was removed under reduced pressure at 30°C to give the product as a clear, colorless liquid (10.1 mg, 19%). LCMS (2 min high pH method): *t*_R_ = 1.12 min, [M‐H]^−^ 174, (100% purity). ^1^H NMR (400 MHz, CDCl_3_) δ ppm 7.33–7.37 (m, 1H), 7.26–7.31 (m, 1H), 6.95 (td, *J* = 7.5, 1.2 Hz, 1H), 6.87 (dd, *J* = 8.1, 0.7 Hz, 1H), 3.97 (t, *J* = 6.4 Hz, 2H), 3.69 (s, 2H), 1.85 (sxt, *J* = 7.0 Hz, 2H), 1.07 (t, *J *= 7.4 Hz, 3H). ^13^C NMR (101 MHz, CDCl_3_) δ ppm 156.3, 129.5, 129.2, 120.6, 118.8, 118.1, 111.2, 69.7, 22.6, 18.7, and 10.6. ṽ_max_ (CDCl_3_ solution) = 2966, 2878, 2252, 1602, 1495, 1455, 1252, 980, and 751 cm^−1^. HRMS (20 min high pH): (C_11_H_12_NO) [M‐H]^−^ requires 174.0924, found [M‐H]^−^ 174.0880 (error ‐25.27 ppm).

### 4.21 Synthesis of 2‐(3‐chloro‐5‐isopropoxyphenyl)acetonitrile, 23

The general procedure for reactions in Lucent360 was followed, irradiating for 22 h. The reaction mixture was diluted in DCM, adsorbed onto Florisil, and purified by column chromatography (0%–10% EtOAc in cyclohexane over 50 CV, 12 g RediSep Silica column, solid loaded). Fractions containing product were combined and solvent removed under reduced pressure at 30°C to give crude product. Crude product was purified by reverse‐phase column chromatography (50%–99% acetonitrile in 10 mM ammonium bicarbonate in water (adjusted to pH 10 with ammonia solution) over 10 min at 40 mL/min, Waters XSelect CSH Prep C18 5 µm, 30 × 100 mm). Fractions containing product were combined, and acetonitrile was removed under reduced pressure at 30°C. Product was extracted into DCM and solvent removed under reduced pressure at 30°C to give product as a pale pink liquid (15.2 mg, 23%). LCMS (2 min high pH method): *t*_R_ = 1.23 min, no ionization observed (95% purity). ^1^H NMR (400 MHz, CDCl_3_) δ ppm 6.86–6.88 (m, 1H), 6.84 (t, *J *= 2.0 Hz, 1H), 6.73–6.76 (m, 1H), 4.53 (spt, *J *= 6.1 Hz, 1H), 3.66–3.69 (m, 2H), 1.33 (d, *J *= 6.1 Hz, 6H). ^13^C NMR (101 MHz, CDCl_3_) δ ppm 159.2, 135.6, 132.4, 120.0, 117.1, 115.6, 114.1, 70.7, 23.4, and 21.9. ṽ_max_ (CDCl_3_ solution) = 2979, 2931, 2256, 1577, 1452, 1270, 1114, 1016, 856, and 833 cm^−1^. HRMS (10 min formic acid): (C_11_H_11_ClNO) [M‐H]^−^ requires 208.0535, no mass ion found.

### 4.22 Synthesis of 4‐(cyanomethyl)benzenesulfonamide, 24

The general procedure for reactions in Lucent360 was followed, irradiating for 66 h. The reaction mixture was filtered and purified by reverse‐phase column chromatography (3%–22% methanol in 10 mM ammonium bicarbonate in water (adjusted to pH 10 with ammonia solution) over 14 min at 40 mL/min, Waters XBridge BEH C18 5 µm, 30 × 100 mm). Fractions containing product were combined, and methanol was removed under reduced pressure at 30°C. The aqueous solution was freeze‐dried to give the product as a white, fluffy solid (11.0 mg, 18%). LCMS (2 min high pH method): *t*_R_ = 0.47 min, [M‐H]^−^ 195, (98% purity). ^1^H NMR (400 MHz, DMSO‐*d*_6_) δ ppm 7.85 (d, *J* = 8.4 Hz, 2H), 7.55 (d, *J* = 8.4 Hz, 2H), 7.20–7.43 (br s, 2H), 4.17 (s, 2H). ^13^C NMR (101 MHz, DMSO‐*d*_6_) δ ppm 143.9, 135.8, 129.1, 126.8, 119.2, and 22.8. ṽ_max_ (CDCl_3_ solution) = 3361, 3254, 2251, 1568, 1337, and 1147 cm^−1^. HRMS (10 min formic acid): (C_8_H_7_N_2_O_2_S) [M‐H]^−^ requires 195.0234, found [M‐H]^−^ 195.0233 (error ‐0.51 ppm).

## Conflicts of Interest

The authors declare no conflicts of interest.

## Supporting information




**Supporting Information**: The authors have cited additional references within the Supporting Information [22, 33, 44].
