# Supplementary material for: Expedient Discovery of a Metallaphotoredox Cyanomethylation for Synthesizing α‐Aryl Nitriles
Source: Chemistry. 2026 Apr 1;32(23):e70946. doi: 10.1002/chem.70946 (PMC13282906; doi:10.1002/chem.70946)
Supplement: Supplementary file 1 — Supporting Information: The authors have cited additional references within the Supporting Information [22, 33, 44]. [file CHEM-32-e70946-s001.pdf]

Expedient Discovery of a Metallaphotoredox Cyanomethylation for Synthesizing α-Aryl Nitriles

Gemma C. Cook*[1,2], Blandine McKay[1], Craig Jamieson[2], Lee J. Edwards[3], Charlotte Harriman[1], Zackary S. Read[4], Simon T. Bate[4]

[1]Molecular Modalities Capabilities (MMC), GSK Medicines Research Centre, Gunnels Wood Road, Stevenage, Hertfordshire, SG1 2NY, UK

[2]Department of Pure and Applied Chemistry WestCHEM, University of Strathclyde, 295 Cathedral Street, Glasgow, G1 1XL, UK

[3]Drug Substance Development, Process Engineering & PAT, GSK Medicines Research Centre, Gunnels Wood Road, Stevenage, Hertfordshire, SG1 2NY, UK

[4]CMC Statistics, GSK Medicines Research Centre, Gunnels Wood Road, Stevenage, Hertfordshire, SG1 2NY, UK

Table of Contents

1. Materials 1
2. Analytical Methods 2
3. High-Throughout Experimentation (HTE) 8
   3.1 General Screening Procedure 8
   3.2 Screening of Discrete Variables 9
   3.3 Design of Experiments (DoE) campaign 27
   3.4 Screening of Continuous Variables 41
4. Preparative Reactions 43
   4.1 General Procedure for Reactions in Lucent360 43
   4.2 Determination of NMR Yield 43
   4.3 Determination of Purity and Isolated Yield 44
   4.4 Substrate Scope 44
5. Mechanistic Investigations 70
   5.1 Reactions performed in MeCN-d_3 70
   5.2 Stern-Volmer Quenching 75
6. NMR Spectra 86
7. References 109

1. Materials

Reagents were obtained commercially, from Sigma-Aldrich, Alfa Aesar and Combi-Blocks, and used as received. Solvents were anhydrous unless otherwise stated, and reactions were performed under an inert atmosphere in a nitrogen-filled MBRAUN glovebox or purgebox unless otherwise stated. Where stated, bases were weighed inside the glovebox using a Chronect weighing robot, which comprises of a Mettler-Toledo balance and Verulam robotic arm. The small-scale reaction vials used were as follows: 1.5 mL clear glass crimp neck vials, 11.6 × 32 mm, catalogue number: 548-0004, purchased from VWR, with the following lids: 11 mm natural rubber red-orange/butyl red/TEF, catalogue number 548-3261, purchased from VWR. Reactions run in the absence of light were run in the following vials: crimp vial, amber, wide mouth, with label and filling lines, 12×32 mm, catalogue number: 548-1203, purchased from VWR. The microwave vials used were as follows: 2-5 mL Biotage microwave vials, aluminium caps with septa and magnetic stirrer bars, catalogue number: 351521, purchased from Biotage. All photochemistry screens were carried out on Pacer's Photochemistry LED Illuminator (part number POS0349-0100-AS-B) using the continual wave (CW) function. The 24-well screening wavelength cassette (part number POS0358-0200-AS-A) and 48-well cassettes at 450 nm (part number POS0349-0600-AS-C) and 365 nm (part number POS0349-0400-AS-B). In screening mode, the reactor was placed onto an orbital shaker, with glass shaker beads placed into each vial (soda-lime glass, 3 mm diameter, purchased from Sigma-Aldrich, product code 1002768270). In scale-up mode, a standard hotplate stirrer was used with magnetic stirrers. The substrate scope reactions were performed in the Lucent360 photoreactor (part number HCK1021-01-001), equipped with 4 x 450 nm side light modules (part number HCK1021-01-015) and 1 x 450 nm bottom light module (part number HCK1021-01-019). Vials (4 mL screw-top borosilicate vial, purchased from VWR, part number 548-0051A) with a screw top lid (with silicone/PTFE septum, purchased from VWR, part number 548-0514A) were placed into the Lucent360 4 mL vial holder (part number HCK1021-01-

004). Screening experiments were recorded using the ACD notebook, Katalyst. [1] All other experiments were recorded in either eLNB or Signals notebooks.

2. Analytical Methods

Nuclear Magnetic Resonance (NMR)

NMR spectra were recorded using a Bruker AV400 instrument and processed using ACD/SpecManager v12.5. Chemical shifts (δ) are reported in parts per million (ppm) relative to tetramethylsilane (TMS) and coupling constants (*J*) are reported in Hz. The following abbreviations are used for multiplicities: s = singlet; br. s = broad singlet; d = doublet; t = triplet; q = quartet; quin = quintet; spt = septet; m = multiplet; dd = doublet of doublets; and td = triplet of doublets. NMR experiments were run at 30 °C.

Low resolution liquid chromatography mass spectrometry (LCMS)

2 minute formic acid method

These data were recorded using a Waters Acquity UPLC, equipped with a CSH C18 column (50 mm × 2.1 mm internal diameter, 1.7 µm packing diameter) at 40 °C.

The solvents employed were:

A = 0.1% v/v solution of formic acid in water

B = 0.1% v/v solution of formic acid in acetonitrile

The gradient employed was as follows:

**Time / min**	**Flow rate / mL min^{-1}**	**% A**	**% B**
0	1	97	3
1.5	1	3	97

1.9	1	3	97
2.0	1	97	3

UV detection was an averaged signal from wavelength of 210 nm to 450 nm and mass spectra were recorded on a Waters QDa mass spectrometer using alternate-scan positive and negative electrospray ionization (ES) within the mass range of 100-1000.

2 minute high pH method

These data were recorded using a Waters Acquity UPLC, equipped with a CSH C18 column (50 mm × 2.1 mm internal diameter, 1.7 µm packing diameter) at 40 °C.

The solvents employed were:

A = 10 mM ammonium bicarbonate in water adjusted to pH 10 with ammonia solution

B = acetonitrile

The gradient employed was as follows:

**Time / min**	**Flow rate / mL min^{-1}**	**% A**	**% B**
0	1	97	3
0.05	1	97	3
1.5	1	3	97
1.9	1	3	97
2.0	1	97	3

UV detection was an averaged signal from wavelength of 210 nm to 350 nm and mass spectra were recorded on a Waters QDa mass spectrometer using alternate-scan positive and negative electrospray ionization (ES) within the mass range of 100-1250.

6 minute formic acid method

These data were recorded using a Waters Acquity UPLC, equipped with a CSH C18 column (50 mm × 2.1 mm internal diameter, 1.7 µm packing diameter) at 40 °C.

The solvents employed were:

A = 0.1% v/v solution of formic acid in water

B = 0.1% v/v solution of formic acid in acetonitrile

The gradient employed was as follows:

**Time / min**	**Flow rate / mL min^{-1}**	**% A**	**% B**
0	0.8	90	10
3.90	0.8	20	80
4.00	0.8	10	90
4.70	0.8	5	95
4.80	0.8	97	3
5.10	0.5	97	3
5.50	0.2	97	3
6.00	0.2	97	3

UV detection was an averaged signal from wavelength of 210 nm to 450 nm and mass spectra were recorded on a Waters SQD2 mass spectrometer using alternate-scan positive and negative electrospray ionization (ES) within the mass range of 100-1250.

10 minute trifluoroacetic acid method

The UPLC analysis was conducted on an Acquity UPLC BEH C18 column (100 mm × 2.1 mm i.d. 1.7 μm packing diameter) at 50 °C.

The solvents employed were:

A = 0.1% v/v solution of TFA in water

B = 0.1% v/v solution of TFA in acetonitrile

The gradient employed was:

**Time / min**	**Flow rate / mL min^{-1}**	**% A**	**% B**
0	0.8	97	3
8.5	0.8	0.1	99.9
9	0.8	0.1	99.9
9.5	0.8	97	3
10	0.8	97	3

UV detection was an averaged signal from wavelength of 210 nm to 500 nm and mass spectra was recorded on a Waters Qda mass spectrometer using alternate-scan positive and negative electrospray ionization (ES).

High-resolution mass spectrometry (HRMS)

10 minute formic acid method

These data were recorded using a Waters Acquity UPLC, equipped with a CSH BEH C18 column (100 mm × 2.1 mm internal diameter, 1.7 µm packing diameter) at 50 °C.

The solvents employed were:

A = 0.1% v/v solution of Formic Acid in Water.

B = 0.1% v/v solution of Formic Acid in Acetonitrile.

The gradient employed was as follows:

**Time / min**	**Flow rate / mL min^{-1}**	**% A**	**% B**
0	0.8	95	5
8.5	0.8	7	93
9.0	0.8	7	93
9.5	0.8	95	5
10	0.8	95	5

UV detection was an averaged signal from wavelength of 210 nm to 500 nm and mass spectra were recorded on a Waters XEVO G2-XS QTof mass spectrometer using either positive or negative electrospray ionization (ES) within the mass range of 100-1200.

20 minute high pH method

These data were recorded using a Waters Acquity UPLC, equipped with a CSH BEH C18 column (100 mm × 2.1 mm internal diameter, 1.7 µm packing diameter) at 50 °C.

The solvents employed were:

A = 10 mM ammonium bicarbonate in water adjusted to pH 10 with ammonia solution

B = acetonitrile

The gradient employed was as follows:

Time / min	Flow rate / mL min^{-1}	% A	% B
0	0.8	99	1
0.5	0.8	99	1
17	0.8	10	90
18.5	0.8	10	90
19	0.8	99	1
20	0.8	99	1

UV detection was an averaged signal from wavelength of 210 nm to 500 nm and mass spectra were recorded on a Waters XEVO G2-XS QTof mass spectrometer using either positive or negative electrospray ionization (ES) within the mass range of 100-1200.

Infrared (FT-IR) spectroscopy

Infrared (FT-IR) spectra were obtained as evaporated films from $CDCl_3$ solutions on a Perkin Elmer Spectrum Two™ FT-IR spectrometer fitted with a Perkin Elmer UATR diamond sampling accessory. Absorption maxima ($\tilde{\nu}$) are listed in units of wavenumbers (cm^{-1}).

Purification by column chromatography

Normal phase silica chromatography for the substrate scope was performed using a Teledyne ISCO CombiFlash® Rf+ system, using pre-packed RediSep Rf Silver® SiO_2 columns. Dry loading was performed using Florisil® (60-100 mesh). The initial purification of compound **4** was carried out on a Biotage SP4,

using Biotage SNAP ultra-prepacked cartridges. UV response was monitored at wavelengths between 200 and 400 nm.

Quench Solution Preparation

N,N-dibenzylaniline (5.57 g, 2.0 mmol) was dissolved in dimethyl sulfoxide (3.45 mL, 40 mmol) and acetonitrile (1 L, 19 mol).

Calculation of P/IS values

The ratio of product to internal standard was calculated by dividing the LCMS peak area % for the product by the LCMS peak area % for *N,N*-dibenzylaniline.

3. High-Throughout Experimentation (HTE)

3.1 General Screening Procedure

Inside a nitrogen-filled purgebox, stock solutions of the reaction components were made as follows in acetonitrile: aryl bromide (0.3 M), photocatalyst (0.03 M), nickel catalyst and ligand (0.015 M) and alkylating agent (0.67 M). Base (60 µmol, 2.0 equiv.) was pre-weighed under nitrogen by a Chronect Quantos weighing robot into a 2 mL crimp-top HPLC vial. Aryl bromide solution (100 µL, 30 µmol, 1.0 equiv.) and photocatalyst solution (10 µL, 0.3 µmol, 0.01 equiv.), followed by tris(trimethylsilyl)silanol (14.6 µL, 45 µmol, 1.5 equiv.) were then added to the vial under nitrogen. The nickel and ligand solution (100 µL, 1.5 µmol, 0.05 equiv.) was pre-mixed for a minimum of 15 minutes, then added to the vial under nitrogen, followed by the alkylating agent solution (90 µL, 60 µmol, 2.0 equiv.). The vial was crimped and irradiated with an LED at 450 nm in the PHIL Pacer, with a light intensity of 300 mA and shaking using an orbital shaker at 150 rpm. The LED temperature typically reached 35 °C. After 17-19.5 hours,

15 μL of the crude reaction mixture and 35 μL quench solution was added to 150 μL of acetonitrile and analyzed by LCMS.

3.2 Screening of Discrete Variables

Control Reactions

General screening procedure was followed, where the base used was sodium carbonate, the photocatalyst used was $(Ir[dF(CF_3)ppy]_2(dtbpy))PF_6$, the aryl bromide used was 4-bromoacetophenone and the alkylating agent used was cyclopropyl bromide. Stock solutions were prepared for all reaction components as described in the general screening procedure, which were dosed across all reactions. Reactions were irradiated for 18 hours before quenching and analyzing by LCMS.

Control = all reaction components present.

Without light = all reaction components present, prepared in amber vial.

Without iridium = iridium solution replaced with 10 μL acetonitrile.

Without nickel = nickel and ligand solution replaced with 100 μL acetonitrile.

Without base = solutions added to empty vial rather than vial containing base.

Without silanol = silanol not added to reaction.

Without alkylating agent = cyclopropyl bromide solution replaced with 90 μL acetonitrile.

Table 1 Area % of each reaction product across a range of control reactions.

	Area % by LCMS					Product/ Internal Standard Ratio
	nitrile product ***4***	*des-bromo starting material* ***4c***	*bromide starting material* ***5***	*alkylated product* ***4a***	*starting material dimer* ***4b***	
**Control**	13.9	25.8	0.0	6.2	31.2	2.8
**Without light**	0.0	0.0	63.6	0.0	0.0	0.0
**Without iridium**	0.0	0.0	65.7	0.0	0.0	0.0
**Without nickel**	0.0	58.8	4.7	1.3	3.8	0.0
**Without silanol**	0.0	0.0	79.6	0.0	0.0	0.0
**Without base**	7.4	19.6	0.0	4.8	23.2	0.3
**Without alkyl bromide**	8.6	23.2	2.4	0.0	24.1	0.8

Investigations into dehalogenation process

It was noticed from the initial control reaction that dehalogenation became prevalent when the nickel catalyst was excluded from the reaction conditions. The cause of dehalogenation was further investigated to facilitate optimization and minimize the des-bromo side product.

Control Reactions II

General screening procedure was followed, where the base used was sodium carbonate, the photocatalyst used was (Ir[dF(CF_3)ppy]$_2$(dtbpy))PF_6, the aryl bromide used was 4-bromoacetophenone and the alkylating agent used was cyclopropyl bromide. Stock solutions were prepared for all reaction components as described in the general screening procedure, which were dosed across vial according to the screen design (Table 2). Reactions were irradiated at each wavelength according to the screen design for 18 hours before quenching and analyzing by LCMS.

Control = all reaction components present.

Without nickel = nickel solution replaced with 100 μL acetonitrile.

Without iridium or nickel = iridium solution and nickel and ligand solution replaced with 110 μL acetonitrile.

Without alkylating agent = cyclopropyl bromide solution replaced with 90 μL acetonitrile.

*Table 2 Ratio of de-brominated starting material **4c** to internal standard after reacting in the absence of certain reaction components and irradiating at a range of different wavelengths.*

	**Wavelength (nm)**					
	**365**	**385**	**405**	**420**	**450**	**525**
without Ni	3.3	4.3	4.1	4.2	4.3	0.9
without Ni or Ir	1.3	4.1	1.2	0.0	0.0	0.0
control	1.9	1.4	1.4	1.2	1.5	0.5
without alkylating agent	2.0	1.4	1.5	1.6	1.7	0.7

The results of this screen suggest that dehalogenation could be a light-mediated process, which is photosensitized by the iridium photocatalyst.

Control Reactions III

General screening procedure was followed, where the base used was sodium carbonate, the photocatalyst used was (Ir[dF(CF_3)ppy]$_2$(dtbpy))PF_6, the aryl bromide used was 4-bromoacetophenone and the alkylating agent used was cyclopropyl bromide. Stock solutions were prepared for the photocatalyst, nickel and ligand as described in the general screening procedure. The solution of cyclopropyl bromide (60 μmol, 2.0 equiv.) was prepared in acetonitrile (45 μL). A solution of tris(trimethylsilyl)silanol (45 μmol, 1.5 equiv.) was prepared in acetonitrile (45 μL). Stock solutions were dosed across all reactions according to

the specifications listed below. Reactions were irradiated for 17 hours before quenching and analyzing by LCMS.

Control reaction at 365 nm = all reaction components present but irradiated at 365 nm instead of 450 nm.

Without iridium at 365 nm = iridium solution replaced with 10 µL acetonitrile, irradiated at 365 nm instead of 450 nm.

Control reaction at 450 nm = all reaction components present and irradiated at 450 nm.

*Table 3 Ratio of product **4** to internal standard and product to des-bromo starting material **4c** after reacting in the absence of certain reaction components and irradiating at either 365 nm or 450 nm.*

Condition	product/ internal standard	product/ des bromo
365 nm control	1.9	1.4
365 nm without Ir	2.8	0.6
450 nm control	1.7	1.1

This screen demonstrated that the reaction can occur under photocatalyst-free conditions under UV irradiation. However, dehalogenation occurred to an even greater extent, therefore these conditions were not taken forward.

Alkylating Agent Screen

General screening procedure was followed, where the base used was sodium carbonate, photocatalyst used was (Ir[dF(CF_3)ppy]$_2$(dtbpy))PF_6 and the aryl bromide used was 4-bromoacetophenone. Stock solutions were prepared for all reaction components as described in the general screening procedure, which were dosed across all reactions. Reactions were irradiated for 17 hours before quenching and analyzing by LCMS.

Alkylating agents tested: cyclopropyl bromide, cyclopropylmethyl bromide, chloromethyl cyclopropane, (iodomethyl)cyclopropane, 2-bromo-2-methyl propane, benzyl bromide, 2-bromocyclopropane-1-carboxylic acid, 3-bromooxetane, 1-bromo-2-methylcyclohexane, 2-bromopropane, 2-chloropropane and 2-iodopropane.

Without alkylating agent = alkylating agent solution replaced with 90 µL acetonitrile.

Table 4 Ratio of product ***4*** *to internal standard and product to des-bromo starting material* ***4c*** *after reacting with different alkylating agents (****A1*** *to* ***A12****). Screens 1 and 2 were carried out on different days therefore differences in product/ internal standard and product / des-bromo values are expected due to variations in the LCMS from day-to-day. For this reason, a control reaction with cyclopropyl bromide was included in both screens, so the results of each alkylating agent should be compared to this value rather than across the whole table.*

**Screen**	**Alkylating agent**	**product/ internal standard**	**product/ des bromo**
	**none**	1.12	0.44
	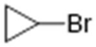 **A1**	1.89	0.99
	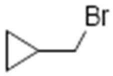 **A2**	0.00	0.00
**1**	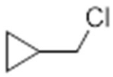 **A3**	0.00	0.00
	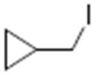 **A4**	0.00	0.00
	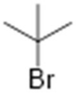 **A5**	0.49	0.13
	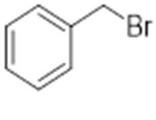	0.84	0.20

	**A6**		
	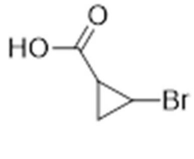 **A7**	0.25	0.50
	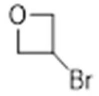 **A8**	1.02	0.19
	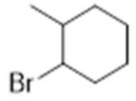 **A9**	0.65	0.34
**2**	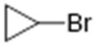 **A1**	2.53	1.84
	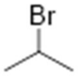 **A10**	0.64	0.30
	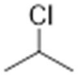 **A11**	1.23	0.30
	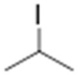 **A12**	1.01	9.28

Photocatalyst & Base Screen

General screening procedure was followed, where the aryl bromide used was 4-bromoacetophenone. The alkylating agent used was cyclopropyl bromide. Stock solutions were prepared for all reaction

components as described in the general screening procedure, which were dosed across the vials according to Table 5. Reactions were irradiated for 17 hours before quenching and analyzing by LCMS.

Bases tested = sodium carbonate, sodium acetate, potassium carbonate, 2,6-lutidine, triethylamine and *N,N*-diisopropylethylamine.

Photocatalysts tested = $(Ir[dF(CF_3)ppy]_2(dtbpy))PF_6$, $Ru(phen)_3Cl_2$, $[Ru(bpz)_3][PF_6]_2$, $[Ir(ppy)_2(dtbpy)]PF_6$, fluorescein, 9-mesityl-10-methylacridinium tetrafluoroborate, *fac*-$Ir(ppy)_3$, 2,4,6-tris(diphenylamino)-5- fluoroisophthalonitrile.

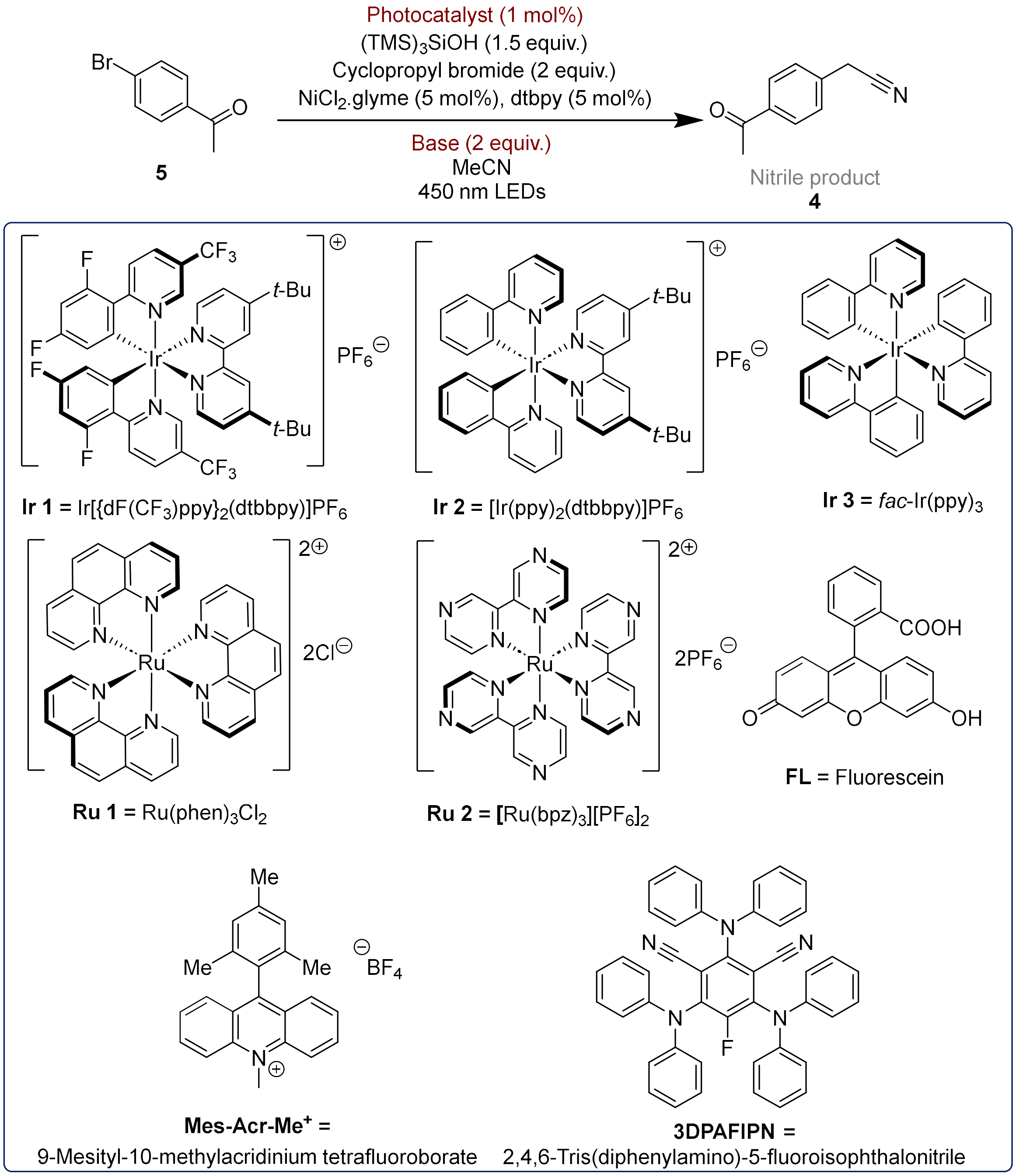

Figure 1 Catalyst systems used in Table 5.

*Table 5 Ratio of product **4** to internal standard (0.7 μmol) when various photocatalysts and bases are employed in the coupling of 4-bromoacetophenone with acetonitrile. Standard reaction conditions: bromoacetophenone (30 μmol, 1.0 equiv.), photocatalyst (0.3 μmol, 0.01 equiv.), nickel chloride ethylene glycol dimethyl ether complex (1.5 μmol, 0.05 equiv.), 4,4'-di-tert-butyl-2,2'-dipyridyl (1.5 μmol, 0.05 equiv.), tris(trimethylsilyl)silanol (45 μmol, 1.5 equiv.), cyclopropyl bromide (60 μmol, 2.0 equiv.), base (60 μmol, 2.0 equiv.) in acetonitrile (0.1 M), irradiating with 450 nm LEDs.*

	Ir 1	Ru 1	Ru 2	Ir 2	FL	Mes-Acr-Me+	Ir 3	3DPAF IPN
sodium carbonate	2.23	0.00	0.00	2.22	1.83	1.8	0.00	2.84
sodium acetate	2.87	0.00	0.00	3.77	0.77	0.72	0.69	1.46
potassium carbonate	2.09	0.00	0.00	1.84	0.52	1.48	0.00	1.57
2,6-lutidine	1.6	0.00	0.25	2.61	2.09	1.23	0.00	2.45
TEA	3.14	0.00	0.00	0.73	0.82	1.21	0.57	0.00
DIPEA	0.00	0.00	0.00	0.00	0.00	0.66	0.84	0.00

Low product/internal standard ratio — High product/internal standard ratio

No clear trends were evident between reaction success and the redox potentials or triplet energies of the catalysts, nor the pka_H of the base.[2-4] TEA and DIPEA are able to quench the excited state of some photocatalysts, which likely explains the lack of product formation in these reactions. [5]

Nickel source & ligand Screen

General screening procedure was followed, where the aryl bromide used was 4-bromoacetophenone and the alkylating agent used was cyclopropyl bromide. Stock solutions were prepared for all reaction components as described in the general screening procedure, which were dosed across the vials according to the screen design (Table 6). Reactions were irradiated for 16 hours before quenching and analyzing by LCMS.

Nickel sources tested = $NiCl_2$.glyme, $NiBr_2$.glyme, $NiBr_2.3H_2O$, $NiCl_2$, $Ni(OTf)_2$, $NiCl_2(PPh_3)_2$, $NiCl_2(PCy_3)_2$.

Ligands tested = dtbpy, bpy, phen, 4,7-dimethoxy phen, 4,4'-dimethyl bpy, 4,4'-dimethoxy bpy.

*Table 6 Ratio of product **4** to internal standard when various nickel sources and ligands are employed to the coupling of 4-bromoacetophenone **5** with acetonitrile.*

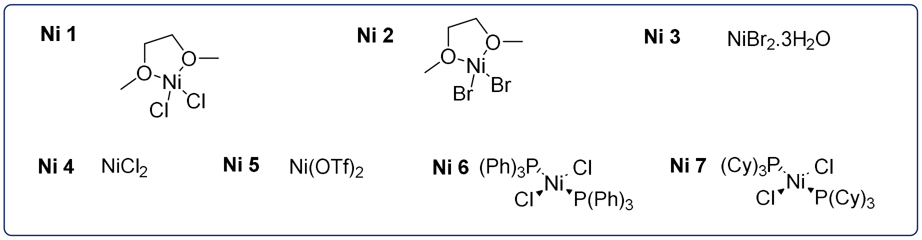

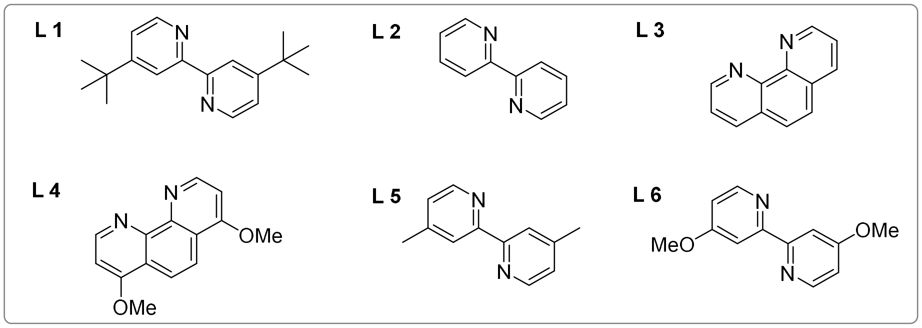

	Ni 1	Ni 2	Ni 3	Ni 4	Ni 5	Ni 6	Ni 7
L1	2.20	3.63	3.27	4.89	4.49	1.85	2.18
L2	4.83	2.69	3.45	5.08	4.62	2.95	1.59
L3	4.09	4.20	3.19	4.09	2.53	2.45	2.18
L4	7.40	6.40	6.78	4.97	4.43	3.16	1.69
L5	4.87	3.60	3.47	4.96	3.15	1.49	3.87
L6	4.01	3.49	3.16	5.16	2.51	2.60	5.79

Library Validation Experiments (LVEs)

General screening procedure was followed. Stock solutions were prepared for all reaction components as described in the general screening procedure, which were dosed across the vials according to the screen designs shown in Table 7 (LVE 1), Table 8 (LVE 2), Ratio of product to internal standard for 6 different substrates when subjected to each combination of best performing photocatalysts, bases and nickel ligands (LVE 3). Reactions were irradiated for 18 hours (LVE 1 and 3) or 17 hours (LVE 2) before quenching and analyzing by LCMS.

Table 7 Ratio of product to internal standard for 6 different substrates when subjected to each combination of best performing photocatalysts, bases and nickel ligands.

Substrate	Base	Ir 1		Ir 2	
		dtbpy	4,7-dimethoxy phen	dtbpy	4,7-dimethoxy phen
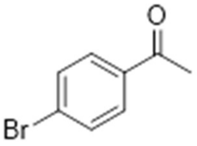 **5**	Na_2CO_3	3.14	6.18	4.25	6.73
	NaOAc	2.82	3.03	3.34	2.19
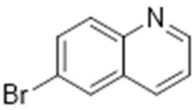 **39**	Na_2CO_3	3.42	3.77	4.05	3.35
	NaOAc	2.51	1.84	2.89	2.46
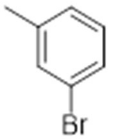 **40**	Na_2CO_3	0.47	0.69	0.35	0.74
	NaOAc	0.33	0.00	0.00	0.00
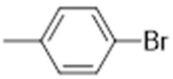 **41**	Na_2CO_3	1.32	1.25	1.40	1.50
	NaOAc	0.55	0.32	0.67	0.33
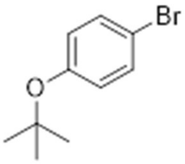 **42**	Na_2CO_3	1.33	1.68	1.10	1.67
	NaOAc	0.56	0.22	0.43	0.38
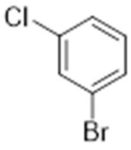 **43**	Na_2CO_3	0.20	0.58	0.29	0.71
	NaOAc	0.19	0.22	0.25	0.19

Table 8 Ratio of product to internal standard for 6 different substrates when subjected to each combination of best performing photocatalysts and nickel ligands, with sodium carbonate as base.

**Substrate**	**Ir 1**		**Ir 2**	
	**dtbpy**	**4,7-dimethoxy phen**	**dtbpy**	**4,7-dimethoxy phen**
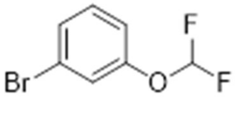 **44**	0.75	0.41	0.40	0.51
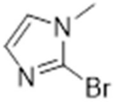 **45**	0.55	0.58	0.76	0.76
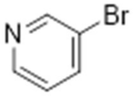 **46**	0.34	0.41	0.69	0.94
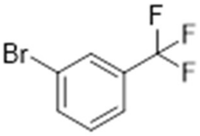 **47**	0.29	0.37	0.41	0.40
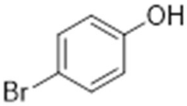 **48**	0.53	0.38	0.34	0.23
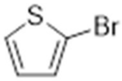 **49**	0.22	0.34	0.02	0.09

Table 9 Ratio of product to internal standard for 6 different substrates when subjected to each combination of best performing photocatalysts, bases and nickel ligands.

**Substrate**	**Base**	**Ir 1**		**Ir 2**	
		**dtbpy**	**4,7-dimethoxy phen**	**dtbpy**	**4,7-dimethoxy phen**
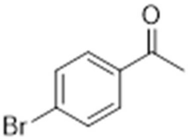 **5**	Na_2CO_3	2.39	5.04	3.66	5.28
	TEA	0.00	0.00	0.00	0.00

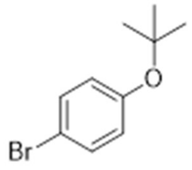 **42**	Na_2CO_3	1.23	1.72	1.37	1.60
	TEA	0.00	0.00	0.00	0.00
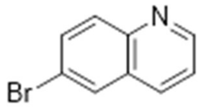 **39**	Na_2CO_3	4.19	4.34	3.38	5.30
	TEA	1.23	0.00	0.00	0.00
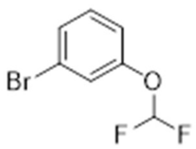 **44**	Na_2CO_3	0.25	0.33	0.48	0.64
	TEA	0.00	0.00	0.00	0.00
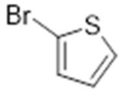 **49**	Na_2CO_3	0.55	0.54	0.51	0.33
	TEA	0.00	0.00	0.00	0.00
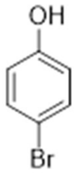 **48**	Na_2CO_3	0.43	0.63	0.79	0.99
	TEA	0.67	1.20	1.35	0.83

Investigation into the reproducibility across the screening plate

Data from the LVE experiments prompted an investigation into how much variation in product/internal standard ratio should be expected from well to well, in order to put any differences observed into the context of the anticipated standard error. The source of variation was identified to be the heterogeneity of the nickel catalyst stock solution; when the concentration of this solution was decreased, variation across the plate reduced. The LCMS analysis, consistency of light intensity across the plate, and the scale of the reaction were also examined as possible causes of variation.

Reproducibility Across Reaction Plate

General screening procedure was followed. The base used was sodium carbonate, photocatalyst used was $[Ir(ppy)_2(dtbpy)]PF_6$, the alkylating agent used was cyclopropyl bromide, the nickel source used was $NiCl_2$.glyme, the ligand used was 4,7-dimethoxy phen and the aryl bromide used was 4-bromoacetophenone. Stock solutions were prepared for all reaction components as described in the general screening procedure, which were dosed across all reactions. Reactions were irradiated for 18 hours before quenching and analyzing by LCMS. Excel was used to calculate standard deviation, mean and standard error. Outliers were identified as values below the mean minus two standard deviations (mean-2STDV), or above the mean plus two standard deviations (mean+2STDV).

*Table 10 Analysis of variation in product **4** area %, internal standard area % and the resulting product to internal standard (P/IS) ratio, measured by LCMS, across 48 identical reactions. P/IS values which are statistical outliers are shown in red.*

Product Area %

	1	2	3	4	5	6	7	8
**A**	20.39	21.79	22.12	25.63	25.14	11.89	25.92	23.19
**B**	29.85	23.67	23.52	28.60	27.75	28.22	24.52	28.46
**C**	29.5	27.07	23.61	28.86	27.89	31.35	31.29	25.97
**D**	27.51	28.51	31.47	29.30	19.61	17.27	23.21	18.79
**E**	29.73	27.51	27.28	26.21	25.30	25.01	25.90	28.32
**F**	34.53	23.15	21.58	26.81	27.54	28.93	28.44	22.80

Internal Standard Area %

	1	2	3	4	5	6	7	8
**A**	5.02	5.29	5.30	5.04	5.34	5.12	5.02	4.86
**B**	5.34	4.96	4.95	5.34	5.54	5.33	5.27	5.6
**C**	5.37	5.08	5.06	5.44	5.44	5.67	5.73	5.13
**D**	5.66	5.79	5.8	5.53	5.10	5.13	5.4	4.77
**E**	5.51	5.07	5.14	5.08	5.08	5.15	5.48	5.39
**F**	6.10	4.91	4.99	5.39	5.32	5.41	5.45	5.23

Product/Internal Standard Ratio

	1	2	3	4	5	6	7	8
**A**	4.06	4.12	4.17	5.09	4.71	**2.32**	5.16	4.77
**B**	5.59	4.77	4.75	5.36	5.01	5.29	4.65	5.08
**C**	5.49	5.33	4.67	5.31	5.13	5.53	5.46	5.06

**D**	4.86	4.92	5.43	5.30	3.85	**3.37**	4.30	3.94
**E**	5.40	5.43	5.31	5.16	4.98	4.86	4.73	5.25
**F**	5.66	4.71	4.32	4.97	5.18	5.35	5.22	4.36

Reproducibility Across LCMS Plate

A stock solution of 4-bromoacetophenone (36.0 mg, 181 µmol), (4-acetylphenyl)acetonitrile (28.8 mg, 181 µmol), *N,N*-dibenzylaniline (50.0 mg, 181 µmol) was prepared in acetonitrile (28.69 mL). To three separate 96-well LCMS plates, stock solution (200 µL) was added to 48 of the wells. Each plate was analyzed using the 2 minute formic acid method on a different LCMS instrument. The reproducibility was assessed by comparing P/IS values across each plate and between the three instruments.

*Table 11 Product **4** to internal standard ratios for each well of a 96-well plate containing identical stock solution, analyzed across 3 different LCMS machines.*

LCMS 1

	**1**	**2**	**3**	**4**	**5**	**6**	**7**	**8**	**9**	**10**	**11**	**12**
**A**	0.63	0.64	0.63	0.63	0.63	0.63	0.63	0.62	0.64	0.63	0.64	0.63
**B**	0.62	0.63	0.63	0.62	0.63	0.63	0.63	0.64	0.63	0.64	0.63	0.64
**C**	0.62	0.63	0.63	0.63	0.63	0.62	0.63	0.64	0.64	0.64	0.64	0.63
**D**	0.63	0.63	0.64	0.63	0.64	0.64	0.64	0.63	0.63	0.63	0.64	0.64
**E**	0.64	0.63	0.64	0.63	0.64	0.63	0.64	0.64	0.63	0.64	0.64	0.62
**F**	0.63	0.63	0.63	0.62	0.64	0.64	0.64	0.64	0.64	0.63	0.64	0.64
**G**	0.64	0.63	0.64	0.64	0.64	0.64	0.64	0.63	0.63	0.64	0.63	0.62
**H**	0.63	0.64	0.63	0.63	0.64	0.62	0.63	0.63	0.63	0.63	0.64	0.64

LCMS 2

	**1**	**2**	**3**	**4**	**5**	**6**	**7**	**8**	**9**	**10**	**11**	**12**
**A**	0.67	0.67	0.67	0.67	0.67	0.67	0.67	0.67	0.67	0.67	0.67	0.67
**B**	0.67	0.67	0.67	0.67	0.67	0.67	0.67	0.67	0.67	0.67	0.67	0.67
**C**	0.67	0.67	0.67	0.67	0.67	0.67	0.67	0.67	0.67	0.67	0.67	0.67
**D**	0.67	0.67	0.67	0.67	0.67	0.67	0.67	0.67	0.67	0.67	0.67	0.67
**E**	0.67	0.67	0.67	0.67	0.67	0.67	0.67	0.67	0.67	0.67	0.67	0.67
**F**	0.67	0.67	0.67	0.67	0.67	0.67	0.67	0.67	0.67	0.67	0.67	0.67
**G**	0.67	0.67	0.67	0.67	0.67	0.67	0.67	0.67	0.67	0.67	0.67	0.67
**H**	0.67	0.67	0.67	0.67	0.67	0.67	0.67	0.67	0.67	0.67	0.67	0.67

LCMS 3

	1	2	3	4	5	6	7	8	9	10	11	12
**A**	0.86	0.85	0.80	0.75	0.74	0.72	0.83	0.82	0.75	0.74	0.82	0.73
**B**	0.81	0.86	0.83	0.77	0.81	0.76	0.81	0.76	0.81	0.80	0.75	0.71
**C**	0.72	0.84	0.76	0.84	0.81	0.83	0.72	0.75	0.86	0.78	0.75	0.81
**D**	0.79	0.82	0.73	0.75	0.77	0.86	0.72	0.77	0.82	0.85	0.83	0.74
**E**	0.82	0.85	0.75	0.77	0.82	0.77	0.81	0.82	0.70	0.85	0.78	0.82
**F**	0.79	0.82	0.77	0.73	0.87	0.85	0.82	0.81	0.73	0.77	0.85	0.74
**G**	0.81	0.90	0.73	0.80	0.76	0.73	0.83	0.80	0.73	0.71	0.86	0.84
**H**	0.79	0.84	0.76	0.84	0.73	0.83	0.74	0.86	0.86	0.86	0.88	0.74

Light Intensity Measurements

The intensity of each LED of the 450 nm 48-LED cassette was measured as resistance (high resistance correlates to low light intensity) using LightMap V.1 software (Table12).[6] The LEDs were operated at a current of 304 mA during the measurement. The output number does not directly read the Light Dependent Resistor (LDR) but gives repeatable readings on an arbitrary scale. The LightMap equipment was modified from the original design (Figure 2) to the modified design (Figure 3) to give better resolution of voltage at higher light levels.

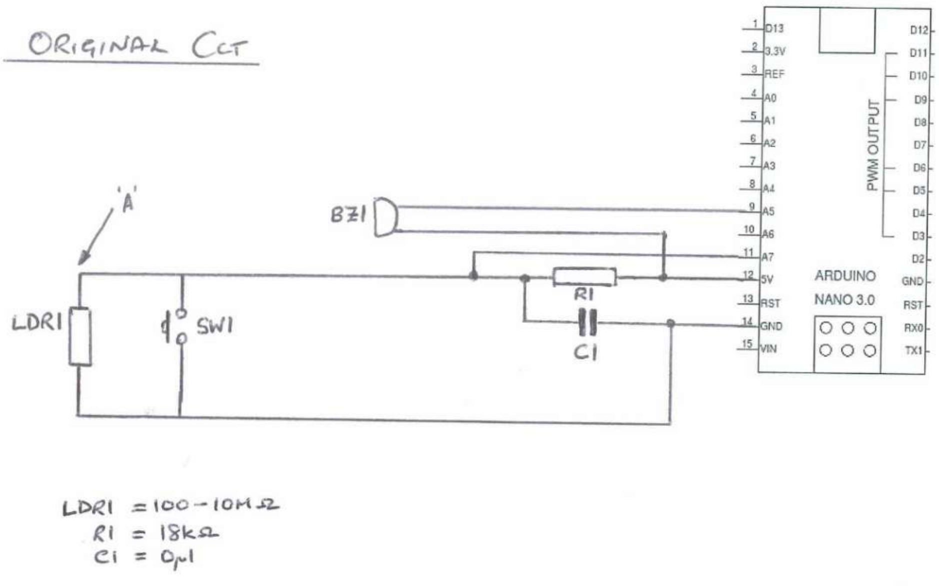

Figure 2 Original circuit diagram, reproduced from SourceForge.net. [6]

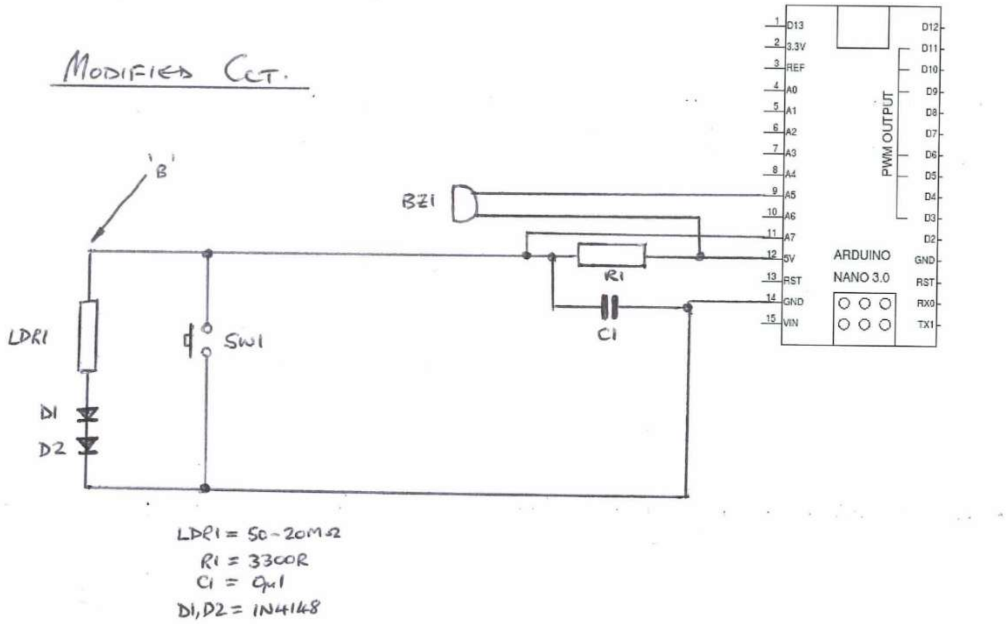

Figure 3 Modified circuit diagram.

Table 12 Light intensity values (Ω) of each LED used to irradiate the reactions.

	**1**	**2**	**3**	**4**	**5**	**6**	**7**	**8**
**A**	8223	8294	8283	8234	8238	8245	8256	8253
**B**	8227	8238	8215	8182	8204	8186	8223	8197
**C**	8171	8189	8204	8182	8174	8212	8208	8163
**D**	8245	8279	8197	8160	8193	8230	8193	8174
**E**	8148	8160	8160	8178	8264	8178	8145	8174
**F**	8152	8156	8182	8171	8130	8134	8141	8137

Reproducibility Across Reaction Plate – Increasing Reaction Scale

General screening procedure was followed, where the micromoles of the aryl bromide was increased from 30 to 150 µmoles (Table 13) and equivalents of catalysts and reagents were kept constant by increasing quantities accordingly. The base used was sodium carbonate, photocatalyst used was $[Ir(ppy)_2(dtbpy)]PF_6$, the alkylating agent used was cyclopropyl bromide, the nickel source used was $NiCl_2$.glyme, the ligand used was 4,7-dimethoxy phen and the aryl bromide used was 4-bromoacetophenone. Stock solutions were prepared for all reaction components as described in the general screening procedure, which were dosed across all reactions. Reactions were irradiated for 18 hours before quenching and analyzing by LCMS.

Table 13 Scales and corresponding reaction volumes tested in reproducibility experiment.

Plate Row	Scale (µmoles)	Reaction volume (µL)
A	30	300
B	40	400
C	60	600
D	90	900
E	120	1200
F	150	1500

Excel was used to calculate standard deviation, mean and standard error. Outliers were identified as values below the mean minus two standard deviations (mean-2STDV), or above the mean plus two standard deviations (mean+2STDV).

Table 14 Analysis of variation in product ***4*** *to internal standard (P/IS) ratio across 8 identical reactions at 6 different scales. P/IS values which are statistical outliers within each row are shown in red.*

	**1**	**2**	**3**	**4**	**5**	**6**	**7**	**8**	**Scale (µmole)**	**Standard Deviation**
**A**	5.07	5.61	5.54	5.17	5.26	4.97	5.17	4.85	30	0.26
**B**	4.93	4.95	4.13	4.28	4.48	4.45	4.95	4.65	40	0.32
**C**	4.57	3.10	4.43	3.9	4.16	4.4	4.67	4.53	60	0.51
**D**	2.81	4.24	2.69	2.02	2.39	1.12	2.41	3.78	90	0.98
**E**	2.12	1.72	1.04	1.86	1.72	1.57	2.11	1.48	120	0.35
**F**	0.94	0.91	0.88	0.86	0.88	0.89	0.88	0.95	150	0.03

3.3 Design of Experiments (DoE) campaign

Inside a nitrogen-filled purgebox, stock solutions of the reaction components were made as follows in acetonitrile: 4-bromoacetophenone (0.6 M), $[Ir(ppy)_2(dtbpy)]PF_6$ (3, 16.5 and 30 mM), nickel(II) chloride ethylene glycol dimethyl ether complex and ligand (2, 6 and 10 mM) and cyclopropyl bromide (0.3, 0.7 and 1 M). Low-point reactions were prepared with the least concentrated solution for each reagent or catalyst, center point reactions were prepared with the middle concentration, and high-point reactions were prepared with the most concentrated solutions. Sodium carbonate (30-90 µmol, 1.0-3.0 equiv.) was pre-weighed under nitrogen by a Chronect Quantos weighing robot into a 2 mL crimp-top HPLC vial. 4-bromoacetophenone solution (50 µL), and photocatalyst solution (10 µL), followed by tris(trimethylsilyl)silanol (30-90 µmol, 1.0-3.0 equiv.) were then added to the vial under nitrogen. A solution of nickel(II) chloride ethylene glycol dimethyl ether complex and 4,7-dimethoxy-1,10-phenanthroline (150 µL) was pre-mixed for 15 minutes before being added to the vial under nitrogen, followed by a solution of alkylating agent (90 µL). The vial was crimped and irradiated with an LED at 450 nm in the PHIL Pacer, with a light intensity of 300 mA and shaking using an orbital shaker at 150 rpm. The LED temperature typically

reached 35 °C. After 18 hours, 15 µL of the crude reaction mixture and 35 µL quench solution was added to 150 µL of acetonitrile and analyzed by LCMS (6 minute formic acid method). Reaction success was determined by assessing the peak area of each reaction component as a ratio to the internal standard (IS), *N,N*-dibenzylaniline.

Initial Scoping Experiment

The DoE experimental procedure was followed. The experiment included three "least forcing" wells (lowest mol % or equivalents for each factor), 6 center point wells and 3 "most forcing" wells (highest mol % or equivalents for each factor) (Table 15). These reactions were prepared and run simultaneously on a single plate within the same photoreactor, and were analyzed using the 2 minute formic acid LCMS method.

Table 15 Results of scoping DoE.

	Photocatalyst (mol%)	Nickel & ligand (mol%)	Silanol (equiv.)	Base (equiv.)	Alkylating agent (equiv.)	Nitrile product **4**/IS	Des-bromo starting material **4c**/IS	Starting material **5**/IS	Alkylation product **4a**/IS
Least Forcing	0.1	1	0.5	1	1	1.28	1.10	9.96	0.26
						1.41	1.18	9.13	0.23
						1.51	1.18	9.07	0.27
Centre Point	0.55	3	1.75	2	2	5.16	2.50	3.69	1.38
						5.16	2.52	4.19	1.56
						4.82	2.32	3.90	1.40
						4.73	2.28	2.92	1.75
						4.91	2.32	3.41	1.19
						5.19	2.41	2.57	1.20
Most Forcing	1	5	3	3	3	5.56	2.11	1.10	1.95
						5.03	1.99	2.20	1.78
						5.56	2.07	0.63	1.67

Full Factorial Design (Screening DoE)

The DoE experimental procedure was followed. This was conducted using a 2-level, 5-factor 32-run full-factorial design with 4 center points as outlined in Table 16. The reactions were prepared and run

simultaneously on a single plate within the same photoreactor. Statistical analysis was performed to evaluate the effect of the factors on the reaction component/internal standard ratio in Design Expert 13, using half normal plots to select the statistical model and ANOVA to assess statistically significant effects.

Table 16 Results of screening DoE.

Std	Run	Factor 1	Factor 2	Factor 3	Factor 4	Factor 5	Response 1	Response 2	Response 3	Response 4
		A:Ir	B:Ni	C: Silanol	D:Base	E: Alkylating agent	Product **4**/ Internal Standard	Desbromo **4c**/ Internal Standard	Starting Material **5**/ Internal Standard	Alkylated Product **4a**/ Internal Standard
		mol%	mol%	Equiv.	Equiv.	Equiv.				
13	1	0.1	1	3	3	1	2.11	1.78	10.05	0.00
5	2	0.1	1	3	1	1	1.40	1.48	10.62	0.00
22	3	1	1	3	1	3	6.17	2.25	0.21	0.84
4	4	1	5	0.5	1	1	4.54	3.19	5.23	0.33
30	5	1	1	3	3	3	6.80	2.16	0.22	1.31
31	6	0.1	5	3	3	3	1.99	1.07	13.34	0.00
25	7	0.1	1	0.5	3	3	0.71	0.76	13.24	0.00
20	8	1	5	0.5	1	3	4.69	2.10	7.88	0.37
14	9	1	1	3	3	1	3.59	2.90	0.05	0.73
33	10	0.55	3	1.75	2	2	6.04	2.62	1.67	1.24
19	11	0.1	5	0.5	1	3	0.66	0.54	14.02	0.00
24	12	1	5	3	1	3	5.94	2.40	0.92	1.02
29	13	0.1	1	3	3	3	1.32	0.84	12.32	0.00
10	14	1	1	0.5	3	1	5.93	3.43	1.11	0.46
15	15	0.1	5	3	3	1	1.99	1.57	9.61	0.16
23	16	0.1	5	3	1	3	1.40	0.85	12.23	0.00
34	17	0.55	3	1.75	2	2	6.33	2.96	1.31	2.06
36	18	0.55	3	1.75	2	2	6.52	2.95	2.25	1.38
21	19	0.1	1	3	1	3	1.17	0.75	12.78	0.00

3	20	0.1	5	0.5	1	1	0.87	0.87	13.30	0.00
11	21	0.1	5	0.5	3	1	1.34	1.01	11.85	0.00
1	22	0.1	1	0.5	1	1	0.43	0.57	13.97	0.00
6	23	1	1	3	1	1	3.89	3.20	0.00	0.53
16	24	1	5	3	3	1	4.82	3.22	0.32	1.96
35	25	0.55	3	1.75	2	2	6.34	2.80	1.55	1.08
7	26	0.1	5	3	1	1	1.51	1.46	11.44	0.00
12	27	1	5	0.5	3	1	4.66	2.90	5.16	0.55
32	28	1	5	3	3	3	6.09	2.09	0.18	2.46
18	29	1	1	0.5	1	3	5.94	2.55	4.41	0.45
28	30	1	5	0.5	3	3	6.17	2.34	6.38	1.28
26	31	1	1	0.5	3	3	6.70	2.39	4.18	0.34
9	32	0.1	1	0.5	3	1	2.04	2.00	14.13	0.00
2	33	1	1	0.5	1	1	5.76	3.77	0.92	0.56
17	34	0.1	1	0.5	1	3	1.09	0.85	13.30	0.00
8	35	1	5	3	1	1	5.27	3.64	0.25	0.96
27	36	0.1	5	0.5	3	3	1.40	0.96	15.18	0.00

Central composite design (Optimization DoE)

As significant curvature was identified in the screening DoE, the initial full-factorial design was augmented with axial points to identify the cause of the curvature, creating a face centered central composite design. The design is outlined in Table 17, which incorporates axial points for each of the 5 factors, as well as 6 center points. Additionally, as part of the robustness DoE (Table 19), a quarter fraction factorial design with 6 center points was included on the plate and this data was also incorporated into the analysis to increase the statistical power. The reactions were prepared and run simultaneously on a single plate within the same photoreactor. Statistical analysis was performed to evaluate the effect of the factors on the reaction component/internal standard ratio in Design Expert 13 and visualized in SAS V3.82. As the

data was generated in 3 stages across 3 plates, several approaches were taken to identify suitable statistical models for each response. In this section we present the combined model that was selected using a backwards selection approach, where the candidate list of terms included main effects, two factor interactions, three factor interactions and quadratic terms. Terms were included in the final model if $p<0.05$ or they were required to support marginality. For the analysis of the alkylated product response, the data was log transformed prior to analysis to stabilize the variance, and hence satisfy the parametric assumptions, using the formula:

$$\textit{transformed response} = \ln(\textit{response} + 0.1).$$

Note, the results from run 14 (center point) from the augmented design was deemed to be an outlier and was removed from the analysis as the product/internal standard value was significantly lower than for all other center points generated across the three plates.

Table 17 Axial points and center points and associated responses for augmented design.

Run	Factor 1	Factor 2	Factor 3	Factor 4	Factor 5	Response 1	Response 2	Response 3	Response 4
	A:Ir	B:Ni	C: Silanol	D:Base	E: Alkylating agent	Product **4**/ Internal Standard	Des-bromo **4c**/ Internal Standard	Starting Material **5**/ Internal Standard	Alkylated Product **4a**/ Internal Standard
	mol%	mol%	Equiv.	Equiv.	Equiv.				
1	0.55	5	1.75	2	2	1.33	1.03	10.14	0.10
2	0.55	3	1.75	1	2	5.69	2.50	2.04	0.38
3	0.1	3	1.75	2	2	1.13	0.74	12.90	0.00
4	0.55	3	1.75	2	2	6.39	2.53	1.60	0.54
5	0.55	1	1.75	2	2	6.83	2.56	0.65	0.69
6	0.55	3	1.75	2	2	6.11	2.38	2.26	0.51
7	0.55	3	1.75	2	2	6.20	2.31	1.10	0.60
8	0.55	3	0.5	2	2	4.26	1.86	6.11	0.23
9	1	3	1.75	2	2	5.67	2.26	0.13	0.98
10	0.55	3	1.75	2	3	6.27	2.20	2.96	0.53

11	0.55	3	1.75	3	2	5.73	2.22	0.23	0.75
12	0.55	3	1.75	2	1	5.50	3.15	0.52	0.53
13	0.55	3	1.75	2	2	6.45	2.60	1.28	0.50
**14**	**0.55**	**3**	**1.75**	**2**	**2**	**3.75**	**1.81**	**6.97**	**0.33**
15	0.55	3	3	2	2	5.84	2.32	0.32	0.58
16	0.55	3	1.75	2	2	6.02	2.32	1.49	0.51

From this analysis, the curvature was identified to be caused by non-linear effects of iridium photocatalyst and nickel catalyst stoichiometry, which can be seen from the Analysis of Variance (ANOVA) table (Table 18).

Table 18 ANOVA table for optimization DoE data. Factors with a p-value less than 0.05 are considered significant.

Source	DF	Mean Square	F-value	p-value
A-Ir	1	221.38	426.21	< 0.0001
B-Ni	1	3.94	7.59	0.0078
C-Silanol	1	0.4696	0.9041	0.3456
D-Base	1	1.73	3.34	0.0729
E-Alkylating agent	1	1.88	3.62	0.0620
AD	1	0.9264	1.78	0.1869
AE	1	5.41	10.42	0.0021
BC	1	2.85	5.48	0.0227
DE	1	0.314	0.6044	0.4400
A^2	1	13.44	25.88	< 0.0001
B^2	1	5.66	10.89	0.0017
ADE	1	4.57	8.8	0.0044
Residual	58	0.5194		

Figure 4 depicts the change in product/internal standard ratio when the stoichiometry of iridium, base and alkylating agent are varied.

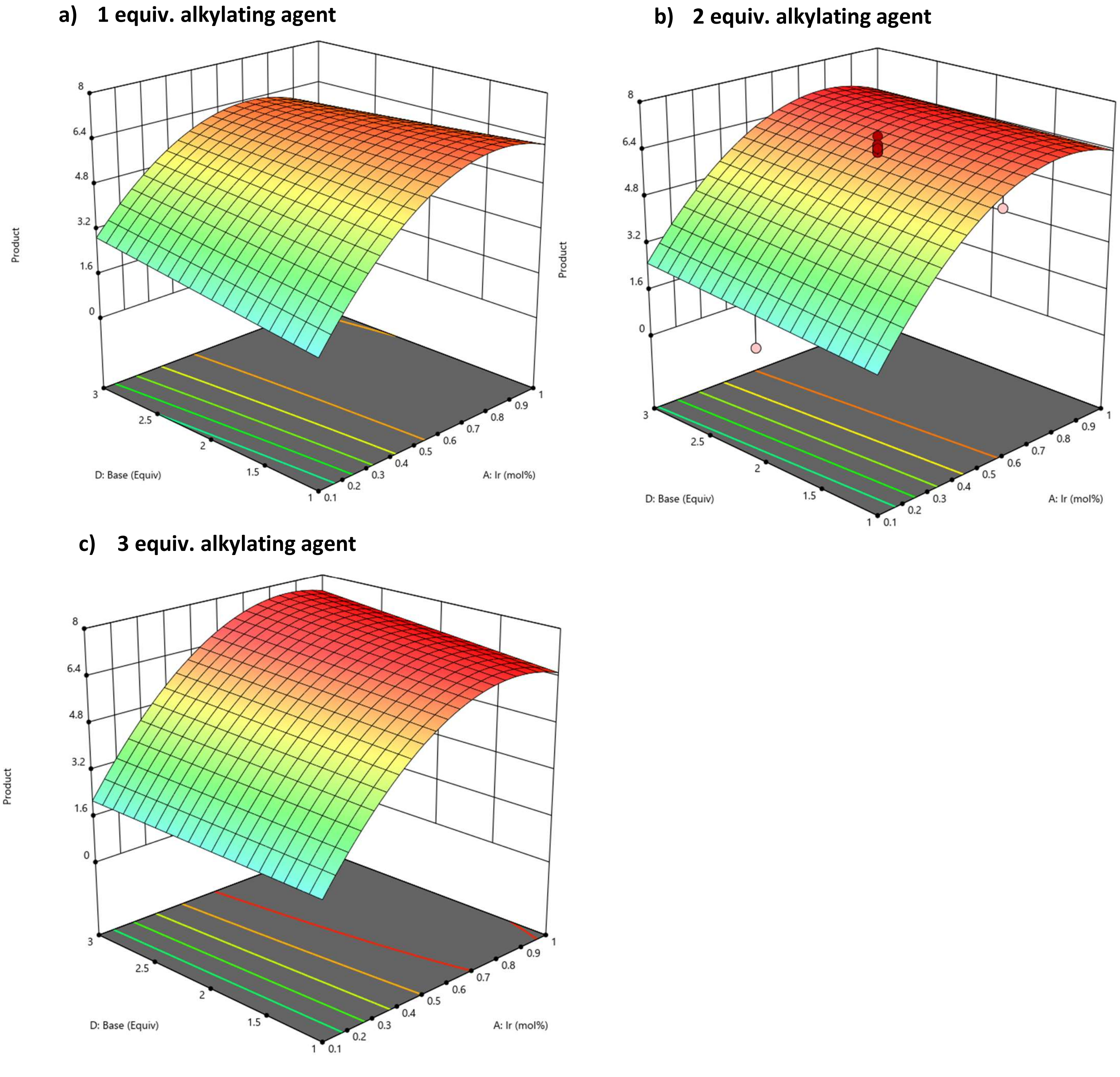

Figure 4 *The effect of iridium loading and base on the product/internal standard ratio at three levels of alkylating agent equivalents, with all other factors held at center point conditions. Center points and axial points are shown as circles.*

Although a potential interaction was noted, between nickel (B) and silanol (C) (Figure 5), the variation in product/internal standard values is relatively small, therefore this interaction may not be significant in a practical sense.

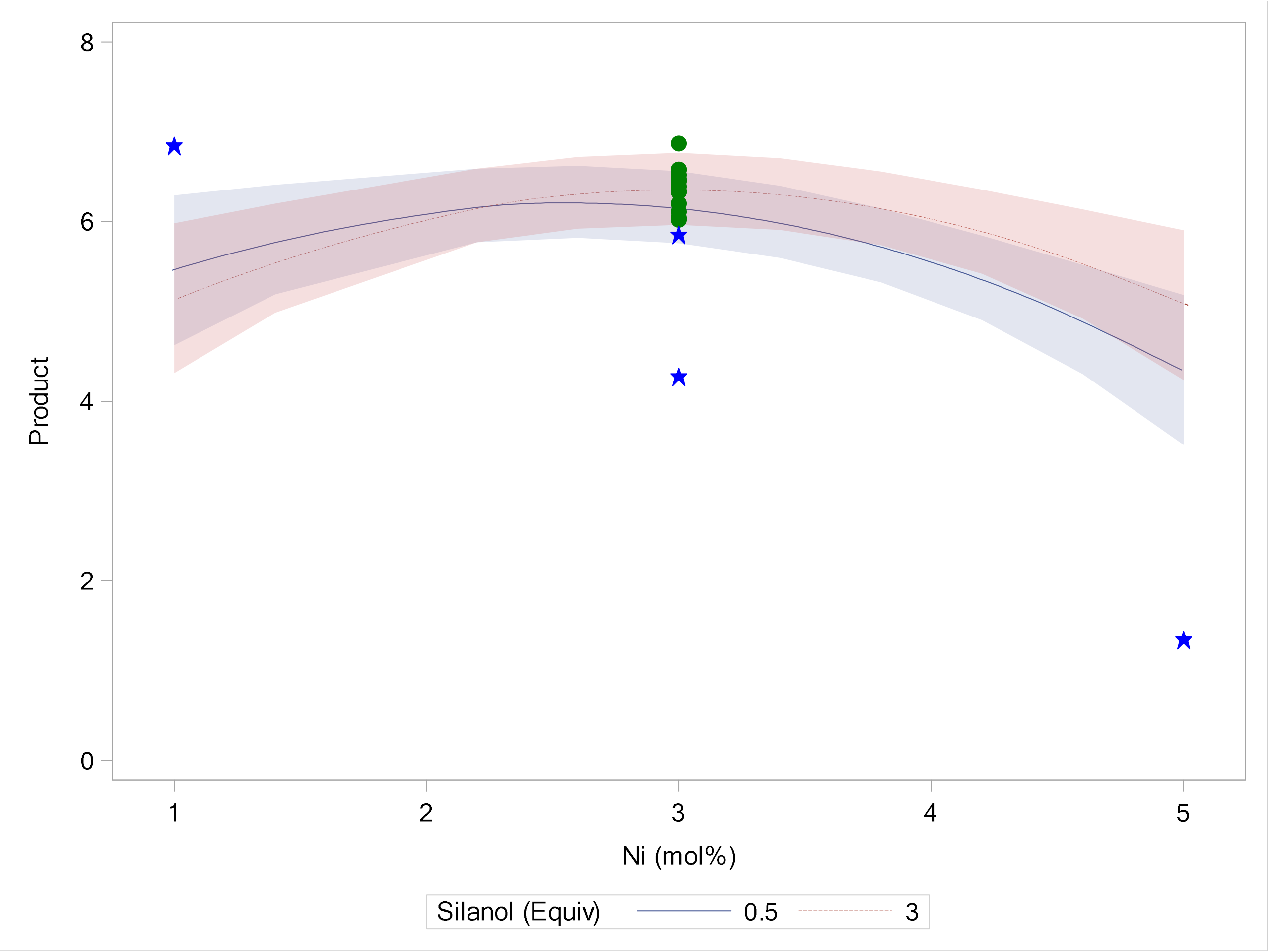

Figure 5 The predicted effect of increasing nickel concentration on the P/IS ratio, at low (blue line) and high (red line) equivalents of silanol,with all other factors held at center point conditions. Center points are shown as green circles and axial points are shown as blue stars.

The amount of remaining starting material, dehalogenated starting material and cyclopropyl alkylated product were monitored in these experiments. Increased loadings of iridium were found to accelerate dehalogenation, particularly at low levels of base and alkylating agent (Figure 6).

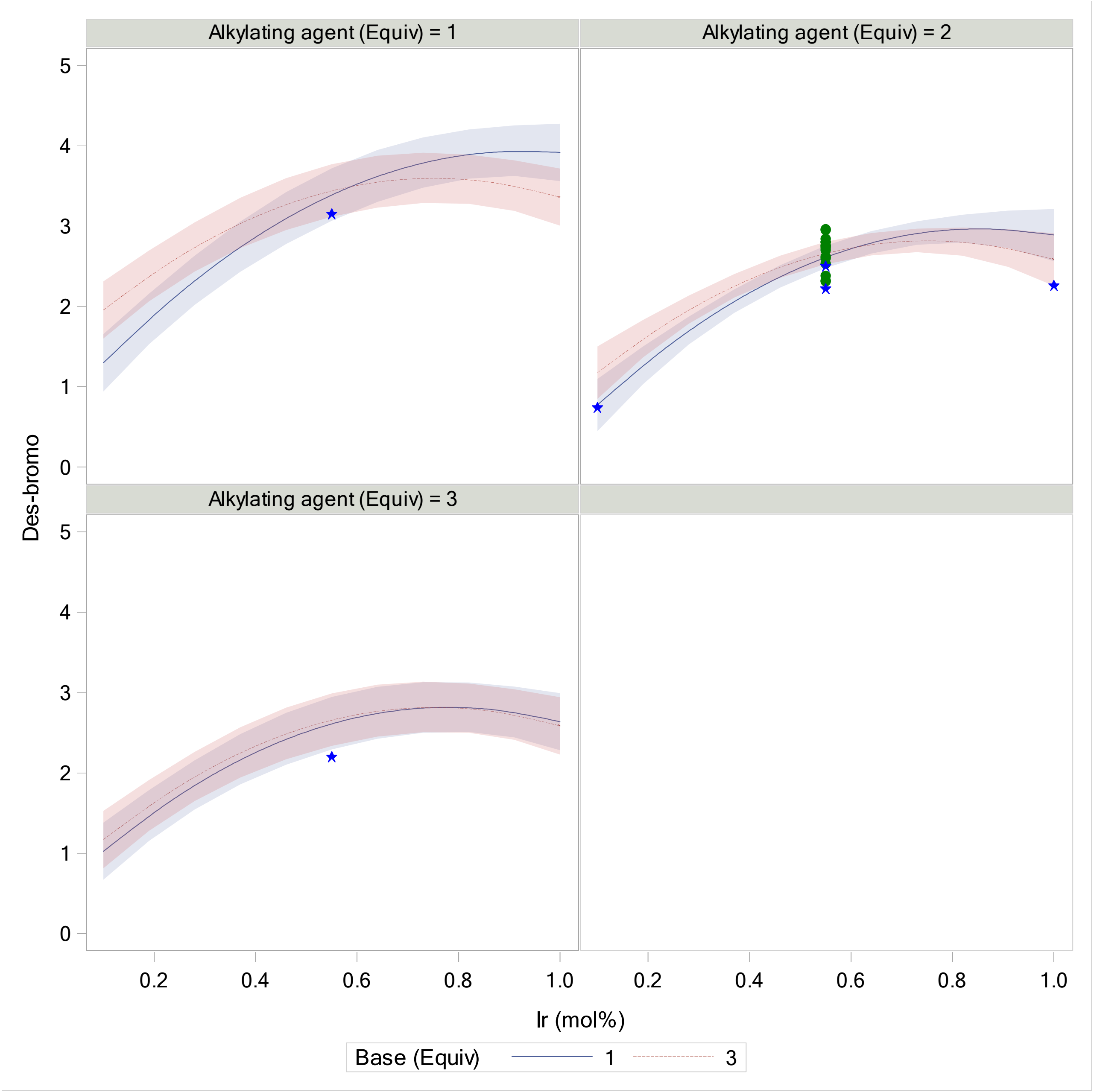

Figure 6 The effect of increasing iridium loading on the des-bromo/IS ratio, at low (blue line) and high (red line) equivalents of base at three levels of alkylating agent equivalents, with all other factors held at center point conditions. Center points are shown as green circles and axial points are shown as blue stars.

The formation of the cyclopropyl alkylated product (**4a**) was governed by three main trends (Figure 7). Its formation increased as the loading of iridium increased, up to approximately 0.8 mol %, and moderate levels of nickel aided alkylation, with low and high levels being detrimental (similar to the trend for desired product formation). Interestingly, at low levels of base, silanol equivalents have minimal impact on alkylation, however, at high levels of base, increasing silanol stoichiometry drastically increases the extent of alkylation (7c). This could also be connected to pH control of the reaction mixture.

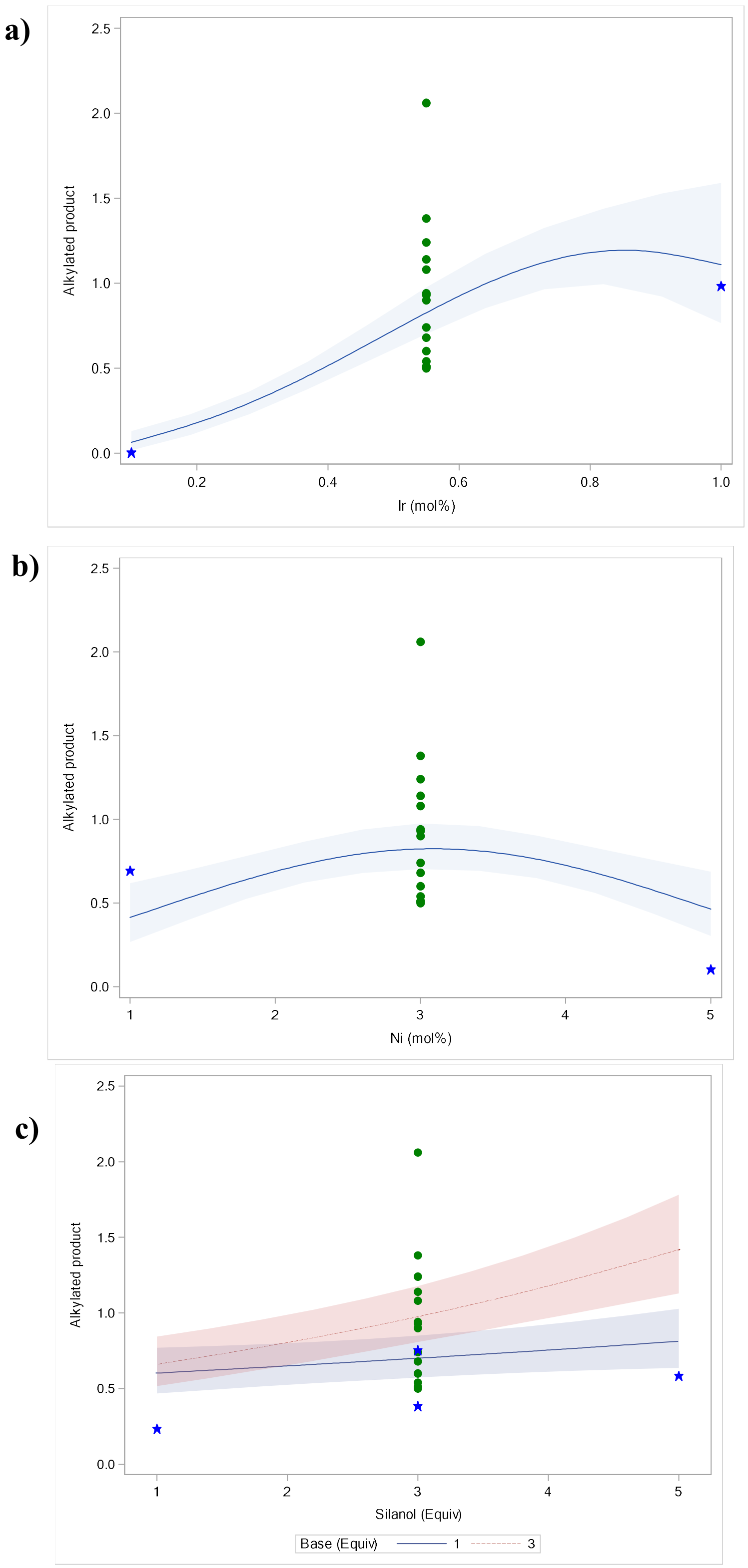

Figure 7 a) The effect of increasing iridium loading on the alkylated product/IS ratio. b) The effect of increasing nickel loading on the alkylated product/IS ratio. c) The effect of increasing equivalents of silanol on the alkylated product/IS ratio, at low (blue line) and high (red line) equivalents of base. All other factors were held at center point conditions. Center points are shown as green circles and axial points are shown as blue stars.

High iridium loadings increase the consumption of starting material, particularly with a larger excess of silanol, which is consistent with the fact that increasing the stoichiometry of iridium and silanol increase product and side-product formation (Figure 8). Higher nickel loading also increases consumption of starting material (**5**) in some cases, which mirrors the favourable stoichiometry for product formation and dehalogenation.

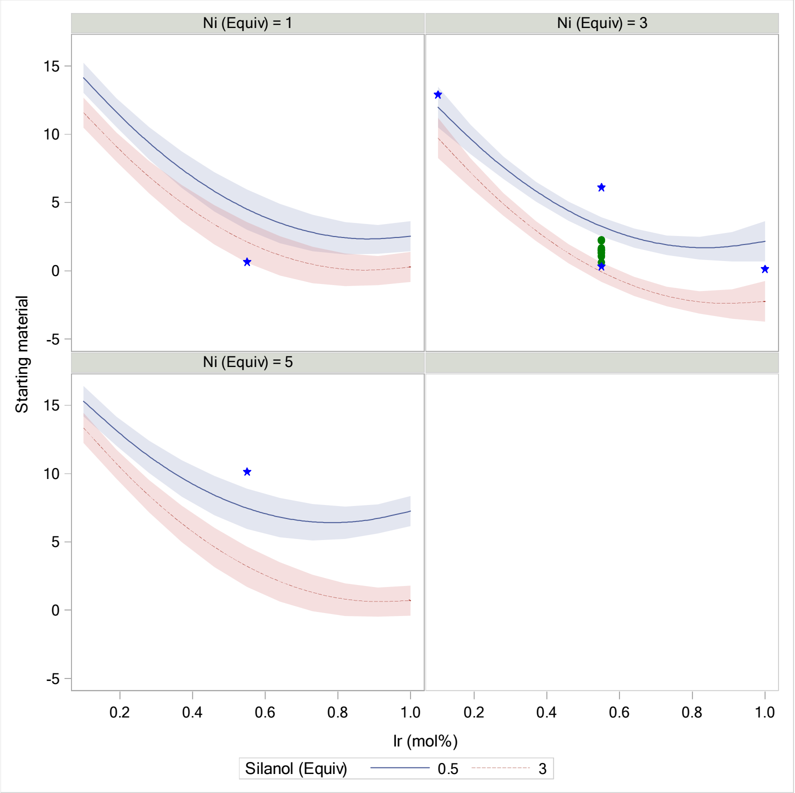

Figure 8 The effect of increasing iridium loading on the starting material/IS ratio, at low (blue line) and high (red line) equivalents of silanol at three levels of nickel, with all other factors held at center point conditions. Center points are shown as green circles and axial points are shown as blue stars.

Robustness Assessment

The robustness design is outlined in Table 19, which incorporates six center points, as well as the robustness experiments (each robustness run performed in duplicate) and the quarter fraction factorial runs used

in the analysis above. The reactions were prepared and conducted simultaneously on a single plate within the same photoreactor. The robustness data points were plotted as a scatterplot using Statistica V13.

Table 19 Robustness design of experiments and associated responses.

Run	Factor 1	Factor 2	Factor 3	Factor 4	Factor 5	Response 1	Response 2	Response 3	Response 4
	A: Ir	B: Ni	C: Si-lanol	D: Base	E: Alkylat-ing agent	Product **4**/ Internal Standard	Des-bromo **4c**/ Internal Standard	Starting Material **5**/ Internal Standard	Alkylated Product **4a**/ Internal Standard
	mol%	mol%	equiv.	equiv.	equiv.				
1	0.100	1.0	0.5	3.0	3.0	1.38	0.82	14.78	0.00
2	0.100	1.0	0.5	3.0	3.0	1.38	0.85	14.83	0.00
3	1.000	1.0	0.5	1.0	1.0	6.73	4.42	0.25	0.65
4	1.000	1.0	0.5	1.0	1.0	6.31	3.90	1.36	0.53
5	0.100	5.0	0.5	1.0	3.0	0.11	0.19	17.99	0.00
6	0.100	5.0	0.5	1.0	3.0	0.12	0.20	17.12	0.00
7	1.000	5.0	0.5	3.0	1.0	3.15	2.56	7.92	0.47
8	1.000	5.0	0.5	3.0	1.0	3.95	2.89	6.16	0.54
9	0.100	1.0	3.0	3.0	1.0	2.04	1.73	10.36	0.22
10	0.100	1.0	3.0	3.0	1.0	2.74	2.04	10.57	0.13
11	1.000	1.0	3.0	1.0	3.0	5.11	2.27	0.29	0.66
12	1.000	1.0	3.0	1.0	3.0	5.79	2.26	1.50	0.63
13	0.100	5.0	3.0	1.0	1.0	0.42	0.73	13.81	0.00
14	0.100	5.0	3.0	1.0	1.0	0.72	0.93	14.99	0.08
15	1.000	5.0	3.0	3.0	3.0	6.70	2.23	0.20	1.40
16	1.000	5.0	3.0	3.0	3.0	6.31	2.33	1.08	1.50
17	0.550	3.0	1.75	2.0	2.0	6.33	2.84	2.18	0.94
18	0.550	3.0	1. 75	2.0	2.0	6.58	2.70	1.03	0.74
19	0.550	3.0	1. 75	2.0	2.0	6.87	2.80	0.61	1.14
20	0.550	3.0	1. 75	2.0	2.0	6.47	2.75	1.48	0.93
21	0.550	3.0	1. 75	2.0	2.0	6.58	2.76	1.50	0.68
22	0.550	3.0	1. 75	2.0	2.0	6.46	2.73	1.34	0.90

23	0.505	2.8	1.6	2.1	2.1	6.38	2.72	4.40	0.87
24	0.505	2.8	1.6	2.1	2.1	5.83	2.51	3.07	0.75
25	0.595	2.8	1.6	1.9	1.9	7.59	3.24	0.67	0.85
26	0.595	2.8	1.6	1.9	1.9	6.97	2.98	1.46	0.72
27	0.505	3.2	1.6	1.9	2.1	6.09	2.62	3.78	0.68
28	0.505	3.2	1.6	1.9	2.1	5.77	2.55	3.96	0.59
29	0.595	3.2	1.6	2.1	1.9	6.25	2.78	2.06	0.77
30	0.595	3.2	1.6	2.1	1.9	5.98	2.68	2.22	0.82
31	0.505	2.8	1.9	2.1	1.9	5.42	2.53	3.82	0.46
32	0.505	2.8	1.9	2.1	1.9	6.21	2.73	1.67	0.76
33	0.595	2.8	1.9	1.9	2.1	6.48	2.61	1.36	1.06
34	0.595	2.8	1.9	1.9	2.1	6.98	2.77	1.35	0.82
35	0.505	3.2	1.9	1.9	1.9	5.47	2.59	4.24	0.60
36	0.505	3.2	1.9	1.9	1.9	5.56	2.55	3.30	0.54
37	0.595	3.2	1.9	2.1	2.1	5.64	2.36	2.24	0.68
38	0.595	3.2	1.9	2.1	2.1	6.48	2.82	2.04	0.60

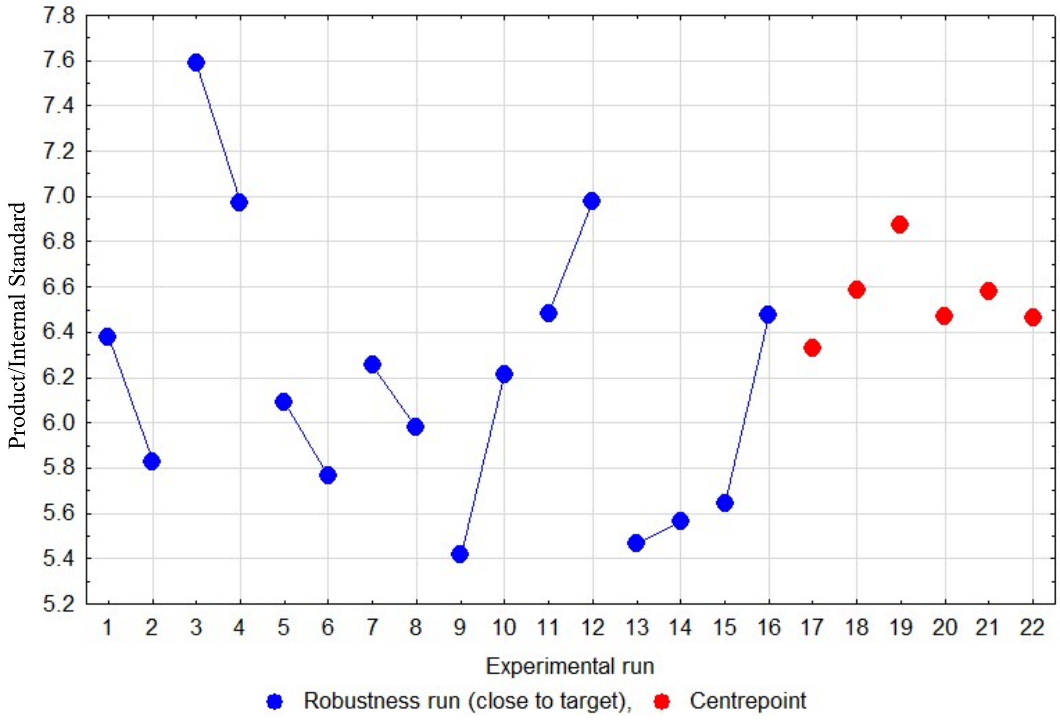

Figure 9 Variation in product/internal standard relative across robustness reactions (blue), relative to center point reactions (red). Dots joined together depict reactions with identical reaction conditions, where variation is due to well-to-well variability, or plate-to-plate variability, rather than deliberate variation in the amount of reagent dosed.

3.4 Screening of Continuous Variables

Concentration & Silanol Equivalents Screen

Inside a nitrogen-filled purgebox, sodium carbonate was weighed under nitrogen by a Chronect Quantos weighing robot into 36 separate 2 mL crimp-top HPLC vials, with the quantities specified in Table 21 (4 vials per quantity). An iridium stock solution (Ir solution) was prepared by dissolving $[Ir(ppy)_2(dtbpy)]PF_6$ (9.1 mg) in acetonitrile (374.9 μL). A nickel/ligand solution was prepared by dissolving $NiCl_2$.glyme (7.7 mg) and 4,7-dimethoxy-1,10-phenanthroline (8.6 mg) in acetonitrile (5.7 mL). A bromocyclopropane solution (AlkBr solution) was prepared by dissolving bromocyclopropane (422.1 mg, 279.5 μL) in acetonitrile (3.2 mL). A bromoacetophenone solution (SM solution) was prepared by dissolving 4-bromoacetophenone (229.2 mg) in acetonitrile (1.7 mL). These stock solutions were used to prepare reaction solutions at 0.1, 0.5, 1 and 3 equivalents of silanol (Table 20).

Table 20 Preparation of reaction mixtures with silanol equivalents from 0.1 to 3.

<table>
<tr><th>Equivalents of silanol</th><th>SM solution (μL)</th><th>Ir solution (μL)</th><th>Silanol (μL)</th><th>Ni solution (μL)</th><th>AlkBr solution (μL)</th></tr>
<tr><td>0.1</td><td rowspan="4">400</td><td rowspan="4">80</td><td>7.8</td><td rowspan="4">1200</td><td rowspan="4">720</td></tr>
<tr><td>0.5</td><td>38.9</td></tr>
<tr><td>1</td><td>77.8</td></tr>
<tr><td>3</td><td>233.5</td></tr>
</table>

These solutions were dosed directly onto the pre-weighed sodium carbonate and acetonitrile was added to each vial to make all reactions up to a total volume of 0.3 mL (Table 21). The starting material solution was added first, followed by the iridium solution, the silanol, the nickel solution and the alkylating agent

solution. The vials were crimped and each vial was irradiated with an LED at 450 nm in the PHIL Pacer, with a light intensity of 300 mA and shaking using an orbital shaker at 150 rpm. The LED temperature typically reached 35 °C. After 18 hours, the crude reaction mixture was added to 35 μL quench solution and 150 μL of acetonitrile (see Table 22 for quantities) and analyzed by LCMS (2 minute formic acid method). Reaction success was determined by assessing the peak area of each reaction component as a ratio to the internal standard, *N,N*-dibenzylaniline (Table 23). These results showed that concentration does not have a significant impact on reaction outcome, and the equivalents of supersilanol could be reduced to 1, which makes the reaction more economical and improves atom economy.

Table 21 Preparation of reactions at concentrations ranging from 0.05 to 0.1M, made using the solutions from Table 20.

Concentration (M)	μmoles of limiting reagent, **5**	Sodium Carbonate (mg)	Volume of reac-tion solution (μL)	MeCN (μL)
0.050	15	4.8	150.0	150.0
0.060	18	5.7	180.0	120.0
0.070	21	6.7	210.0	90.0
0.080	24	7.6	240.0	60.0
0.090	27	8.6	270.0	30.0
0.093	28.0	8.9	280.0	20.0
0.095	28.5	9.0	285.0	15.0
0.097	29.0	9.2	290.0	10.0
0.100	30.0	9.5	300.0	0.0

Table 22 Preparation of LCMS samples.

Concentration (M)	0.05	0.06	0.07	0.08	0.09	0.093	0.095	0.097	0.1
Reaction mixture added to LCMS sample (μL)	15.0	12.5	10.7	9.4	8.3	8.1	7.9	7.7	7.5

*Table 23 Ratio of product **4** to internal standard after reacting at concentrations ranging from 0.05M to 0.1M, and with quantities of supersilanol ranging from 0.1 to 3 equivalents.*

Silanol (equiv.)	Concentration (M)								
	**0.050**	**0.060**	**0.070**	**0.080**	**0.090**	**0.093**	**0.095**	**0.097**	**0.100**
**0.1**	0.06	0.06	0.11	0.25	0.30	0.34	0.42	0.38	0.39
**0.5**	1.80	1.94	2.11	2.50	2.37	2.55	2.72	2.22	2.44
**1**	3.25	3.33	3.37	3.33	3.63	3.42	3.68	3.23	3.21
**3**	3.73	3.55	3.59	3.65	3.32	3.65	3.69	3.69	4.44

4. Preparative Reactions

4.1 General Procedure for Reactions in Lucent360

Inside a nitrogen-filled purgebox, sodium carbonate (95.4 mg, 900 μmol, 3.0 equiv.) was weighed under nitrogen into a 4 mL screw-top borosilicate vial with a PTFE septum. Aryl halide (300 μmol, 1.0 equiv.) in acetonitrile (1 mL), and $[Ir(dtbpy)(ppy)_2]PF_6$ (2.3 mg, 2.55 μmol, 0.0085 equiv.) in acetonitrile (0.2 mL), followed by tris(trimethylsilyl)silanol (102 μL, 330 μmol, 1.1 equiv.) were then added to the vial. A solution of $NiCl_2$.glyme (2.0 mg, 9.00 μmol, 0.03 equiv.) and 4,7-dimethoxy-1,10-phenanthroline (2.2 mg, 9.00 μmol, 0.03 equiv.) in acetonitrile (1 mL) was pre-mixed for 15 mins, then added to the vial, followed by cyclopropyl bromide (72 μL, 900 μmol, 3.0 equiv.). The vial was sealed, removed from the purgebox and irradiated with 450 nm LEDs using the Lucent360 reactor. A Julabo F25 refrigerated/heating circulator containing Slytherm™, set to 40 °C, was used to maintain temperature of the water bath. Stirring was set to 1000 rpm and all LED panels were set to 100% intensity. After 22-88 hours, 3 μL of the crude reaction mixture and 50 μL neutral quench solution was diluted into 100 μL of acetonitrile and analyzed by LCMS. Conversion and P/IS values were recorded for each substrate. Each reaction was prepared in duplicate; one reaction mixture was used to determine NMR yield, and one was purified to give an isolated yield.

4.2 Determination of NMR Yield

General procedure for reactions in Lucent360 was followed. Inside a nitrogen-filled purgebox, maleic acid was dissolved in DMSO-d_6 to prepare a 3.33 mg/mL internal standard (IS) solution. NMR samples were prepared by diluting 300 µL reaction mixture supernatant with 300 µL IS solution. Samples were analyzed by ^{1}H NMR using a "wet" solvent suppression method to suppress the acetonitrile signal. NMR yields were calculated using a published method. [7] The theoretical amount of product in the 300 µL aliquot of reaction mixture was calculated by taking into account the total reaction mixture volume, including both solid and liquid reagents and reactants. For example, the total reaction mixture volume for substrate **2** was 3.25 mL (3 mL solvent plus 0.25 mL reagents and reactants – calculated within Katalyst software[1]). Therefore, on a 0.3 mmol scale, within a 300 µL aliquot, 0.028 mmol of product could theoretically be present.

4.3 Determination of Purity and Isolated Yield

Purity of purified compounds was determined by LCMS analysis. The yields stated have been adjusted to account for purity. NMR analysis was used to evaluate alkyl impurities and remaining solvent. If these were present, the product was re-purified or dried further as appropriate.

4.4 Substrate Scope

Synthesis of 2-(4-acetylphenyl)acetonitrile, **4**

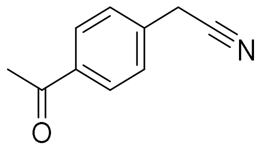

Before optimisation

Inside a nitrogen-filled purgebox, sodium carbonate (63.6 mg, 600 μmol, 2.0 equiv.) was weighed into a 5 mL microwave vial. 4-bromoacetophenone (300 μmol, 1.0 equiv.) in acetonitrile (1 mL), and (Ir[dF(CF_3)ppy]$_2$(dtbpy))PF_6 (7 mg, 6 μmol, 0.02 equiv.) in acetonitrile (1 mL), followed by tris(trimethylsilyl)silanol (139 μL, 450 μmol, 1.5 equiv.) were then added to the vial. A solution of nickel(II) chloride ethylene glycol dimethyl ether complex (3.3 mg, 15 μmol, 0.05 equiv.) and 4,4'-di-*tert*-butyl-2,2'-dipyridyl (4.0 mg, 15 μmol, 0.05 equiv.) in acetonitrile (1 mL) was pre-mixed for 10 mins, then added to the vial, followed by cyclopropyl bromide (48 μL, 600 μmol, 2.0 equiv.). The vial was crimped, removed from the purgebox and irradiated with an LED at 450 nm in the PHIL Pacer, with a light intensity of 300 mA and stirring using a hotplate stirrer on full speed. The LED temperature typically reached 35 °C. After 16.5 hours, 15 μL of the crude reaction mixture was diluted into 0.2 mL of acetonitrile and analyzed by LCMS. Solvent was evaporated under nitrogen at 40 °C to give crude material. Crude material was purified by column chromatography (10-40% EtOAc in heptane, Biotage SNAP Silica column) to afford 2-(4-acetylphenyl)acetonitrile as a pale orange oil (4 mg, 8%).

Following optimisation of discrete variables

Inside a nitrogen-filled purgebox, sodium carbonate (63.6 mg, 600 μmol, 2.0 equiv.) was weighed into a 5 mL microwave vial. 4-bromoacetophenone (300 μmol, 1.0 equiv.) in acetonitrile (1 mL), and [Ir(dtbpy)(ppy)$_2$]PF_6 (2.7 mg, 3 μmol, 0.01 equiv.) in acetonitrile (1 mL), followed by tris(trimethylsilyl)silanol (139 μL, 450 μmol, 1.5 equiv.) were then added to the vial. A solution of nickel(II) chloride ethylene glycol dimethyl ether complex (3.3 mg, 15 μmol, 0.05 equiv.) and 4,7-dimethoxy-1,10-phenanthroline (3.6 mg, 15 μmol, 0.05 equiv.) in acetonitrile (1 mL) was pre-mixed for 15 mins, then added to the vial, followed by cyclopropyl bromide (48 μL, 600 μmol, 2.0 equiv.). The vial was crimped, removed from the purgebox and irradiated with an LED at 450 nm in the PHIL Pacer, with a light intensity of 300 mA and stirring using a hotplate stirrer on full speed. The LED temperature typically reached 35 °C. After 18 hours, 3 μL of the crude reaction mixture was diluted into 100 μL of acetonitrile and 50 μL of quench

solution analyzed by LCMS. The reaction mixture was diluted in DCM, adsorbed onto Florisil and purified by column chromatography (10-40% EtOAc in cyclohexane over 50 CV, 12 g RediSep Silica column, solid loaded). 1% EtOAc (5 CV) was flowed through the column prior to the gradient starting, in order to remove silanol-related species. Fractions containing product were combined and solvent removed under reduced pressure at 30 °C to give product as a pale yellow powder (24.7 mg, 50%).

Following DoE optimisation

Inside a nitrogen-filled purgebox, sodium carbonate (95.4 mg, 900 µmol, 3.0 equiv.) was weighed into a 5 mL microwave vial. 4-bromoacetophenone (300 µmol, 1.0 equiv.) in acetonitrile (1 mL), and $[Ir(dtbpy)(ppy)_2]PF_6$ (2.3 mg, 2.55 µmol, 0.0085 equiv.) in acetonitrile (1 mL), followed by tris(trimethylsilyl)silanol (277 µL, 900 µmol, 3.0 equiv.) were then added to the vial. A solution of nickel(II) chloride ethylene glycol dimethyl ether complex (2.0 mg, 9.0 µmol, 0.03 equiv.) and 4,7-dimethoxy-1,10-phenanthroline (2.2 mg, 9.0 µmol, 0.03 equiv.) in acetonitrile (1 mL) was pre-mixed for 15 mins, then added to the vial, followed by cyclopropyl bromide (72 µL, 900 µmol, 3.0 equiv.). The vial was crimped, removed from the purgebox and irradiated with an LED at 450 nm in the PHIL Pacer, with a light intensity of 300 mA and stirring using a hotplate stirrer on full speed. The LED temperature typically reached 35 °C. After 18 hours, 3 µL of the crude reaction mixture was diluted into 100 µL of acetonitrile and 50 µL of quench solution analyzed by LCMS. The reaction mixture was diluted in DCM, adsorbed onto Florisil and purified by column chromatography (10-40% EtOAc in cyclohexane over 50 CV, 12 g RediSep Silica column, solid loaded). 1% EtOAc (5 CV) was flowed through the column prior to the gradient starting, in order to remove silanol-related species. Fractions containing product were combined and solvent removed under reduced pressure at 30 °C to give product as a pale yellow powder (24.2 mg, 49%).

Fully optimized conditions – under air

Inside a nitrogen-filled purgebox, sodium carbonate (95.4 mg, 900 μmol, 3.0 equiv.) was weighed into a 5 mL microwave vial. 4-bromoacetophenone (300 μmol, 1.0 equiv.) in acetonitrile (1 mL), and [Ir(dtbpy)(ppy)$_2$]PF$_6$ (2.3 mg, 2.55 μmol, 0.0085 equiv.) in acetonitrile (1 mL), followed by tris(trimethylsilyl)silanol (102 μL, 330 μmol, 1.1 equiv.) were then added to the vial. A solution of nickel(II) chloride ethylene glycol dimethyl ether complex (2.0 mg, 9.0 μmol, 0.03 equiv.) and 4,7-dimethoxy-1,10-phenanthroline (2.2 mg, 9.0 μmol, 0.03 equiv.) in acetonitrile (1 mL) was pre-mixed for 15 mins, then added to the vial, followed by cyclopropyl bromide (72 μL, 900 μmol, 3.0 equiv.). The vial was removed from the purgebox and the contents were transferred to another vial under air, to ensure significant exposure to oxygen. The vial was irradiated with an LED at 450 nm in the PHIL Pacer, with a light intensity of 300 mA and stirring using a hotplate stirrer on full speed. The LED temperature typically reached 35 °C. After 18 hours, 3 μL of the crude reaction mixture was diluted into 100 μL of acetonitrile and analyzed by LCMS. The reaction mixture was diluted in DCM, adsorbed onto Florisil and purified by column chromatography (10-40% EtOAc in cyclohexane over 40 CV, 12 g RediSep Silica column, solid loaded). 1% EtOAc (5 CV) was flowed through the column prior to the gradient starting, in order to remove silanol-related species. Fractions containing product were combined and solvent removed under reduced pressure at 30 °C to give product as a pale yellow solid (24.3 mg, 50%).

Fully optimized conditions

General procedure for reactions in Lucent360 was followed, irradiating for 22 hours. The reaction mixture was diluted in DCM, adsorbed onto Florisil® and purified by column chromatography (10-40% EtOAc in cyclohexane over 50 CV, 12 g RediSep Silica column). 1% EtOAc (10 CV) was flowed through the column prior to the gradient starting, in order to remove silanol-related species. Fractions containing product were combined and solvent removed under reduced pressure at 30 °C to give product as a pale yellow solid (25.8 mg, 50%).

Preparation of the compound was attempted from the corresponding aryl triflate, using the same general procedure, irradiating for 22 hours. Trace amounts of product was formed in the reaction, therefore purification was not attempted (0.0 mg, 0%).

This compound was prepared from the corresponding aryl iodide, using the same general procedure (irradiating for 44 hours) and purification process described above, to give product as a yellow solid (2.1 mg, 4%).

This compound was also prepared from the corresponding aryl chloride, using the same general procedure (irradiating for 88 hours) and purification process described above, to give product as a white powder (14.4 mg, 30%). LCMS (2 minute high pH method): t_R = 0.74 min, $[M-H]^-$ 158, (93% purity). ^{1}H NMR (400 MHz, $CDCl_3$) δ ppm 7.98 (d, J = 8.5 Hz, 2H), 7.45 (d, J = 8.5 Hz, 2H), 3.82 (s, 2H), 2.61 (s, 3H). ^{13}C NMR (101 MHz, $CDCl_3$) δ ppm 197.2, 137.0, 135.0, 129.1, 128.2, 117.0, 26.6, 23.7. Characterisation data is consistent with literature reports. [8] NMR yield was calculated to be 57% when prepared from the bromide precursor, 4.2% from the iodide, 31% from the chloride, and 0.5% from the triflate.

Synthesis of 2-(1-oxo-1,3-dihydroisobenzofuran-5-yl)acetonitrile, **6**

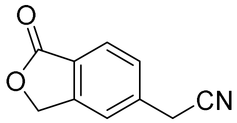

General procedure for reactions in Lucent360 was followed, irradiating for 22 hours. The reaction mixture was diluted in DCM, adsorbed onto Florisil® and purified by column chromatography (10-50% EtOAc in cyclohexane over 60 CV, 12 g RediSep Silica column). Fractions containing product were combined and solvent removed under reduced pressure at 30 °C to give a white solid (20.5 mg, 39%). LCMS (2

minute high pH method): t_R = 0.63 mins $[M-H]^-$ 172, (98% purity). ^1H NMR (400 MHz, $CDCl_3$) δ ppm 7.93 (d, *J* = 7.9 Hz, 1H), 7.54 – 7.56 (m, 1H), 7.49 - 7.53 (m, 1H), 5.34 (s, 2H), 3.93 (s, 2H). ^{13}C NMR (101 MHz, $CDCl_3$) δ ppm 170.1, 147.7, 136.6, 129.1, 126.5, 125.9, 121.8, 116.8, 69.4, 24.0. $\tilde{\nu}_{max}$ ($CDCl_3$ solution) = 3063, 2950, 2914, 2252, 1752, 1621, 1410, 1052, 1001, 767, 676. HRMS (10 minute formic acid): ($C_{10}H_6NO_2$) $[M-H^-]$ requires 172.0404, found $[M-H^-]$ 172.0403 (error -0.58 ppm). NMR yield was calculated to be 20%.

Synthesis of 2-(3-(trifluoromethyl)phenyl)acetonitrile, **7**

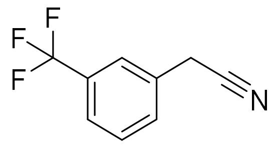

General procedure for reactions in Lucent360 was followed, irradiating for 22 hours. The reaction mixture was diluted in DCM, adsorbed onto Florisil® and purified by column chromatography (0-50% EtOAc in cyclohexane over 50 CV, 12 g RediSep Silica column). Fractions containing product were combined and solvent removed under reduced pressure at 30 °C to give product as a clear, colourless liquid (20.1 mg, 34%). LCMS (2 minute high pH method): t_R = 1.02 min, $[M-H^-]$ 184, (95% purity). ^1H NMR (400 MHz, $CDCl_3$) δ 7.63 - 7.51 (m, 4H), 3.82 (s, 2H). Traces of grease can be seen in spectrum. ^{13}C NMR (101 MHz, $CDCl_3$) δ 131.9, 131.3, 131.0, 129.8, 125.04 - 125.17 (m), 124.8 (q, *J* = 3.1 Hz), 122.3, 116.9, 23.5. ^{19}F NMR (376 MHz, $CDCl_3$) δ -62.85. Characterisation data is consistent with literature reports. [8] NMR yield was calculated to be 38%.

Synthesis of 2-(4-(*tert*-butoxy)phenyl)acetonitrile, **8**

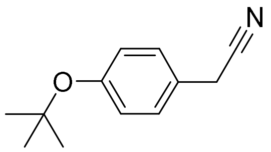

General procedure for reactions in Lucent360 was followed, irradiating for 88 hours. The reaction mixture was diluted in DCM, adsorbed onto Florisil® and purified by column chromatography (1-10% EtOAc in cyclohexane over 60 CV, 12 g RediSep Silica column, solid loaded). Fractions containing product were combined and solvent removed under reduced pressure at 30 °C to give crude product. Crude product was purified by reverse-phase column chromatography (30-85% acetonitrile in 10 mM ammonium bicarbonate in water (adjusted to pH 10 with ammonia solution) over 10 mins at 40 mL/min, Waters XSelect CSH Prep C18 5 µm, 30 x 100 mm). Fractions containing product were combined and acetonitrile was removed under reduced pressure at 30 °C. Product was extracted into DCM and solvent removed under reduced pressure at 30 °C to give product as a yellow oil (22.6 mg, 40%). LCMS (2 minute high pH method): t_R = 1.07 min, $[M+H]^+$ 190, (100% purity). ^1H NMR (400 MHz, $CDCl_3$) δ ppm 7.24 (d, J = 8.6 Hz, 2H), 7.01 (d, J = 8.6 Hz, 2H), 3.72 (s, 2H), 1.37 (s, 9H). ^{13}C NMR (101 MHz, $CDCl_3$) δ ppm 155.3, 128.5, 124.6, 124.5, 118.1, 78.8, 28.8, 23.0. $\tilde{\nu}_{max}$ ($CDCl_3$ solution) = 2978, 2933, 2250, 1610, 1507, 1238, 1160, 894 cm^{-1}. HRMS (10 minute formic acid): ($C_{12}H_{16}NO$) $[M+H^+]$ requires 190.1226, found $[M+H^+]$ 190.1224 (error -1.05 ppm). NMR yield was calculated to be 50%.

Synthesis of 2-(4-((1-hydroxycyclopropyl)methyl)phenyl)acetonitrile, **9**

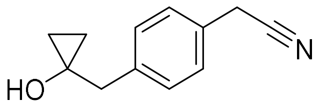

General procedure for reactions in Lucent360 was followed, irradiating for 66 hours. The reaction mixture was diluted in DCM, adsorbed onto Florisil® and purified by column chromatography (5-25% EtOAc in cyclohexane over 50 CV, 12 g RediSep Silica column, solid loaded). 1% EtOAc (10 CV) was flowed through the column prior to the gradient starting, in order to remove silanol-related species. Fractions containing product were combined and solvent removed under reduced pressure at 30 °C to give crude product. Crude product was purified by reverse-phase column chromatography (15-55% acetonitrile in 10 mM ammonium bicarbonate in water (adjusted to pH 10 with ammonia solution) over 10 mins at 40 mL/min, Waters XSelect CSH Prep C18 5 μm, 30 x 100 mm). Fractions containing product were combined and acetonitrile was removed under reduced pressure at 30 °C. Product was extracted into DCM and solvent removed under reduced pressure at 30 °C to give product as off-white crystals (13.0 mg, 23%). LCMS (2 minute high pH method): t_R = 0.84 min, no mass ion observed, (99% purity). ^{1}H NMR (400 MHz, $CDCl_3$) δ ppm 7.26 - 7.34 (m, 4H), 3.73 (s, 2H), 2.88 (s, 2H), 1.84 - 1.91 (m, 1H), 0.76 - 0.90 (m, 2H), 0.58 - 0.70 (m, 2H). ^{13}C NMR (101 MHz, $CDCl_3$) δ ppm 138.8, 130.2, 128.2, 128.1, 117.9, 56.0, 43.7, 23.3, 13.4. $\tilde{\nu}_{max}$ ($CDCl_3$ solution) = 3306, 3215, 2910, 2247, 1515, 1436, 1288, 1014, 740 cm^{-1}. HRMS (10 minute formic acid): ($C_{12}H_{14}NO$) [M+H$^+$] requires 188.1070, found [M+H$^+$] 188.1077 (error 3.72 ppm). NMR yield was calculated to be 43%.

Synthesis of 2-(4-hydroxyphenyl)acetonitrile, **10**

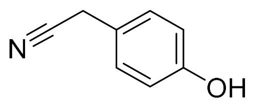

General procedure for reactions in Lucent360 was followed, irradiating for 88 hours. The reaction mixture was diluted in DCM, adsorbed onto Florisil® and purified by column chromatography (10-25% EtOAc in cyclohexane over 50 CV, 12 g RediSep Silica column, solid loaded). Fractions containing product were

combined and solvent removed under reduced pressure at 30 °C to give product as a white powder (11.1 mg, 28%). LCMS (2 minute high pH method): t_R = 0.62 min, $[M-H]^-$ 132 (100% purity). ^1H NMR (400 MHz, $CDCl_3$) δ ppm 7.15 - 7.20 (m, 2H), 6.81 - 6.86 (m, 2H), 5.21 (br s, 1H), 3.67 (s, 2H). ^{13}C NMR (101 MHz, $CDCl_3$) δ ppm 155.5, 129.3, 121.8, 118.2, 116.1, 22.9. Characterisation data is consistent with literature reports. [9] NMR yield was calculated to be 26%.

Synthesis of 2-(2-benzyl-1,1-dioxido-3-oxo-3,4-dihydro-2*H*-benzo[*e*][1,2,4]thiadiazin-7-yl)acetonitrile, **3**

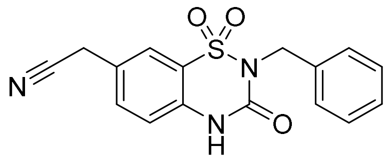

General procedure for reactions in Lucent360 was followed, irradiating for 22 hours. The reaction mixture was diluted in DCM, adsorbed onto Florisil® and purified by column chromatography (10-50% EtOAc in cyclohexane over 50 CV, 12 g RediSep Silica column). Fractions containing product were combined and solvent removed under reduced pressure at 30 °C to give product as a white powder (25.1 mg, 25%). LCMS (2 minute high pH method): t_R = 0.70 min, $[M-H^-]$ 326, (98% purity). ^1H NMR (400 MHz, DMSO-d_6) δ ppm 11.24 - 11.61 (br s, 1H), 7.76 (d, *J* = 2.0 Hz, 1H), 7.59 (dd, *J* = 8.4, 2.0 Hz, 1H), 7.12 - 7.23 (m, 6H), 4.86 (s, 2H), 4.01 (s, 2H). DCM is present in spectrum, equating to approximately 2 wt %. ^{13}C NMR (101 MHz, DMSO-d_6) δ ppm 150.0, 136.9, 135.2, 134.5, 128.9, 128.2, 128.0, 127.4, 122.7, 122.0, 119.2, 118.5, 44.1, 22.1. $\tilde{\nu}_{max}$ ($CDCl_3$ solution) = 3257, 3067, 2925, 2256, 1699, 1507, 1334, 1169, 826, 731 cm^{-1}. HRMS (20 minute high pH): ($C_{16}H_{14}N_3O_3S$) $[M+H^+]$ requires 328.0750, found $[M+H^+]$ 328.0741 (error -2.74 ppm). NMR yield was calculated to be 28%.

Synthesis of 2-(3-(difluoromethoxy)phenyl)acetonitrile, **11**

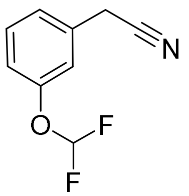

General procedure for reactions in Lucent360 was followed, irradiating for 22 hours. The reaction mixture was diluted in DCM, adsorbed onto Florisil® and purified by column chromatography (0-30% EtOAc in cyclohexane over 50 CV, 12 g RediSep Silica column, solid loaded). Fractions containing product were combined and solvent removed under reduced pressure at 30 °C to give product as a clear, colourless liquid (14.1 mg, 25%). LCMS (2 minute high pH method): t_R = 0.91 mins, $[M-H]^-$ 182, (95% purity). ^1H NMR (400 MHz, $CDCl_3$) δ ppm 7.39 - 7.44 (m, 1H), 7.21 - 7.25 (m, 1H), 7.11 - 7.15 (m, 2H), 6.55 (t, *J* = 72.9 Hz, 1H), 3.79 (s, 2H). ^{13}C NMR (151 MHz, $CDCl_3$) δ ppm 151.5 (br d, *J* = 5.5 Hz), 131.9, 130.6, 124.9, 119.4, 119.3, 117.2, 115.6 (t, *J* = 260.9 Hz), 23.5. ^{19}F NMR (376 MHz, $CDCl_3$) δ ppm -81.11 (d, *J* = 73.2 Hz, 1F). $\tilde{v}_{max}$ ($CDCl_3$ solution) = 2923, 2254, 1613, 1592, 1490, 1382, 1244, 1116, 1037, 774, 690 cm^{-1}. HRMS (10 minute formic acid): ($C_{10}H_9N_2O$) $[M-H^-]$ requires 173.0720, found $[M-H^-]$ 173.0415 (error -4.39 ppm). NMR yield was calculated to be 43%.

Synthesis of 3-(cyanomethyl)-N-methylbenzamide, **12**

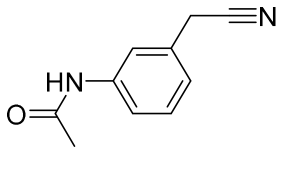

General procedure for reactions in Lucent360 was followed, irradiating for 66 hours. The reaction mixture was diluted in DCM, adsorbed onto Florisil® and purified by column chromatography (5-50% EtOAc in cyclohexane over 50 CV, 12 g RediSep Silica column, solid loaded). Fractions containing product were combined and solvent removed under reduced pressure at 30 °C to give product as a white solid (19.0 mg, 36% yield, 100% purity by LCMS). t_R = 0.64 mins $[M+H]^+$ 175, $[M-H]^-$ 173. ^{1}H NMR (400 MHz, $CDCl_3$) δ ppm 7.67 (br s, 1H), 7.56 (s, 1H), 7.44 (br d, *J* = 7.8 Hz, 1H), 7.30 (t, *J* = 7.8 Hz, 1H), 7.06 (br d, *J* = 7.8 Hz, 1H), 3.71 (s, 2H), 2.17 (s, 3H). ^{13}C NMR (101 MHz, $CDCl_3$) δ ppm 168.7, 138.8, 130.8, 129.8, 123.6, 119.4, 119.3, 117.8, 24.5, 23.6. $\tilde{v}_{max}$ ($CDCl_3$ solution) = 3265, 3106, 2920, 2247, 1662, 1596, 1559, 1439, 1371, 788, 764, 690 cm^{-1}. HRMS (10 minute formic acid): ($C_{10}H_9N_2O$) $[M-H^-]$ requires 173.0720, found $[M-H^-]$ 173.0718 (error -1.16 ppm). NMR yield was calculated to be 60%.

Synthesis of 2-(4-fluoronaphthalen-1-yl)acetonitrile, **13**

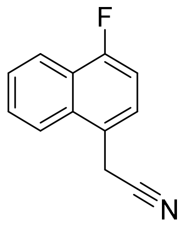

General procedure for reactions in Lucent360 was followed, irradiating for 66 hours. The reaction mixture was diluted in DCM, adsorbed onto Florisil® and purified by column chromatography (1-5% EtOAc in cyclohexane over 50 CV, 12 g RediSep Silica column, solid loaded). Fractions containing product were combined and solvent removed under reduced pressure at 30 °C to give crude product. Crude product was purified by reverse-phase column chromatography (30-85% acetonitrile in 10 mM ammonium bicarbonate in water (adjusted to pH 10 with ammonia solution) over 10 mins at 40 mL/min, Waters XSelect CSH Prep C18 5 µm, 30 x 100 mm). Fractions containing product were combined and acetonitrile was

removed under reduced pressure at 30 °C. Product was extracted into DCM and solvent removed under reduced pressure at 30 °C to give product as off-white crystals (12.3 mg, 22%). LCMS (2 minute high pH method): t_R = 1.08 min, $[M-H]^-$ 184 (100% purity). ^1H NMR (400 MHz, $CDCl_3$) δ ppm 8.19 (dd, *J* = 8.2, 1.6 Hz, 1H), 7.87 (d, *J* = 8.4 Hz, 1H), 7.60 - 7.70 (m, 2H), 7.51 (dd, *J* = 8.0, 5.0 Hz, 1H), 7.13 (dd, *J* = 10.0, 8.0 Hz, 1H), 4.08 (s, 2H). ^{13}C NMR (101 MHz, $CDCl_3$) δ ppm 159.1 (d, *J* = 254.8 Hz), 132.0 (d, *J* = 4.6 Hz), 128.1, 126.7, 126.4 (d, *J* = 9.2 Hz), 124.1 (d, *J* = 16.8 Hz), 122.5 (d, *J* = 3.1 Hz), 121.7, 121.6, 117.5, 109.0 (d, *J* = 21.4 Hz), 21.4. ^{19}F NMR (376 MHz, $CDCl_3$) δ -121.72 - -121.63 (m). $\tilde{\nu}_{max}$ ($CDCl_3$ solution) = 3069, 2913, 2254, 1604, 1466, 1397, 1260, 1053, 826, 755 cm^{-1}. HRMS (10 minute formic acid): ($C_{12}H_7FN$) $[M-H^-]$ requires 184.0568, found $[M-H^-]$ 184.0602 (error 18.47 ppm). NMR yield was calculated to be 36%.

Synthesis of *tert*-butyl 5-(cyanomethyl)isoindoline-2-carboxylate, **14**

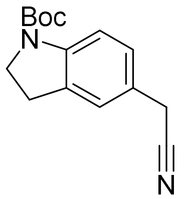

General procedure for reactions in Lucent360 was followed, irradiating for 88 hours. The reaction mixture was diluted in DCM, adsorbed onto Florisil® and purified by column chromatography (1-10% EtOAc in cyclohexane over 60 CV, 12 g RediSep Silica column, solid loaded). Fractions containing product were combined and solvent removed under reduced pressure at 30 °C to give product as an off-white crystalline solid (26.1 mg, 33%). LCMS (2 minute high pH method): t_R = 1.23 min, $[M-H]^-$ 257, $[M-Boc+H]^+$ 159 (98% purity). ^1H NMR (600 MHz, DMSO-d_6, 298 K) δ ppm 7.67 (br s, 1H), 7.16 (br s, 1H), 7.11 (d, *J* = 8.4 Hz, 1H), 3.92 (s, 2H), 3.89 (t, *J* = 8.8 Hz, 2H), 3.05 (t, *J* = 8.8 Hz, 2H), 1.50 (br s, 9H). ^1H NMR (600

MHz, DMSO-d_6, 373 K) δ ppm 7.59 (br d, J = 8.1 Hz, 1H), 7.17 (br s, 1H), 7.12 (br d, J = 7.3 Hz, 1H), 3.94 (t, J = 9.0 Hz, 2H), 3.88 (s, 2H), 3.08 (t, J = 8.6 Hz, 2H), 1.54 (s, 9H). ^{13}C NMR (101 MHz, DMSO-d_6) δ ppm 152.3, 142.5, 132.9, 127.3, 125.1, 125.0, 119.4, 114.7, 80.9, 48.1, 28.6, 27.2, 22.4. $\tilde{\nu}_{max}$ ($CDCl_3$ solution) = 2976, 2931, 2250, 1694, 1492, 1389, 1142, 1020, 821, 765 cm^{-1}. HRMS (10 minute formic acid): ($C_{15}H_{19}N_2O_2$) [M-^{t}Bu+H$^+$] requires 203.0815, found [M-^{t}Bu+H$^+$] 203.0814 (error -0.49 ppm). NMR yield was calculated to be 40%.

Synthesis of 2-(2-methylbenzo[d]thiazol-5-yl)acetonitrile, **15**

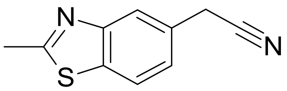

General procedure for reactions in Lucent360 was followed, irradiating for 22 hours. The reaction mixture was diluted in DCM, adsorbed onto Florisil® and purified by column chromatography (10-30% EtOAc in cyclohexane over 50 CV, 12 g RediSep Silica column). Fractions containing product were combined and solvent removed under reduced pressure at 30 °C to give product as a white powder (20.2 mg, 35%). LCMS (2 minute high pH method): t_R = 0.79 min, [M+H$^+$] 189, (99% purity). ^{1}H NMR (400 MHz, $CDCl_3$) δ ppm 7.89 (br s, 1H), 7.82 (d, J = 8.1 Hz, 1H) 7.32 (dd, J = 8.2, 1.6 Hz, 1H), 3.88 (s, 2H), 2.84 (s, 3H). ^{13}C NMR (101 MHz, $CDCl_3$) δ ppm 168.5, 153.9, 135.5, 128.0, 124.4, 122.1, 121.9, 117.7, 23.6, 20.2. Characterisation data is consistent with literature reports. [8] NMR yield was calculated to be 61%.

Synthesis of 2-(quinolin-6-yl)acetonitrile, **16**

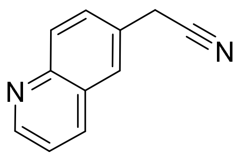

General procedure for reactions in Lucent360 was followed, irradiating for 22 hours. The reaction mixture was dissolved in DCM, adsorbed onto Florisil® and purified by column chromatography (10-100% EtOAc in cyclohexane over 50 CV, 12 g RediSep Silica column). 1% EtOAc (10 CV) was flowed through the column prior to the gradient starting, in order to remove silanol-related species. Fractions containing product were combined and solvent removed under reduced pressure at 30 °C to give product as a yellow gummy solid (15.3 mg, 29%). LCMS (2 minute high pH method): t_R = 0.70 min, [$M+H^+$] 169, (97% purity). ^{1}H NMR (400 MHz, $CDCl_3$) δ ppm 8.92 - 8.97 (m, 1H), 8.10 - 8.20 (m, 2H), 7.84 (br s, 1H), 7.59 - 7.64 (m, 1H), 7.42 - 7.48 (m, 1H), 3.94 - 3.97 (m, 2H). ^{13}C NMR (101 MHz, $CDCl_3$) δ ppm 151.0, 147.7, 135.9, 130.6, 129.1, 128.3, 128.2, 126.7, 121.9, 117.4, 23.7. Characterisation data is consistent with literature reports.[8] NMR yield was calculated to be 30%.

Synthesis of 2-(quinolin-4-yl)acetonitrile, **17**

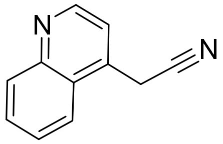

General procedure for reactions in Lucent360 was followed, irradiating for 22 hours. The reaction mixture was diluted in DCM, adsorbed onto Florisil® and purified by column chromatography (20-30% EtOAc in cyclohexane over 50 CV, 12 g RediSep Silica column, solid loaded). 1% EtOAc (10 CV) was flowed through the column prior to the gradient starting, in order to remove silanol-related species. Fractions containing product were combined and solvent removed under reduced pressure at 30 °C to give crude

product. Crude product was purified by reverse-phase column chromatography (15-55% acetonitrile in 10 mM ammonium bicarbonate in water (adjusted to pH 10 with ammonia solution) over 10 mins at 40 mL/min, Waters XSelect CSH Prep C18 5 µm, 30 x 100 mm). Fractions containing product were combined and acetonitrile was removed under reduced pressure at 30 °C. Product was extracted into DCM and solvent removed under reduced pressure at 30 °C to give product as a yellow solid (9.2 mg, 18%). LCMS (2 minute high pH method): t_R = 0.76 min, [M+H$^+$] 169, (98% purity). ^{1}H NMR (400 MHz, $CDCl_3$) δ ppm 8.94 (d, *J* = 4.4 Hz, 1H), 8.19 (dd, *J* = 8.4, 1.9 Hz, 1H), 7.87 (dd, *J* = 8.4, 2.0 Hz, 1H), 7.88 – 7.82 (m, 1H), 7.63 - 7.70 (m, 1H), 7.56 (dt, *J* = 4.4, 1.0 Hz, 1H), 4.18 (d, *J* = 1.0 Hz, 2H). ^{13}C NMR (101 MHz, $CDCl_3$) δ ppm 150.3, 148.2, 135.5, 130.7, 130.0, 127.7, 125.9, 122.0, 120.7, 116.2, 21.2. $\tilde{\nu}_{max}$ ($CDCl_3$ solution) = 2931, 2253, 1511, 1395, 825, 758 cm^{-1}. HRMS (10 minute formic acid): ($C_{11}H_7N_2$) [M-H$^-$] requires 167.0615, found [M-H$^-$] 167.0614 (error -0.60 ppm). NMR yield was calculated to be 9.8%.

Synthesis of 2-(isoquinolin-4-yl)acetonitrile, **18**

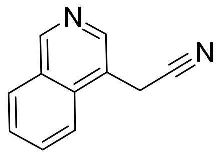

General procedure for reactions in Lucent360 was followed, irradiating for 22 hours. The reaction mixture was diluted in DCM, adsorbed onto Florisil® and purified by column chromatography (20-30% EtOAc in cyclohexane over 50 CV, 12 g RediSep Silica column, solid loaded). 1% EtOAc (10 CV) was flowed through the column prior to the gradient starting, in order to remove silanol-related species. Fractions containing product were combined and solvent removed under reduced pressure at 30 °C to give crude product. Crude product was purified by reverse-phase column chromatography (15-55% acetonitrile in 10 mM ammonium bicarbonate in water (adjusted to pH 10 with ammonia solution) over 10 mins at 40

mL/min, Waters XSelect CSH Prep C18 5 µm, 30 x 100 mm). Fractions containing product were combined and acetonitrile was removed under reduced pressure at 30 °C. Product was extracted into DCM and solvent removed under reduced pressure at 30 °C to give product as a yellow solid (11.4 mg, 22%). LCMS (2 minute high pH method): t_R = 0.77 min, [M+H$^+$] 169, (98% purity). ^{1}H NMR (400 MHz, $CDCl_3$) δ ppm 9.27 (s, 1H), 8.58 (s, 1H), 8.05 – 8.08 (m, 1H), 7.92 – 7.96 (m, 1H), 7.83 – 7.88 (m, 1H), 7.69 – 7.74 (m, 1H), 4.08 (s, 2H). ^{13}C NMR (101 MHz, $CDCl_3$) δ ppm 153.9, 142.9, 133.5, 131.6, 128.7, 128.3, 127.9, 121.8, 119.9, 116.8, 19.1. $\tilde{\nu}_{max}$ ($CDCl_3$ solution) = 2934, 2904, 2242, 748 cm^{-1}. HRMS (10 minute formic acid): ($C_{11}H_7N_2$) [M-H$^-$] requires 167.0615, found [M-H$^-$] 167.0615 (error 0.00 ppm). NMR yield was calculated to be 31%.

Synthesis of methyl 6-(cyanomethyl)-2-naphthoate, **19**

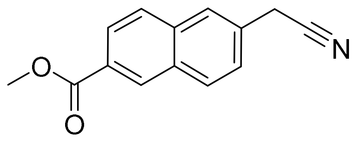

General procedure for reactions in Lucent360 was followed, irradiating for 44 hours. The reaction mixture was diluted in DCM, adsorbed onto Florisil® and purified by column chromatography (5-15% EtOAc in cyclohexane over 50 CV, 12 g RediSep Silica column, solid loaded). 1% EtOAc (10 CV) was flowed through the column prior to the gradient starting, in order to remove silanol-related species. Fractions containing product were combined and solvent removed under reduced pressure at 30 °C to give crude product. Crude product was purified by reverse-phase column chromatography (30-85% acetonitrile in 10 mM ammonium bicarbonate in water (adjusted to pH 10 with ammonia solution) over 10 mins at 40 mL/min, Waters XSelect CSH Prep C18 5 µm, 30 x 100 mm). Fractions containing product were combined and acetonitrile was removed under reduced pressure at 30 °C. Product was extracted into DCM

and solvent removed under reduced pressure at 30 °C to give product as an off-white solid (14.5 mg, 21%). LCMS (2 minute high pH method): t_R = 1.04 min, $[M-H]^-$ 224, (100% purity). ^1H NMR (400 MHz, $CDCl_3$) δ ppm 8.60 (br s, 1H), 8.10 (dd, J = 8.5, 1.7 Hz, 1H), 7.96 (d, J = 8.5 Hz, 1H), 7.85 - 7.89 (m, 2H), 7.45 (dd, J = 8.5, 1.8 Hz, 1H), 3.99 (s, 3H), 3.94 (s, 2H). ^{13}C NMR (101 MHz, $CDCl_3$) δ ppm 166.9, 135.4, 131.9, 130.8, 130.5, 129.8, 128.2, 128.0, 126.7, 126.3, 126.2, 117.4, 52.3, 24.0. $\tilde{v}_{max}$ ($CDCl_3$ solution) = 2961, 2933, 2257, 1725, 1294, 1180, 804 cm^{-1}. HRMS (10 minute formic acid): ($C_{14}H_{10}NO_2$) $[M-H^-]$ requires 224.0717, found $[M-H^-]$ 224.0726 (error 4.02 ppm). NMR yield was calculated to be 27%.

48 mmol scale reaction

Inside a nitrogen-filled purgebox, sodium carbonate (15.26 g, 144 mmol, 3.0 equiv.) was weighed into a Schott bottle. Methyl 6-bromo-2-naphthoate (98% purity, 12.99 g, 48 mmol, 1.0 equiv.) and $[Ir(dtbpy)(ppy)_2]PF_6$ (373 mg, 408 µmol, 0.0085 equiv.) in acetonitrile (180 mL), followed by tris(trimethylsilyl)silanol (16.3 mL, 52.8 µmol, 1.1 equiv.) were added to a separate Schott bottle. A solution of nickel(II) chloride ethylene glycol dimethyl ether complex (316 mg, 1.44 mmol, 0.03 equiv.) and 4,7-dimethoxy-1,10-phenanthroline (346 mg, 1.44 mmol, 0.03 equiv.) in acetonitrile (300 mL) was pre-mixed for 15 mins, then added to the reaction mixture Schott bottle, followed by cyclopropyl bromide (11.5 mL, 144 mmol, 3.0 equiv.). The Schott bottles were sealed and removed from the purgebox. Using a nitrogen-filled glovebag to maintain a nitrogen atmosphere, the sodium carbonate was added to a 500 mL reactor ready vessel, followed by the reaction mixture solution. The reactor vessel was equipped with a water-cooled condenser, a temperature probe and overhead stirring and was under a blanket of nitrogen. The reaction temperature was set to 40 °C, with stirring at 500 rpm. The reaction was irradiated by 6 x 440 nm PR160L Kessil lamps positions around the reactor, on the full intensity setting. The reaction was monitored by LCMS periodically over a total reaction time of 190 hours, and the reaction color was noted at each time point. The reaction mixture was adsorbed onto Florisil and purified by column

chromatography (5-15% EtOAc in cyclohexane over 50 CV, 12 g RediSep Silica column, solid loaded). 1% EtOAc (10 CV) was flowed through the column prior to the gradient starting, in order to remove silanol-related species. Fractions containing product were combined and solvent removed under reduced pressure at 30 °C to give product as a pale orange solid (3.176 g, 28.8%). Characterization data was consistent with the reaction performed on a 0.3 mmol scale. M.pt. 120 °C. Metal content was analyzed using a PANalytical Epsilon4 (firmware version 2.1.2), and Omnian software, which determined that the product contained 0.8 ppm Ni and 2.8 ppm Ir.

Synthesis of methyl 6-(cyanomethyl)picolinate, **20**

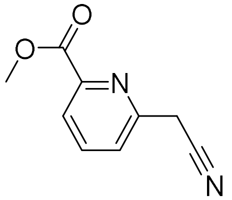

General procedure for reactions in Lucent360 was followed, irradiating for 22 hours. The reaction mixture was diluted in DCM, adsorbed onto Florisil® and purified by column chromatography (10-30% EtOAc in cyclohexane over 60 CV, 12 g RediSep Silica column, solid loaded). Fractions containing product were combined and solvent removed under reduced pressure at 30 °C to give crude product. Crude product was purified by reverse-phase column chromatography (30-85% acetonitrile in 10 mM ammonium bicarbonate in water (adjusted to pH 10 with ammonia solution) over 10 mins at 40 mL/min, Waters XSelect CSH Prep C18 5 µm, 30 x 100 mm). Fractions containing product were combined and acetonitrile was removed under reduced pressure at 30 °C. Product was extracted into DCM and solvent removed under reduced pressure at 30 °C to give product as a colourless liquid (2.9 mg, 5.4%). LCMS (2 minute high pH method): t_R = 0.59 min, $[M+H]^+$ 177, (98% purity). ^1H NMR (400 MHz, $CDCl_3$) δ ppm 8.14 (d, J = 7.9

Hz, 1H), 7.95 (t, J = 7.9 Hz, 1H), 7.74 (d, J = 7.9 Hz, 1H), 4.09 (s, 2H), 4.04 (s, 3H). ^{13}C NMR (101 MHz, $CDCl_3$) δ ppm 165.0, 151.1, 148.3, 138.6, 125.4, 124.5, 116.6, 53.1, 26.7. $\tilde{\nu}_{max}$ ($CDCl_3$ solution) = 2955, 2924, 2253, 1724, 1590, 1321, 1138, 995, 760 cm^{-1}. HRMS (10 minute formic acid): ($C_9H_9N_2O_2$) [$M+H^+$] requires 177.0659, found [$M+H^+$] 177.0664 (error 2.82 ppm). NMR yield was calculated to be 29%.

Synthesis of methyl 2-(cyanomethyl)benzoate, **21**

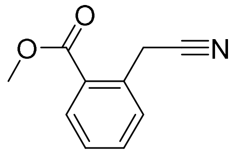

General procedure for reactions in Lucent360 was followed, irradiating for 22 hours. The reaction mixture was diluted in DCM, adsorbed onto Florisil® and purified by column chromatography (1-10% EtOAc in cyclohexane over 50 CV, 12 g RediSep Silica column, solid loaded). 1% EtOAc (10 CV) was flowed through the column prior to the gradient starting, in order to remove silanol-related species. Fractions containing product were combined and solvent removed under reduced pressure at 30 °C to give crude product. Crude product was purified by reverse-phase column chromatography (30-85% acetonitrile in 10 mM ammonium bicarbonate in water (adjusted to pH 10 with ammonia solution) over 12 mins at 40 mL/min, Waters XSelect CSH Prep C18 5 μm, 30 x 100 mm). Fractions containing product were combined and acetonitrile was removed under reduced pressure at 30 °C. Product was extracted into DCM and solvent removed under reduced pressure at 30 °C to give product as a white solid (14.1 mg, 27%). LCMS (2 minute high pH method): t_R = 0.92 min, [$M+H^+$] 175, (99% purity). ^{1}H NMR (400 MHz, $CDCl_3$) δ ppm 8.05 – 8.09 (m, 1H), 7.58 – 7.59 (m, 1H), 7.57 - 7.58 (m, 1H), 7.40 - 7.46 (m, 1H), 4.22 (s, 2H), 3.93 (s, 3H). ^{13}C NMR (101 MHz, $CDCl_3$) δ ppm 166.7, 133.1, 132.1, 131.6, 130.2, 128.3 (2C), 117.9,

52.3, 23.2. Characterisation data is consistent with literature reports. [10] NMR yield was calculated to be 44%.

Synthesis of 2-(2-propoxyphenyl)acetonitrile, **22**

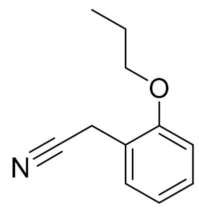

General procedure for reactions in Lucent360 was followed, irradiating for 22 hours. The reaction mixture was diluted in DCM, adsorbed onto Florisil® and purified by column chromatography (0-20% EtOAc in cyclohexane over 50 CV, 12 g RediSep Silica column, solid loaded). Fractions containing product were combined and solvent removed under reduced pressure at 30 °C. The resulting material was dissolved in DMSO and purified by reverse-phase column chromatography (30-85% acetonitrile in 10 mM ammonium bicarbonate in water (adjusted to pH 10 with ammonia solution) over 50 CV, 13 g Biotage C18 column). Fractions containing product were combined and acetonitrile was removed under reduced pressure at 30 °C, leaving an aqueous solution. The product was extracted in DCM and the DCM was removed under reduced pressure at 30 °C to give product as a clear, colourless liquid (10.1 mg, 19%). LCMS (2 minute high pH method): t_R = 1.12 min, $[M-H]^-$ 174, (100% purity). ^{1}H NMR (400 MHz, $CDCl_3$) δ ppm 7.33 – 7.37 (m, 1H), 7.26 – 7.31 (m, 1H), 6.95 (td, *J* = 7.5, 1.2 Hz, 1H), 6.87 (dd, *J* = 8.1, 0.7 Hz, 1H), 3.97 (t, *J* = 6.4 Hz, 2H), 3.69 (s, 2H), 1.85 (sxt, *J* = 7.0 Hz, 2H), 1.07 (t, *J* = 7.4 Hz, 3H). ^{13}C NMR (101 MHz, $CDCl_3$) δ ppm 156.3, 129.5, 129.2, 120.6, 118.8, 118.1, 111.2, 69.7, 22.6, 18.7, 10.6. $\tilde{v}_{max}$ ($CDCl_3$ solution) = 2966, 2878, 2252, 1602, 1495, 1455, 1252, 980, 751 cm^{-1}. HRMS (20 minute high pH):

($C_{11}H_{12}NO$) [M-H$^-$] requires 174.0924, found [M-H$^-$] 174.0880 (error -25.27ppm). NMR yield was calculated to be 52%.

Synthesis of 2-(3-chloro-5-isopropoxyphenyl)acetonitrile, **23**

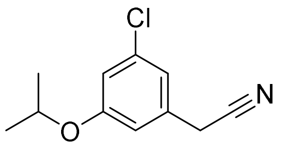

General procedure for reactions in Lucent360 was followed, irradiating for 22 hours. The reaction mixture was diluted in DCM, adsorbed onto Florisil® and purified by column chromatography (0-10% EtOAc in cyclohexane over 50 CV, 12 g RediSep Silica column, solid loaded). Fractions containing product were combined and solvent removed under reduced pressure at 30 °C to give crude product. Crude product was purified by reverse-phase column chromatography (50-99% acetonitrile in 10 mM ammonium bicarbonate in water (adjusted to pH 10 with ammonia solution) over 10 mins at 40 mL/min, Waters XSelect CSH Prep C18 5 µm, 30 x 100 mm). Fractions containing product were combined and acetonitrile was removed under reduced pressure at 30 °C. Product was extracted into DCM and solvent removed under reduced pressure at 30 °C to give product as a pale pink liquid (15.2 mg, 23%). LCMS (2 minute high pH method): t_R = 1.23 min, no ionisation observed (95% purity). ^{1}H NMR (400 MHz, $CDCl_3$) δ ppm 6.86 - 6.88 (m, 1H), 6.84 (t, J = 2.0 Hz, 1H), 6.73 - 6.76 (m, 1H), 4.53 (spt, J = 6.1 Hz, 1H), 3.66 - 3.69 (m, 2H), 1.33 (d, J = 6.1 Hz, 6H). ^{13}C NMR (101 MHz, $CDCl_3$) δ ppm 159.2, 135.6, 132.4, 120.0, 117.1, 115.6, 114.1, 70.7, 23.4, 21.9. $\tilde{v}_{max}$ ($CDCl_3$ solution) = 2979, 2931, 2256, 1577, 1452, 1270, 1114, 1016, 856, 833 cm^{-1}. HRMS (10 minute formic acid): ($C_{11}H_{11}ClNO$) [M-H$^-$] requires 208.0535, no mass ion found. NMR yield was calculated to be 40%.

Synthesis of 4-(cyanomethyl)benzenesulfonamide, **24**

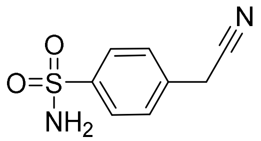

General procedure for reactions in Lucent360 was followed, irradiating for 66 hours. The reaction mixture was filtered and purified by reverse-phase column chromatography (3-22% methanol in 10 mM ammonium bicarbonate in water (adjusted to pH 10 with ammonia solution) over 14 mins at 40 mL/min, Waters XBridge BEH C18 5 μm, 30 x 100 mm). Fractions containing product were combined and methanol was removed under reduced pressure at 30 °C. The aqueous solution was freeze dried to give product as a white fluffy solid (11.0 mg, 18%). LCMS (2 minute high pH method): t_R = 0.47 min, $[M-H]^-$ 195, (98% purity). ^{1}H NMR (400 MHz, DMSO-d_6) δ ppm 7.85 (d, *J* = 8.4 Hz, 2H), 7.55 (d, *J* = 8.4 Hz, 2H), 7.20 - 7.43 (br s, 2H), 4.17 (s, 2H). ^{13}C NMR (101 MHz, DMSO-d_6) δ ppm 143.9, 135.8, 129.1, 126.8, 119.2, 22.8. $\tilde{v}_{max}$ ($CDCl_3$ solution) = 3361, 3254, 2251, 1568, 1337, 1147 cm^{-1}. HRMS (10 minute formic acid): ($C_8H_7N_2O_2S$) $[M-H^-]$ requires 195.0234, found $[M-H^-]$ 195.0233 (error -0.51 ppm). NMR yield was calculated to be 7.3%.

Synthesis of methyl 2-(cyanomethyl)pyrimidine-4-carboxylate, **25**

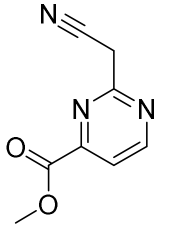

General procedure for reactions in Lucent360 was followed, irradiating for 66 hours. Low levels of product were formed in the reaction. The reaction mixture was filtered and purification was attempted by reverse-phase column chromatography (30-99% acetonitrile in 10 mM ammonium bicarbonate in water (adjusted to pH 10 with ammonia solution) over 25 mins at 30 mL/min, an Xselect CSH C18 column (150mm x 30mm i.d. 5µm). Product was not recovered from the purification procedure. NMR yield was calculated to be 9.3%.

Synthesis of 2-(pyrimidin-2-yl)acetonitrile, **26**

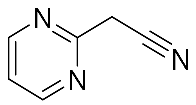

General procedure for reactions in Lucent360 was followed, irradiating for 44 hours. Trace amounts of product[11] was formed in the reaction, therefore purification was not attempted. NMR yield was calculated to be 7.7%.

Synthesis of 2-(thiophen-2-yl)acetonitrile, **27**

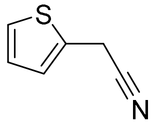

General procedure for reactions in Lucent360 was followed, irradiating for 22 hours. Trace amounts of product[12] was formed in the reaction, therefore purification was not attempted. NMR yield was calculated to be 8.8%.

Synthesis of 2-(pyridin-2-yl)acetonitrile, **28**

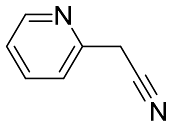

General procedure for reactions in Lucent360 was followed, irradiating for 22 hours. Trace amounts of product[13] was formed in the reaction, therefore purification was not attempted. NMR yield was calculated to be 27%.

Synthesis of 6-(3-(cyanomethyl)phenyl)-2-oxo-1,2-dihydropyridine-3-carbonitrile, **29**

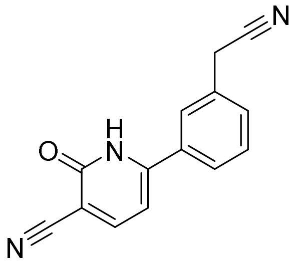

General procedure for reactions in Lucent360 was followed, irradiating for 66 hours. Low levels of product were formed in the reaction. The reaction mixture was filtered and purification was attempted by reverse-phase column chromatography (30-51% methanol in 10 mM ammonium bicarbonate in water (adjusted to pH 10 with ammonia solution) over 8.5 mins at 20 mL/min, an XBridge Prep C18 column (150 x 30mm, 5µm)). Solvents were removed under reduced pressure at 30 °C to give product as a white amorphous solid (2.7 mg, 2.9%). LCMS (2 minute high pH method): t_R = 0.59 min, $[M+H]^+$ 236 (purity could not be calculated due to co-eluting dehalogenated starting material impurity present). ^{1}H NMR

shows dehalogenated material equates to approximately 25 mol % of the sample. NMR yield was calculated to be 7.2%.

Synthesis of methyl 2-(1-methyl-1*H*-imidazol-2-yl)acetonitrile, **50**

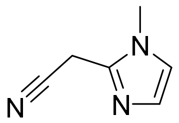

General procedure for reactions in Lucent360 was followed. Reaction failed to produce desired product.

Synthesis of 2-(1,2,3,4-tetrahydroisoquinolin-5-yl)acetonitrile, **51**

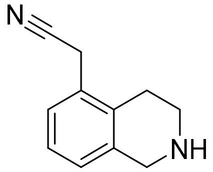

General procedure for reactions in Lucent360 was followed. Reaction failed to produce desired product.

Synthesis of 2-(3-chloro-5-(piperidin-1-ylmethyl)phenyl)acetonitrile, **52**

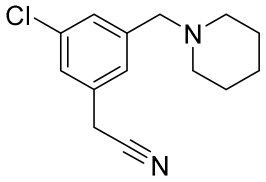

General procedure for reactions in Lucent360 was followed. Reaction failed to produce desired product.

Cyanomethylation of bromodiphenhydramine, **53**

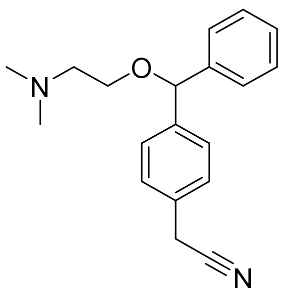

General procedure for reactions in Lucent360 was followed. Reaction failed to produce desired product.

Cyanomethylation of amiodarone, **54**

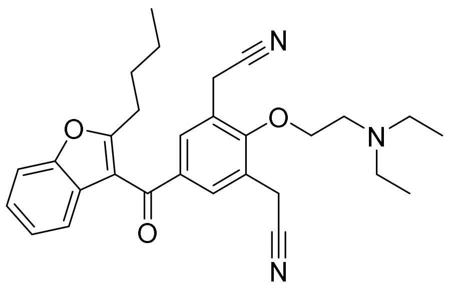

General procedure for reactions in Lucent360 was followed. Reaction failed to produce desired product.

Cyanomethylation of selumetinib, **55**

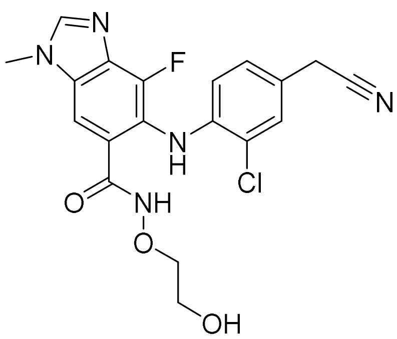

General procedure for reactions in Lucent360 was followed. Reaction failed to produce desired product.

5. Mechanistic Investigations

5.1 Reactions performed in MeCN-d_3

Sodium carbonate (12.7 mg, 120 µmol, 2.0 equiv.) was weighed under nitrogen into 3 x 2 mL crimp-top HPLC vials. 4-bromoacetophenone (11.9 mg, 60 µmol, 1.0 equiv.), 1,1,1,3,3,3-hexamethyl-2-(trimethylsilyl)trisilan-2-ol (27.7 µL, 90 µmol, 1.5 equiv.) and MeCN-d_3 (0.3 mL) were then added to each vial under nitrogen. [Ir(ppy)$_2$(dtbpy)]PF$_6$ (0.5 mg, 0.6 µmol, 0.01 equiv.) was dissolved in 100 µL MeCN-d_3 and added to the vials. $NiCl_2$.glyme (0.7 mg, 3 µmol, 0.05 equiv.) and 4,7-dimethoxy-1,10-phenanthroline (0.6 mg, 2.4 µmol, 0.04 equiv.) was dissolved in 200 µL MeCN-d_3 and added to each vial. To one of the vials, cyclopropyl bromide (9.6 µL, 120 µmol, 2.0 equiv.) was added. To another vial, (2-bromocyclopropyl)benzene (23.7 mg, 120 µmol, 2.0 equiv.) was added. To the third vial, no alkyl bromide was added. The vials were sealed, removed from the glovebox and irradiated with an LED at 450 nm in the PHIL Pacer, with a light intensity of 300 mA, shaking using an orbital shaker at 150 rpm. The LED temperature typically reached 35 °C. Under nitrogen, a 1 in 2 dilution was performed into MeCN-d_3 for ^{1}H NMR analysis and a 1 in 10 dilution was performed into MeCN-d_3 for GCMS and LCMS analysis. LCMS (2 minute high pH method): t_R = 0.75 mins, $[M-H]^-$ 159. ^{1}H NMR of the crude reaction mixture (400 MHz, MeCN-d_3) δ ppm 7.78 - 7.86 (m, 2H), 7.49 - 7.59 (m, 2H), 2.41 (s, 3H). Benzylic protons were not

observed by NMR, suggesting only deuterium atoms are present at the benzylic position. Deuterated and non-deuterated reaction products were identified by GCMS (Table 24).

Table 24 Reaction products observed through GCMS analysis of reactions performed in MeCN-d3.

Retention time (mins)	Mass Observed	Reaction product	Notes
2.67	119.9	cyclopropyl bromide, **A1** 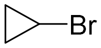	Observed in reactions with cyclopropyl bromide only
2.99	147.0 (-Me)	Hexamethyldisiloxane, **56** 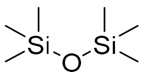	Observed in all reactions
6.12	118.0 & 117.0	cyclopropylbenzene-d, **33** & cyclopropyl benzene, **57** 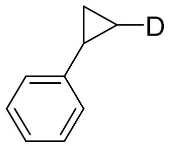 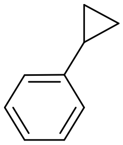	Observed in reactions with (2-bromocyclopropyl)benzene only
6.45	120.0	Acetophenone, **58** 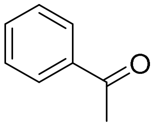	Observed in all reactions
7.30	280, 191 (-OTMS)	trimethylsilyl-di(trimethylsiloxy)-silane, **59** 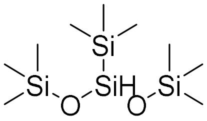	Observed in all reactions
7.81 & 7.99	117.0 (-Br)	(2-bromocyclopropyl)benzene, **30**	Observed in reactions with (2-bromocyclopropyl)benzene only

		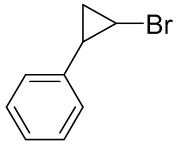	
8.03	191.1 (-TMS)	Supersilanol, **60** 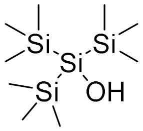	Observed in all reactions
8.10	197.9	Bromoacetophenone, **5** 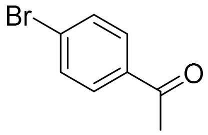	Observed in all reactions
8.55	326.0	$(TMS)_3SiBr$, **61** 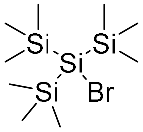	Observed in all reactions
9.23	161.0	deuterated nitrile product, **31** 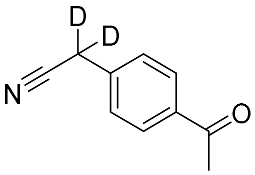	Observed in all reactions

Reference samples of the following compounds were also analysed by GCMS to confirm retention times: cyclopropyl benzene (**57**), (2-bromocyclopropyl)benzene (**30**), supersilanol (**60**), $(TMS)_3SiBr$ (**61**). All other GCMS signals have been assigned based on the mass observed and future work will involve isolating these species for confirmation.

Reaction performed in MeCN

Sodium carbonate (12.7 mg, 120 µmol, 2.0 equiv.) was weighed under nitrogen into a 2 mL crimp-top HPLC vial. 4-bromoacetophenone (11.9 mg, 60 µmol, 1.0 equiv.), 1,1,1,3,3,3-hexamethyl-2-

(trimethylsilyl)trisilan-2-ol (27.7 µL, 90 µmol, 1.5 equiv.) and MeCN (0.3 mL) were then added to the vial under nitrogen. $[Ir(ppy)_2(dtbpy)]PF_6$ (0.5 mg, 0.6 µmol, 0.01 equiv.) was dissolved in 100 µL MeCN and added to the vial. $NiCl_2$.glyme (0.7 mg, 3 µmol, 0.05 equiv.) and 4,7-dimethoxy-1,10-phenanthroline (0.6 mg, 2.4 µmol, 0.04 equiv.) was dissolved in 200 µL MeCN and added to the vial. Cyclopropyl bromide (9.6 µL, 120 µmol, 2.0 equiv.) was added and the vial was sealed, removed from the glovebox and irradiated with an LED at 450 nm in the PHIL Pacer, with a light intensity of 300 mA, shaking using an orbital shaker at 150 rpm. The LED temperature typically reached 35 °C. Under nitrogen, a 1 in 2 dilution was performed into MeCN-d_3 for ^{1}H NMR analysis and a 1 in 10 dilution was performed into MeCN for GCMS and LCMS analysis. LCMS (2 minute high pH method): t_R = 0.75 mins, $[M-H]^-$ 158. ^{1}H NMR of the crude reaction mixture (400 MHz, MeCN-d_3) δ ppm 7.78 - 7.88 (m, 2H), 7.29 - 7.40 (m, 2H), 3.75 (s, 2H), 2.41 (s, 3H). Deuterated and non-deuterated reaction products were identified by GCMS (Table 25).

Table 25 Reaction products observed through GCMS analysis of reactions performed in MeCN.

**Retention time (mins)**	**Mass Observed**	**Reaction product**
2.66	119.9	cyclopropyl bromide, **A1** 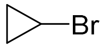
3.00	147.0 (-Me)	Hexamethyldisiloxane, **56** 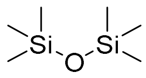
6.45	120.0	Acetophenone, **58** 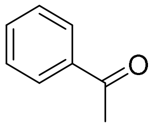
7.30	280, 191 (-OTMS)	trimethylsilyl-di(trimethylsiloxy)-silane, **59** 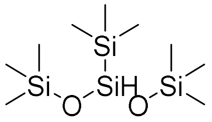
8.03	191.1 (-TMS)	Supersilanol, **60**

		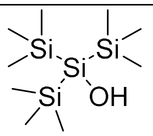
8.10	197.9	Bromoacetophenone, **5** 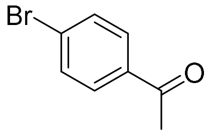
8.55	326.0	$(TMS)_3SiBr$, **61** 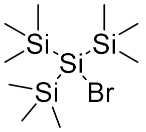
9.26	159.0	nitrile product, **4** 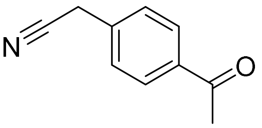

Synthesis of 2-bromo-1,1,1,3,3,3-hexamethyl-2-(trimethylsilyl)trisilane, **61**

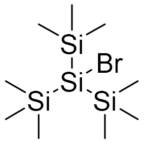

According to a modified literature procedure. [14] Isopropyl bromide (113 µL, 2 equiv., 1.20 mmol) was added dropwise to a solution of tris(trimethylsilyl)silane (185 µL, 1 equiv., 0.60 mmol) in tert-butyl methyl ether (0.2 mL) under air in a 4 mL borosilicate vial. The reaction vial was capped under air and irradiated with 450 nm LEDs for 12 hours, using the Lucent360 reactor (light intensity 100%, chiller set to 30 °C, stirring at 1000 rpm). Volatiles were removed under a stream of nitrogen at 40 °C, leaving product as a clear and colourless gum (124 mg, 60%). GCMS: t_R = 8.56 mins, [M] 326, (94.6% purity). ^{1}H NMR (400 MHz, $CDCl_3$) δ ppm 0.12 (s, 27H). ^{1}H NMR (400 MHz, MeCN-d_3) δ ppm 0.17 - 0.20 (m,

27H). ^{13}C NMR (101 MHz, MeCN-d_3) δ ppm 0.7. $\tilde{\nu}_{max}$ ($CDCl_3$ solution) = 2957, 2900, 1247, 1056, 841 cm^{-1}. HRMS unavailable as product was unstable to column conditions.

When supersilanol is used as an abstraction reagent in the literature, it is generally proposed that either a radical Brook rearrangement or β-scission process occurs. [15] If this was the case, detection of either the deuteration or bromination of these resulting radical species might be expected. None of these species were detected, however, the formation of **61** may suggest the formation of the supersilyl radical, rather than **63**. Further studies into this process are ongoing.

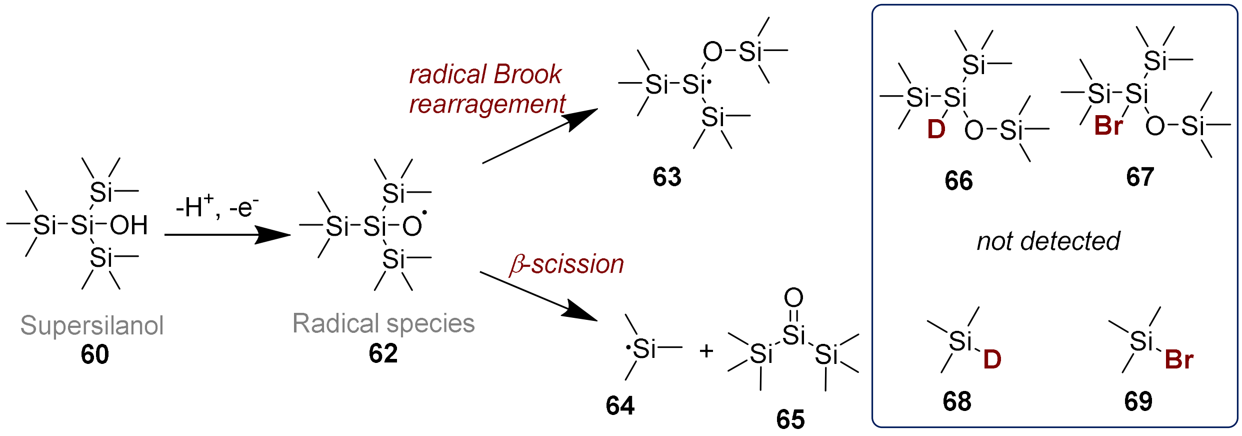

*Scheme 1 Literature proposed radical species which can be formed from supersilanol (**60**).*

5.2 Stern-Volmer Quenching

Validation of Procedure for Fluorescence Measurements

Inside a nitrogen-filled glovebox, a stock solution was prepared by dissolving $[Ir(ppy)_2(dtbpy)]PF_6$ (11.7 mg) in acetonitrile (12.75 mL). To 2 mL aliquots of this solution, varying volumes of DIPEA were added, according to Table 26. The solutions (100 µL) were added to the appropriate wells of a 96-well fluorescence microplate (black-walled, clear bottom), as described by Table 26 and Table 27. The plate was sealed with LightCycler 480 sealing foil and removed from the glovebox in a sealed plastic tub to maintain a nitrogen atmosphere until the plate is placed onto the microplate reader for analysis. Each well of the

plate was excited at 420 nm and emission was measured at 581 nm, using a SpectraMax M5 Multi-Mode Microplate Reader. Measurements were taken every 5 minutes over a 30 minute period.

Table 26 Iridium stock solutions with varying concentrations of DIPEA.

Stock Solution	Plate Columns	DIPEA Concentration (M)	DIPEA added (µL)
A	1 & 2	0	0
B	3 & 4	0.1	18
C	5 & 6	0.2	35
D	7 & 8	0.3	53
E	9 & 10	0.4	70
F	11 & 12	0.5	88

*Table 27 Fluorescence output of [Ir(ppy)$_2$(dtbpy)]PF$_6$ (**Ir 2**) at increasing concentrations of known quencher DIPEA, measured every 5 minutes over a period of 30 minutes. Fluorescence output refers to the detection of photons emitted from the sample at 581 nm.*

a) 0 minutes

DIPEA Concentration (M)											
0	0	0.1	0.1	0.2	0.2	0.3	0.3	0.4	0.4	0.5	0.5
3877	3843	179	176	94	94	66	63	47	48	40	41
3995	3818	176	174	92	94	64	66	48	49	40	40
4022	3824	177	175	93	94	66	64	48	48	40	40
4009	3793	176	175	92	92	65	66	48	47	41	41
3984	3762	175	177	90	91	65	64	47	47	39	40
4028	3879	175	174	89	92	65	64	49	48	40	41
4021	3778	177	174	90	91	64	64	48	48	39	41
4125	3859	174	171	91	92	63	64	47	49	39	39

b) 5 minutes

2098	3804	179	163	94	93	66	62	45	47	41	42
3946	3775	174	176	91	93	64	65	48	48	40	41
4014	3795	180	178	94	94	64	66	48	50	39	39
3963	3743	178	173	92	93	64	65	48	48	39	40
3950	3742	175	174	91	92	64	65	49	49	39	40
3996	3858	176	175	91	93	65	66	48	49	39	42
4022	3724	175	174	90	93	64	65	48	48	40	40
4095	3821	175	173	91	92	65	65	48	47	39	38

c) 10 minutes

1199	3716	171	156	88	94	66	60	46	46	42	41
3883	3717	178	177	93	90	63	64	48	48	40	40
2478	3742	180	181	95	95	65	65	47	49	40	39
3898	3680	178	176	92	94	65	65	48	47	39	39
3228	3687	174	177	93	92	64	64	47	48	39	39
3863	3795	179	178	92	93	63	65	48	49	40	40

3947	3714	176	177	90	93	63	64	49	50	40	39
4029	3682	175	173	94	93	65	64	49	49	38	40

d) 15 minutes

980	1073	157	153	85	92	66	60	46	46	41	41
3823	3619	176	176	93	89	63	63	47	46	40	41
1624	3648	180	178	93	95	66	64	47	50	40	39
3163	3602	176	175	92	95	64	64	48	47	39	38
2395	3589	174	176	91	92	63	65	47	49	39	39
2865	3700	177	177	92	93	65	65	47	49	39	39
3282	3631	176	176	91	93	63	64	48	48	39	39
3596	3555	176	171	92	92	65	64	47	47	39	39

e) 20 minutes

865	775	152	152	87	92	66	61	46	46	42	42
3832	3577	180	177	90	88	61	61	47	46	41	41
1371	3610	182	181	95	95	66	64	46	49	41	39
1826	3564	176	178	93	94	63	65	48	48	38	39
1961	3539	175	177	92	92	64	64	46	49	40	38
2312	3665	179	175	92	91	65	66	47	49	40	39
2629	3586	178	177	92	92	64	64	48	50	40	40
2892	3123	174	171	91	93	65	64	48	48	39	39

f) 25 minutes

793	699	148	146	85	89	65	58	46	45	40	42
3744	3482	179	164	89	86	60	62	46	45	40	41
1185	3510	179	179	93	94	65	63	46	49	41	39
1443	3455	175	175	91	92	63	64	48	48	38	39
1615	3443	167	179	94	92	64	62	46	47	38	38
1885	3561	177	170	89	91	62	66	47	50	39	39
2127	3491	177	174	90	91	62	63	48	48	41	39
2342	2340	174	168	93	92	65	63	46	46	38	39

g) 30 minutes

765	681	149	149	85	90	68	61	45	46	42	41
3709	3447	178	159	89	87	62	63	47	46	42	40
1084	3493	182	183	95	95	66	63	46	50	39	38
1276	3443	177	178	90	95	64	65	48	48	37	39
1432	3427	167	177	94	94	63	64	46	48	38	39
1651	3558	171	168	89	92	65	65	47	50	40	39
1835	3479	178	176	90	93	63	64	48	50	41	39
2001	1973	175	170	92	92	65	64	48	48	38	39

Low fluorescence output — High fluorescence output

The results from this validation experiment verified that as long as measurements were taken within 5 minutes of removing the plate from the glovebox and the samples were not dispensed into the outer edge wells, the readings would not be affected by oxygen.

Fluorescence Quenching Experiment 1

Inside a nitrogen-filled glovebox, an iridium stock solution (Ir solution) was prepared by dissolving $[Ir(ppy)_2(dtbpy)]PF_6$ (6.0 mg) in acetonitrile (13.19 mL). A nickel/ligand solution (Ni&lig solution) was prepared by dissolving $NiCl_2$.glyme (64.1 mg) and 4,7-dimethoxy-1,10-phenanthroline (70.8 mg) in acetonitrile (11.31 mL). A bromocyclopropane solution (AlkBr solution) was prepared by dissolving bromocyclopropane (36.3 mg, 24.0 µL) in acetonitrile (11.86 mL). A bromoacetophenone solution (SM solution) was prepared by dissolving 4-bromoacetophenone (59.1 mg) in acetonitrile (11.82 mL). The stock solutions, as well as neat reagents, were dosed into 2 mL HPLC vials according to Table 28 and stirred for 15 minutes. The solution in well C6 did not turn the expected blue/green color, suggesting this solution had not successfully complexed. Each of these solutions (100 µL) were dosed into a 96-well fluorescence plate, in order to achieve the concentrations shown in Table 29. Each well of the plate was excited at 420 nm and emission was measured at 581 nm, using a SpectraMax M5 Multi-Mode Microplate Reader.

Table 28 Preparation of fluorescence samples from stock solutions of each reaction component.

	1	2	3	4	5	6
**A**	200 µL Ir solution<br>1800 µL MeCN	200 µL Ir solution<br>360 µL SM solution<br>1440 µL MeCN	200 µL Ir solution<br>720 µL SM solution<br>1080 µL MeCN	200 µL Ir solution<br>1080 µL SM solution<br>720 µL MeCN	200 µL Ir solution<br>1440 µL SM solution<br>360 µL MeCN	200 µL Ir solution<br>1800 µL SM solution
**B**	200 µL Ir solution<br>1800 µL MeCN	200 µL Ir solution<br>360 µL AlkBr solution<br>1440 µL MeCN	200 µL Ir solution<br>720 µL AlkBr solution<br>1080 µL MeCN	200 µL Ir solution<br>1080 µL AlkBr solution<br>720 µL MeCN	200 µL Ir solution<br>1440 µL AlkBr solution<br>360 µL MeCN	200 µL Ir solution<br>1800 µL AlkBr solution

**C**	200 µL Ir solu-tion<br>1800 µL MeCN	200 µL Ir solu-tion<br>360 µL Ni&lig solution<br>1440 µL MeCN	200 µL Ir solu-tion<br>720 µL Ni&lig solution<br>1080 µL MeCN	200 µL Ir solu-tion<br>1080 µL Ni&lig solution<br>720 µL MeCN	200 µL Ir solu-tion<br>1440 µL Ni&lig solution<br>360 µL MeCN	200 µL Ir solu-tion<br>1800 µL Ni&lig solution
**D**	200 µL Ir solu-tion<br>1800 µL MeCN	200 µL Ir solu-tion<br>2.9 µL SiOH<br>1800 µL MeCN	200 µL Ir solu-tion<br>5.8 µL SiOH<br>1800 µL MeCN	200 µL Ir solu-tion<br>8.8 µL SiOH<br>1800 µL MeCN	200 µL Ir solu-tion<br>11.7 µL SiOH<br>1800 µL MeCN	200 µL Ir solu-tion<br>14.6 µL SiOH<br>1800 µL MeCN
**E**	200 µL Ir solu-tion<br>1800 µL MeCN	200 µL Ir solu-tion<br>1 mg Na_2CO_3<br>1800 µL MeCN	200 µL Ir solu-tion<br>1.9 mg Na_2CO_3<br>1800 µL MeCN	200 µL Ir solu-tion<br>2.9 mg Na_2CO_3<br>1800 µL MeCN	200 µL Ir solu-tion<br>3.8 mg Na_2CO_3<br>1800 µL MeCN	200 µL Ir solu-tion<br>4.8 mg Na_2CO_3<br>1800 µL MeCN

Table 29 Fluorescence output values measured for [Ir(ppy)$_2$(dtbpy)]PF$_6$ ***(Ir 2)*** *in the presence of increasing concentrations of each reaction component.*

	Concentration (mM)					
	0	5	10	15	20	25
Bromoacetophenone **5**	462.4	458.7	455.7	441.9	441.7	445.1
Cyclopropyl bromide **A1**	526.0	444.5	452.0	442.9	454.5	441.9
Nickel & dOMe-phen	481.4	326.7	249.2	140.2	138.0	451.6
Supersilanol **60**	465.4	442.8	477.0	462.4	479.0	443.5
Sodium Carbonate	475.7	473.4	451.7	458.7	465.9	451.5

Low fluorescence output — High fluorescence output

It was noted that the 25 mM nickel & ligand solution did not dissolve, and therefore complex, effectively, despite extensive mixing. This solution appears to have not quenched the **Ir 2** excited state, which suggests that the nickel source and ligand cannot be effective quenchers as separate entities and must be complexed.

Fluorescence Quenching Experiment 3

Inside a nitrogen-filled glovebox, an iridium stock solution (Ir solution) was prepared by dissolving [Ir(ppy)$_2$(dtbpy)]PF_6 (16.5 mg) in acetonitrile (8.98 mL). A nickel/ligand solution (Ni&lig solution) was prepared by dissolving $NiCl_2$.glyme (15.8 mg) and 4,7-dimethoxy-1,10-phenanthroline (17.5 mg) in acetonitrile (12.67 mL). The stock solutions were prepared as stated in Table 30 and dosed into prepared directly into a cuvette, in order to achieve the concentrations shown in Figure 10. Each well of the plate was excited at 420 nm and emission was measured at 581 nm, using a SpectraMax M5 Multi-Mode Microplate Reader. A Stern-Volmer plot was generated from these results (Figure 11).

Table 30 Preparation of fluorescence samples in cuvettes, from stock solutions of iridium, nickel and ligand.

	**1**	**2**	**3**	**4**	**5**	**6**
A	100 µL Ir solution<br>900 µL MeCN	100 µL Ir solution<br>37.8 µL Ni&lig solution<br>862.2 µL MeCN	100 µL Ir solution<br>75.8 µL Ni&lig solution<br>824.2 µL MeCN	100 µL Ir solution<br>113.7 µL Ni&lig solution<br>786.3 µL MeCN	100 µL Ir solution<br>151.5 µL Ni&lig solution<br>748.5 µL MeCN	100 µL Ir solution<br>189.3 µL Ni&lig solution<br>710.7 µL MeCN
B	100 µL Ir solution<br>227.3 µL Ni&lig solution<br>672.7 µL MeCN	100 µL Ir solution<br>303.0 µL Ni&lig solution<br>597.0 µL MeCN				

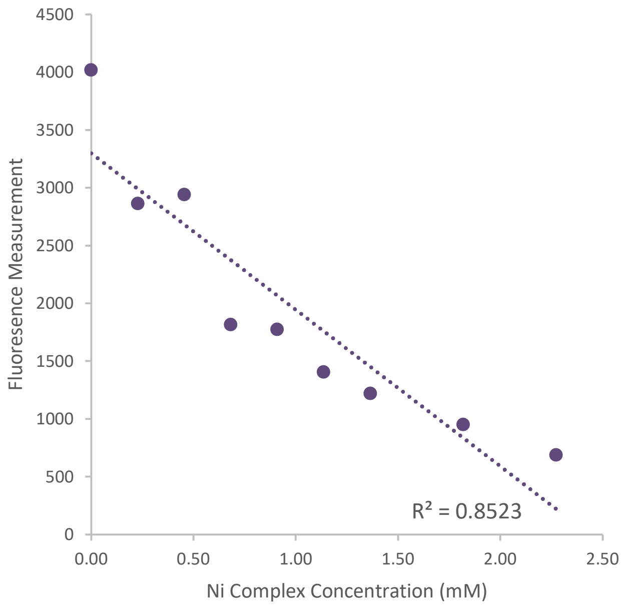

*Figure 10 Fluorescence output values measured for **Ir 2** in the presence of increasing concentrations of the nickel/ligand complex, from 0 to 2.3 mM.*

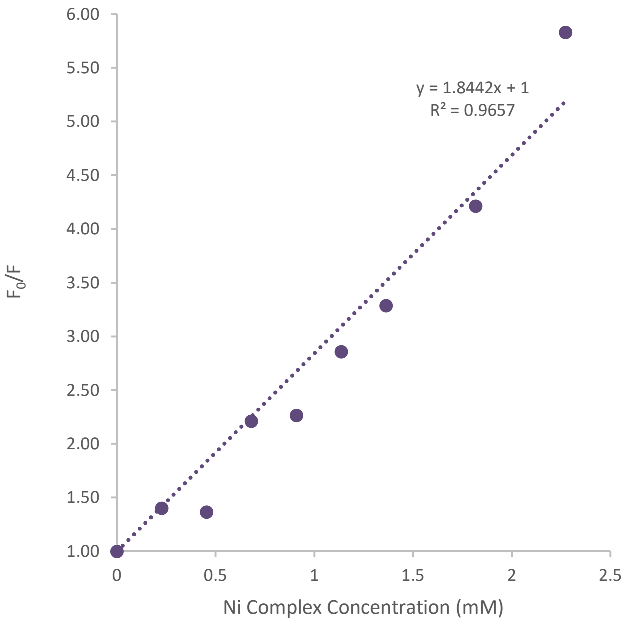

*Figure 11 Stern-Volmer plot for the quenching of **Ir 2*** at increasing concentrations of nickel/ligand complex.*

The Stern-Volmer constant, KSV, was determined from Figure 11 to be 1844 M^{-1}. The excited state life-time of **Ir 2*** has been reported at 0.57 μs,[16] which allows the calculation of the quenching constant, k_q (Equation (1)).

$$k_q = \frac{K_{SV}}{\tau_0} = \frac{1844}{0.00000057} = 3.24 \times 10^9\ M^{-1}s^{-1} \qquad (1)$$

Fluorescence Quenching Experiment 4

Inside a nitrogen-filled glovebox, an iridium stock solution (Ir solution) was prepared by dissolving $[Ir(ppy)_2(dtbpy)]PF_6$ (1.4 mg) in acetonitrile (7.92 mL). The stock solution was dosed into 2 mL HPLC vials containing increasing amounts of LiBr, according to Table 31, and stirred before each of these solutions (100 μL) were dosed into a 96-well fluorescence plate, in order to achieve the concentrations shown in the Stern-Volmer plot, Figure 12. Each well of the plate was excited at 420 nm and emission was measured at 581 nm, using a SpectraMax M5 Multi-Mode Microplate Reader.

Table 31 Preparation of fluorescence samples from LiBr and a stock solution of $[Ir(ppy)_2(dtbpy)]PF_6$.

	**1**	**2**	**3**	**4**	**5**	**6**
A	500 μL Ir solution	500 μL Ir solution 10.0 mg LiBr	500 μL Ir solution 19.5 mg LiBr	500 μL Ir solution 29.5 mg LiBr	500 μL Ir solution 39.5 mg LiBr	500 μL Ir solution 49.5 mg LiBr
B	500 μL Ir solution 59.1 mg LiBr	500 μL Ir solution 79.1 mg LiBr	500 μL Ir solution 98.6 mg LiBr			

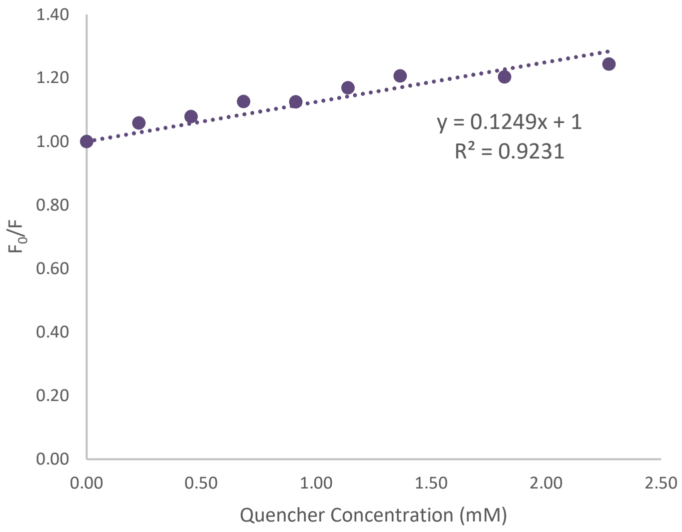

*Figure 12 Stern-Volmer plot for the quenching of **Ir 2*** at increasing concentrations of LiBr.*

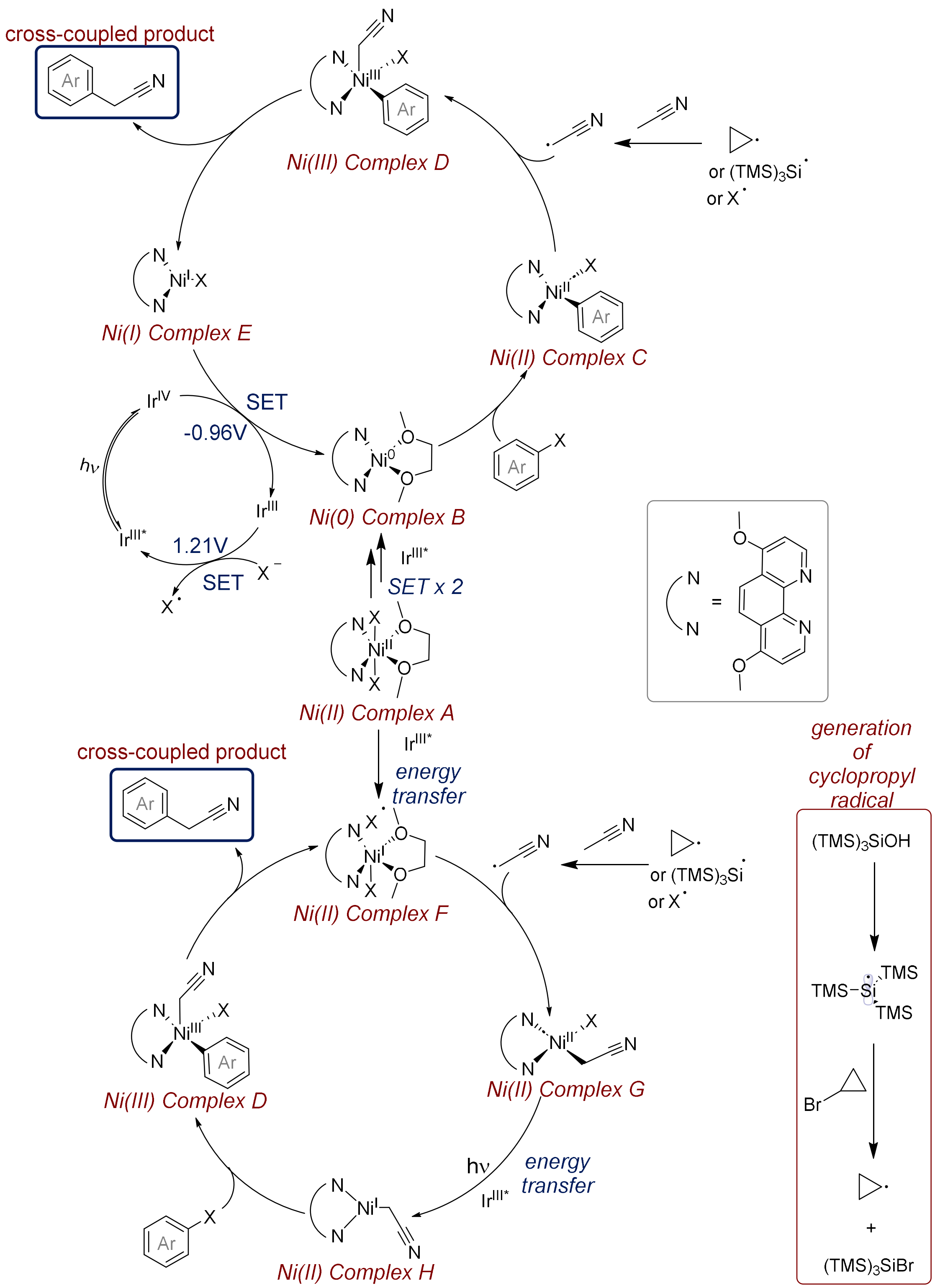

Scheme 2 Mechanistic hypothesis for the photoredox cyanomethylation reaction.

An electron transfer mechanism was initially proposed based on consideration of the progenitor metal-laphotoredox cross-electrophile coupling.[17] To this end, we have suggested a potential cycle, whereby

upon irradiation, the Ir(III) photocatalyst reaches its excited state, Ir(III)*. In this state, it has an oxidation potential of -0.96 V; it is reasoned that this could be sufficient to reduce Ni(II) Complex A to Ni(I), and Ni(I) to Ni(0), through two sequential SET events.[18] Once the catalytically active Ni(0) Complex B is formed, oxidative addition of the aryl halide would lead to Ni(II) Complex C. Addition of the cyanomethyl radical into Ni(II) Complex C, leads to Ni(III) Complex D. This complex is able to reductively eliminate to give the cross coupled product, and Ni(I) Complex E, which could be subsequently reduced back to Ni(0) Complex B by Ir(III)*. Finally, it is proposed that the resultant Ir(IV) species may be reduced back to Ir(III) by a halide anion, simultaneously generating a halogen radical, which could participate in further activation of supersilanol.

Subsequent mechanistic studies into metallaphotoredox cross-electrophile coupling reactions have suggested they may occur via an energy transfer process.[19, 20] Against this background, we hypothesize that upon energy transfer from the Ir(III) photosensitizer to the Ni(II) Complex A, a halogen radical would be photoeliminated. Combination of the cyanomethyl radical with the resulting Ni(I) Complex F would create the Ni(II) Complex G. A second energy transfer step could promote loss of another halogen radical, to form Ni(I) Complex H, followed by oxidation addition of the aryl halide to generate Ni(III) Complex D. Reductive elimination would result in the cross-coupled product and Ni(I) Complex F.

Although the findings of the current study do not unambiguously confirm either of these mechanistic proposals, the experimental findings, allied with literature precedent for metallaphotoredox couplings, have allowed us to formulate a working hypothesis. Furthermore, it is anticipated that there may be multiple reaction pathways occurring simultaneously, and this will be the focus of future investigations.

6. NMR Spectra

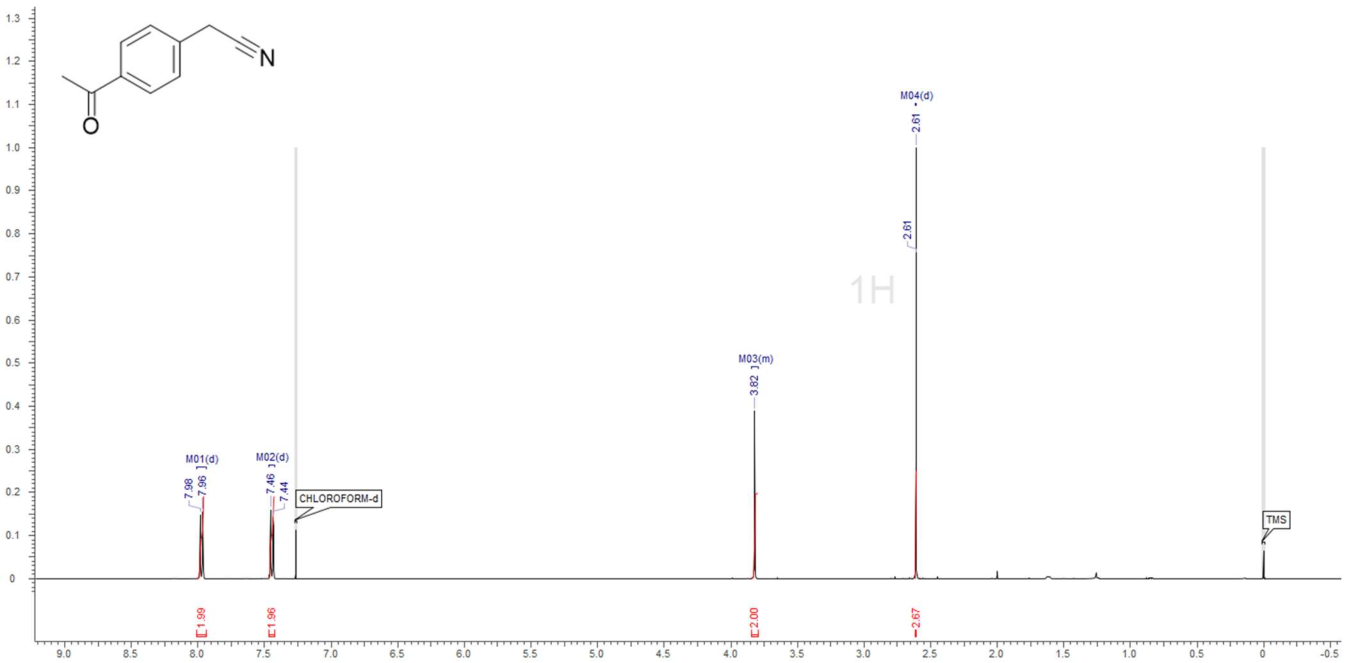

*Figure 13 ^{1}H NMR spectrum (400 MHz, $CDCl_3$) of compound **4**.*

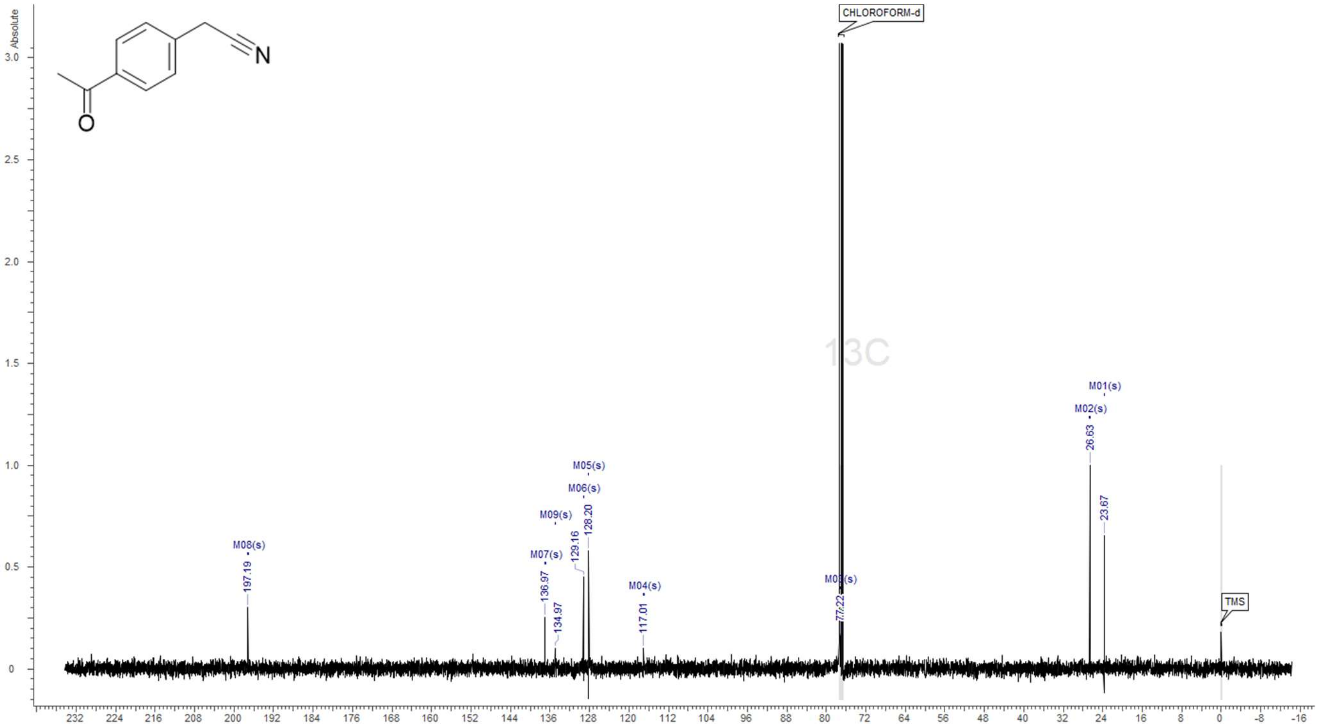

*Figure 14 ^{13}C NMR spectrum (101 MHz, $CDCl_3$) of compound **4**.*

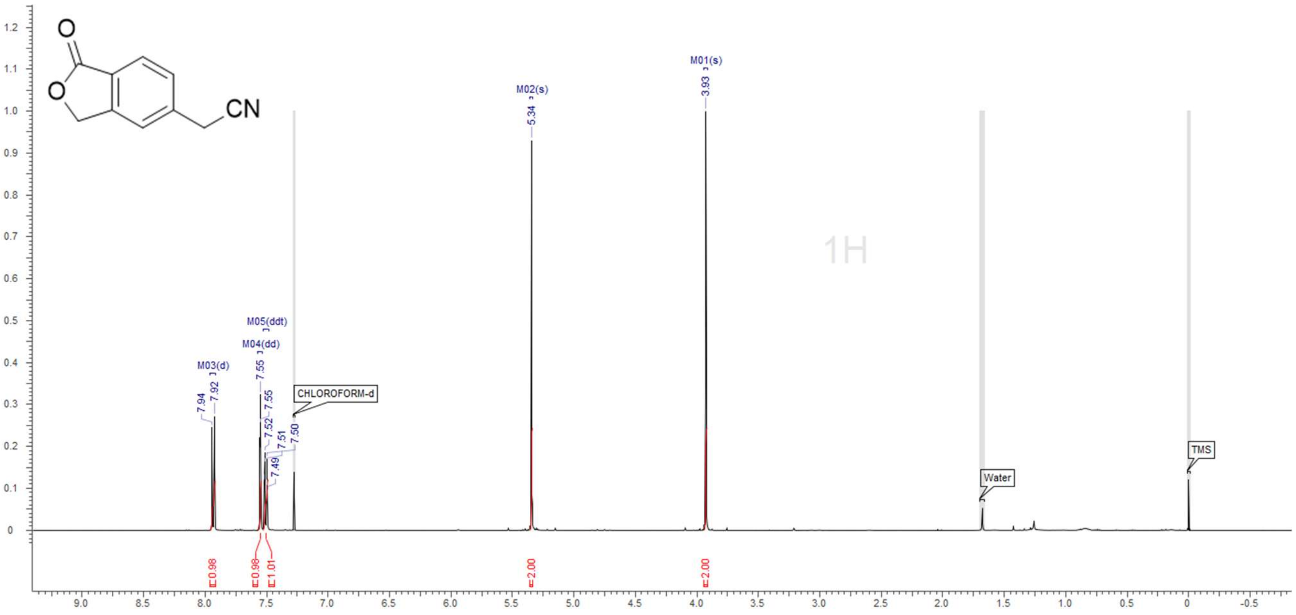

Figure 15 [1]*H NMR spectrum (400 MHz, CDCl*$_3$*) of compound* ***6***.

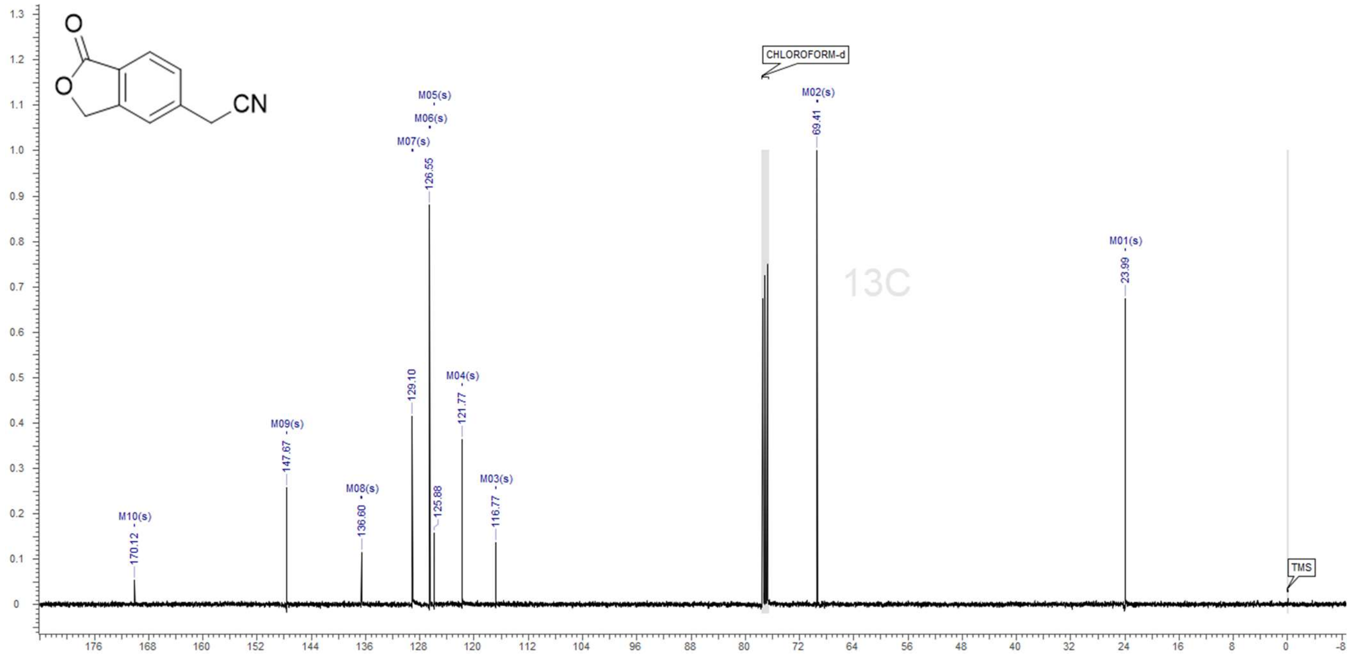

Figure 16 [13]*C NMR spectrum (101 MHz, CDCl*$_3$*) of compound* ***6***.

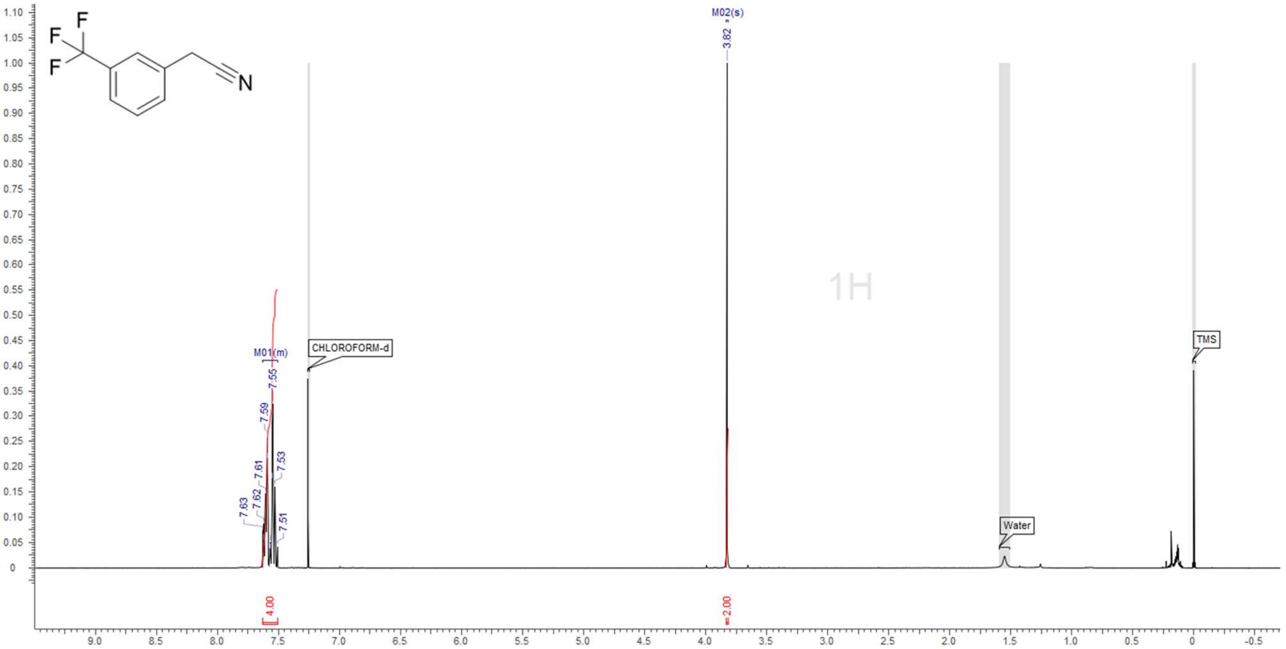

*Figure 17 ^{1}H NMR spectrum (400 MHz, $CDCl_3$) of compound **7**.*

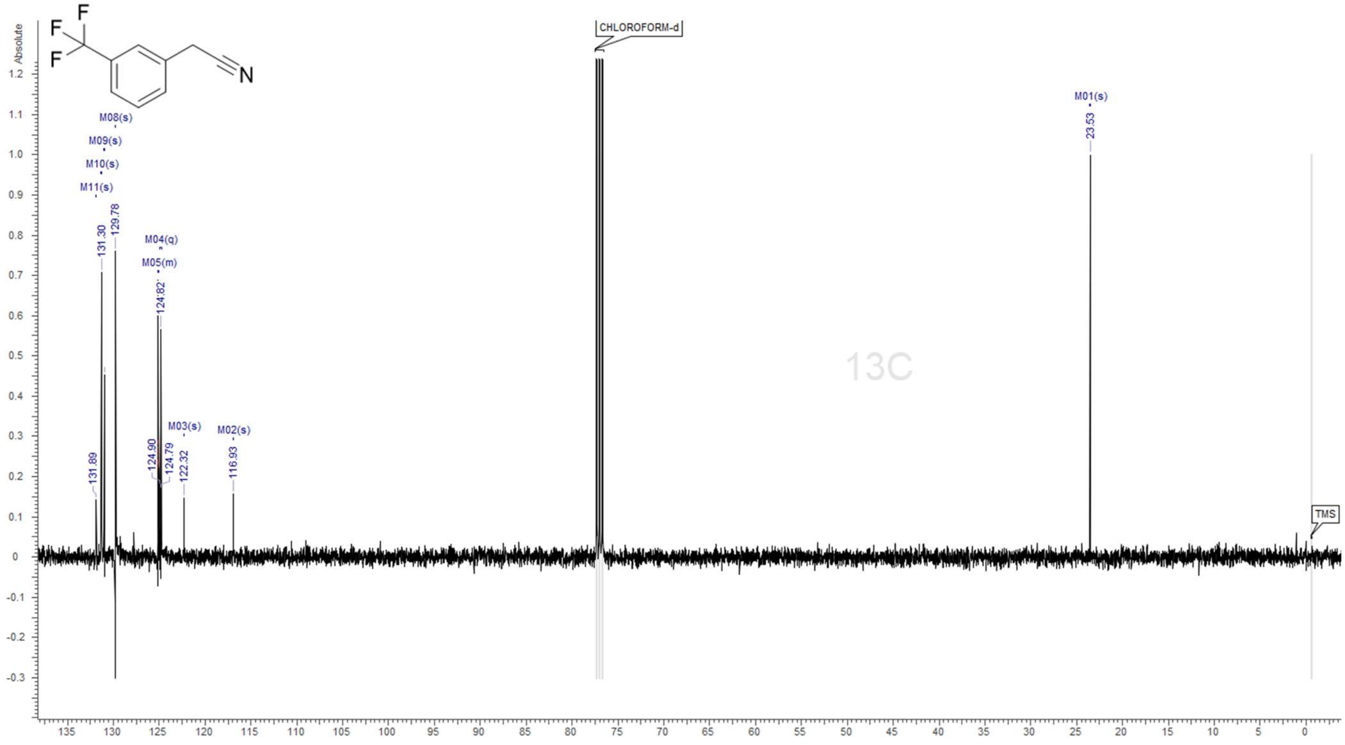

*Figure 18 ^{13}C NMR spectrum (101 MHz, $CDCl_3$) of compound **7**.*

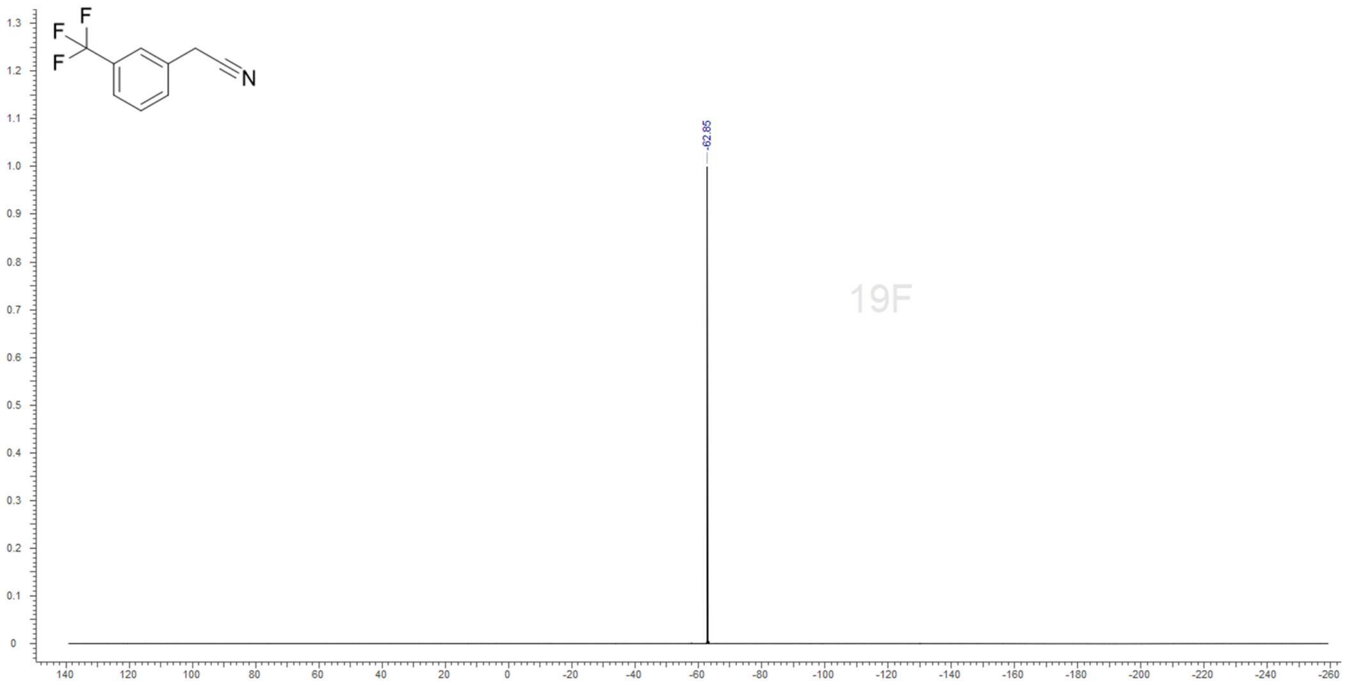

Figure 19 [19]*F NMR spectrum (376 MHz, $CDCl_3$) of compound* ***7***.

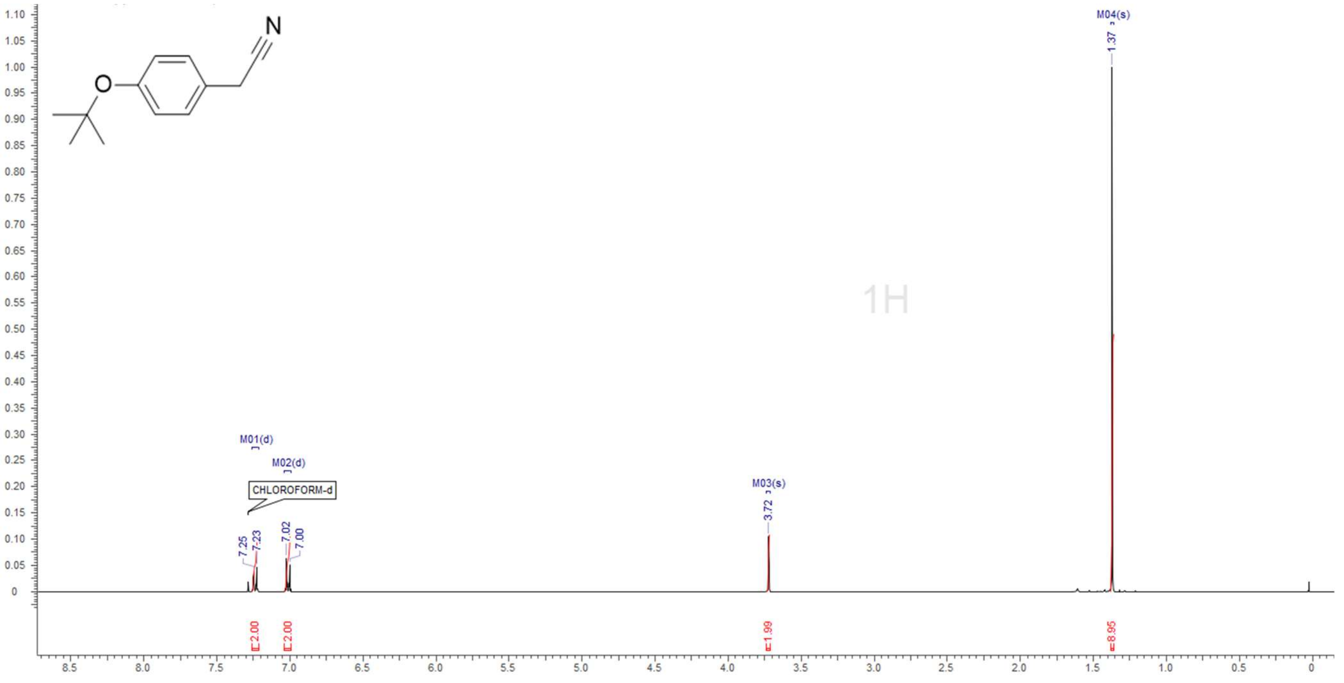

Figure 20 [1]*H NMR spectrum (400 MHz, $CDCl_3$) of compound* ***8***.

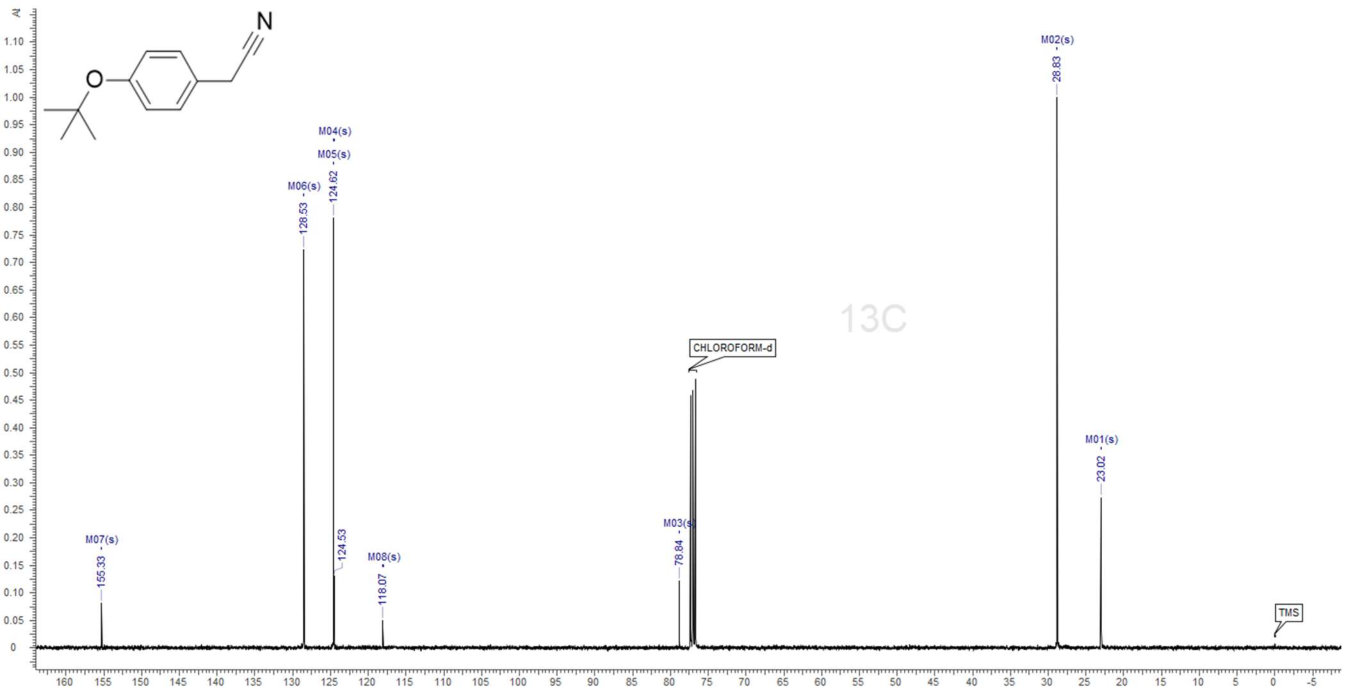

*Figure 21 ^{13}C NMR spectrum (101 MHz, $CDCl_3$) of compound **8**.*

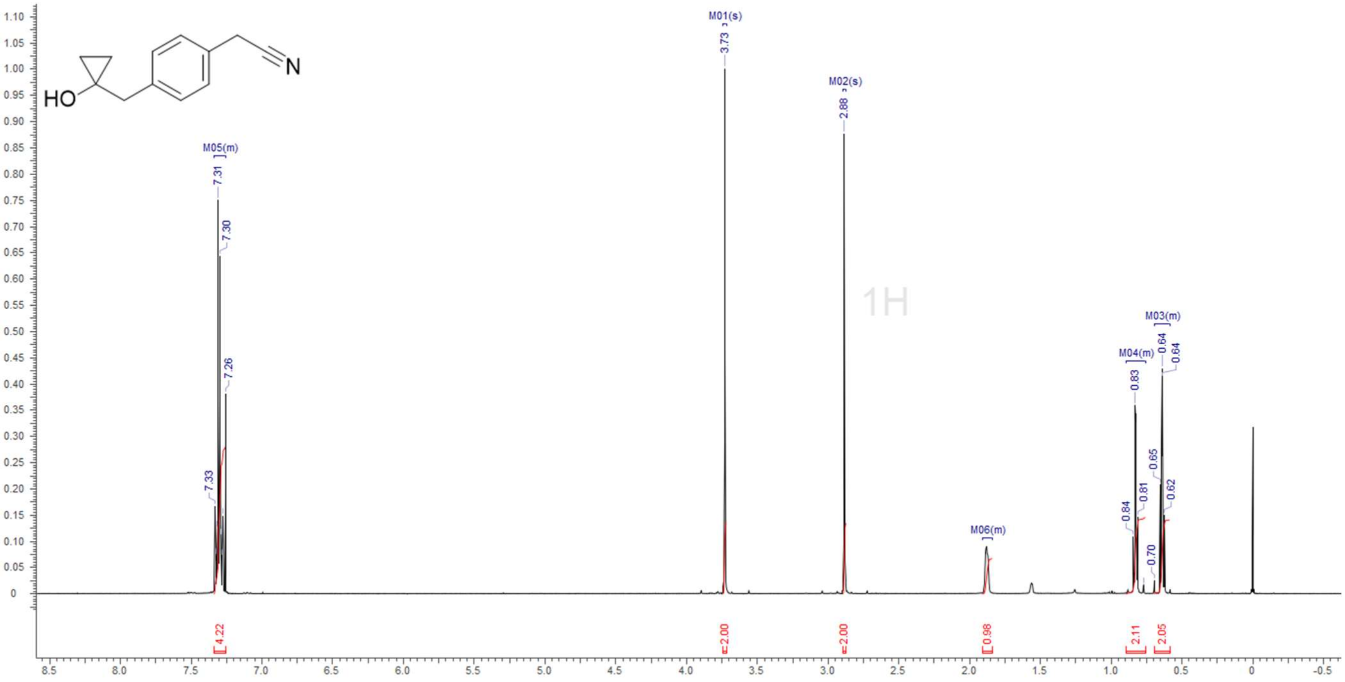

*^{1}H NMR spectrum (400 MHz, $CDCl_3$) of compound **9**.*

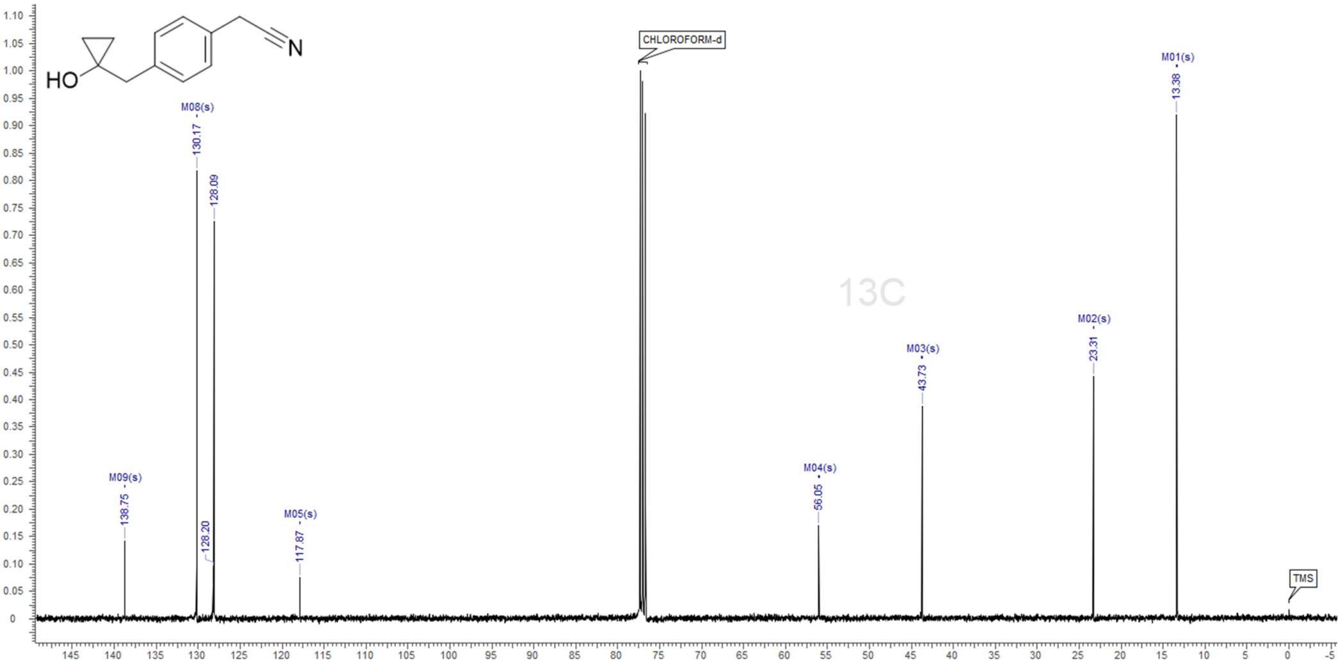

*Figure 22 ^{13}C NMR spectrum (101 MHz, $CDCl_3$) of compound **9**.*

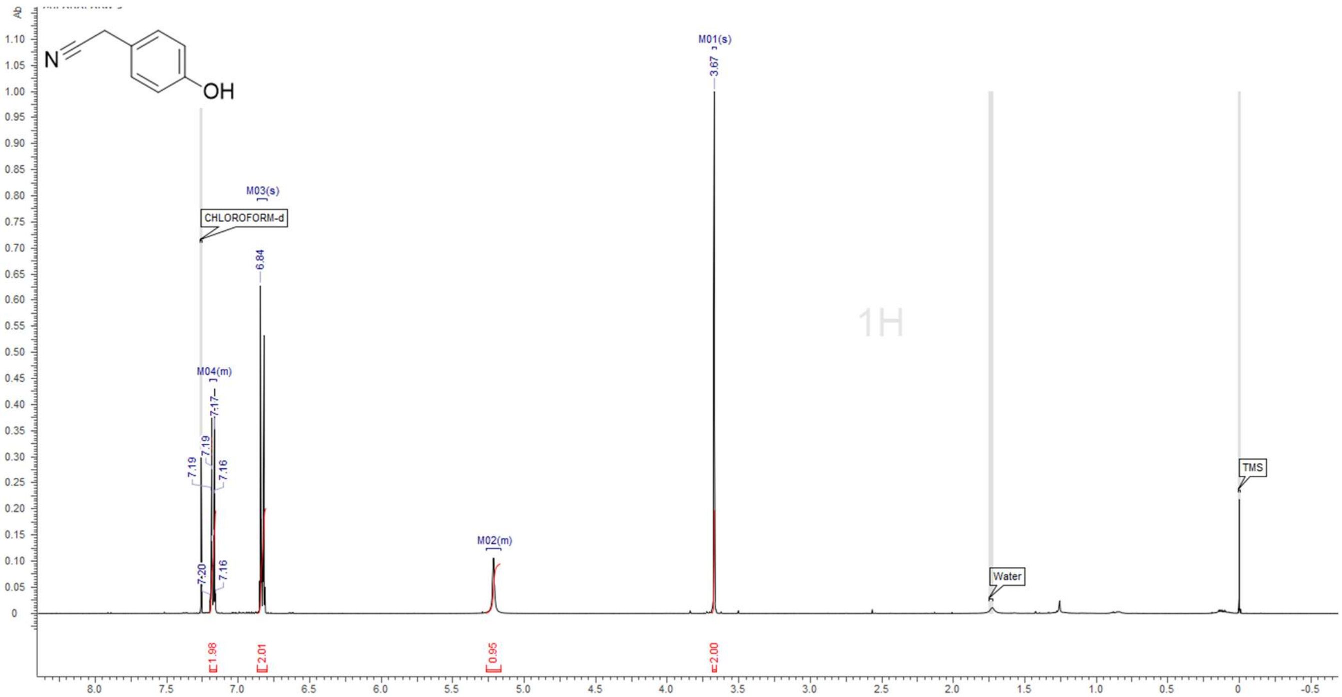

*Figure 23 ^{1}H NMR spectrum (400 MHz, $CDCl_3$) of compound **10**.*

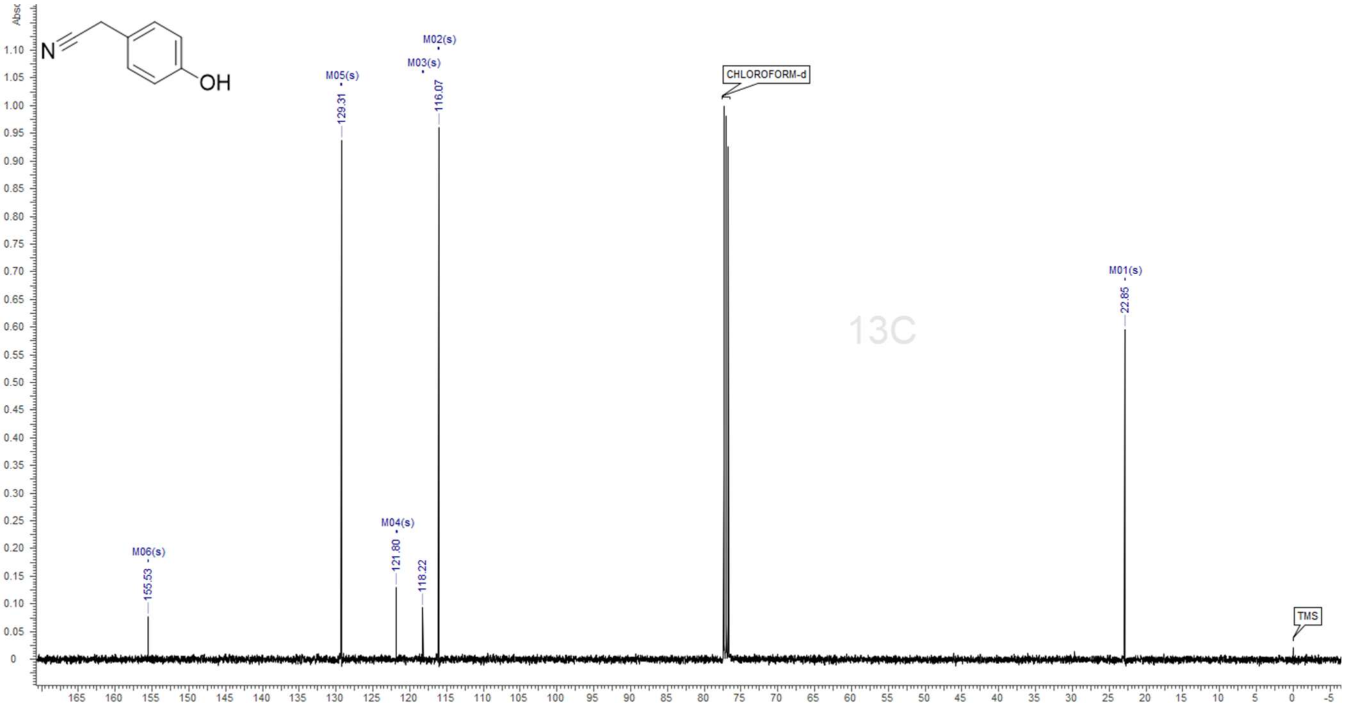

*Figure 24 ^{13}C NMR spectrum (101 MHz, $CDCl_3$) of compound **10**.*

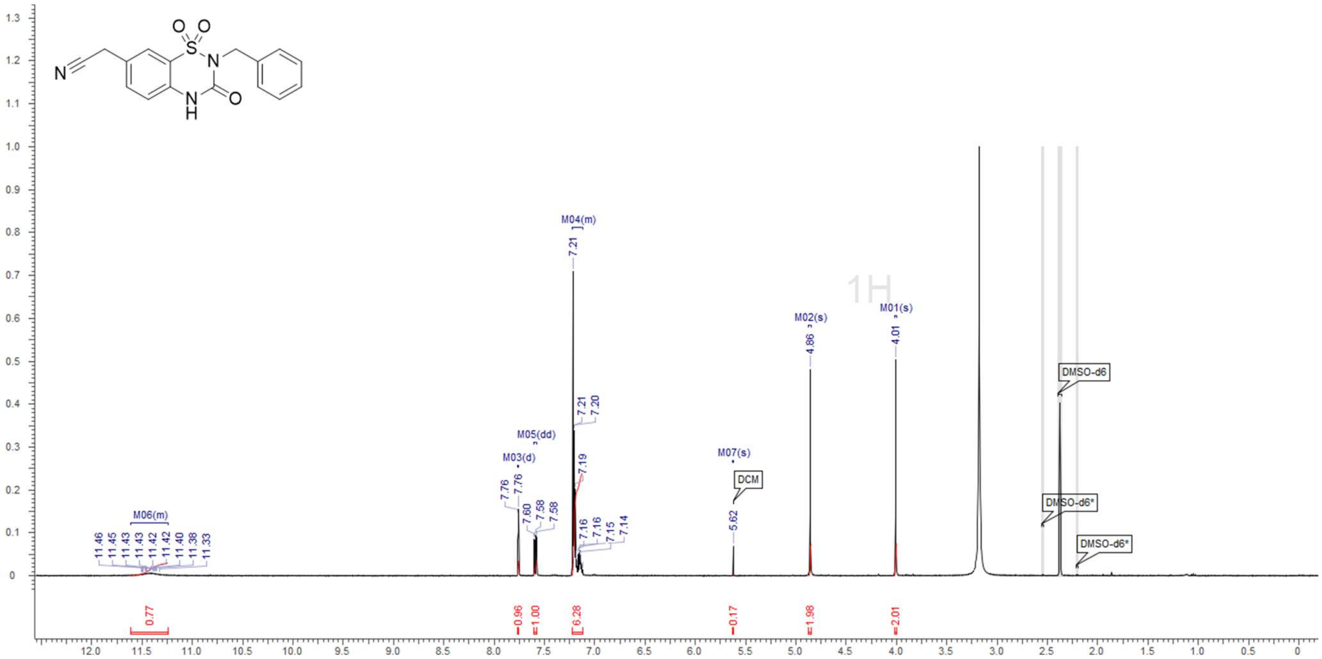

*Figure 25 ^{1}H NMR spectrum (400 MHz, DMSO-d$_6$) of compound **3**.*

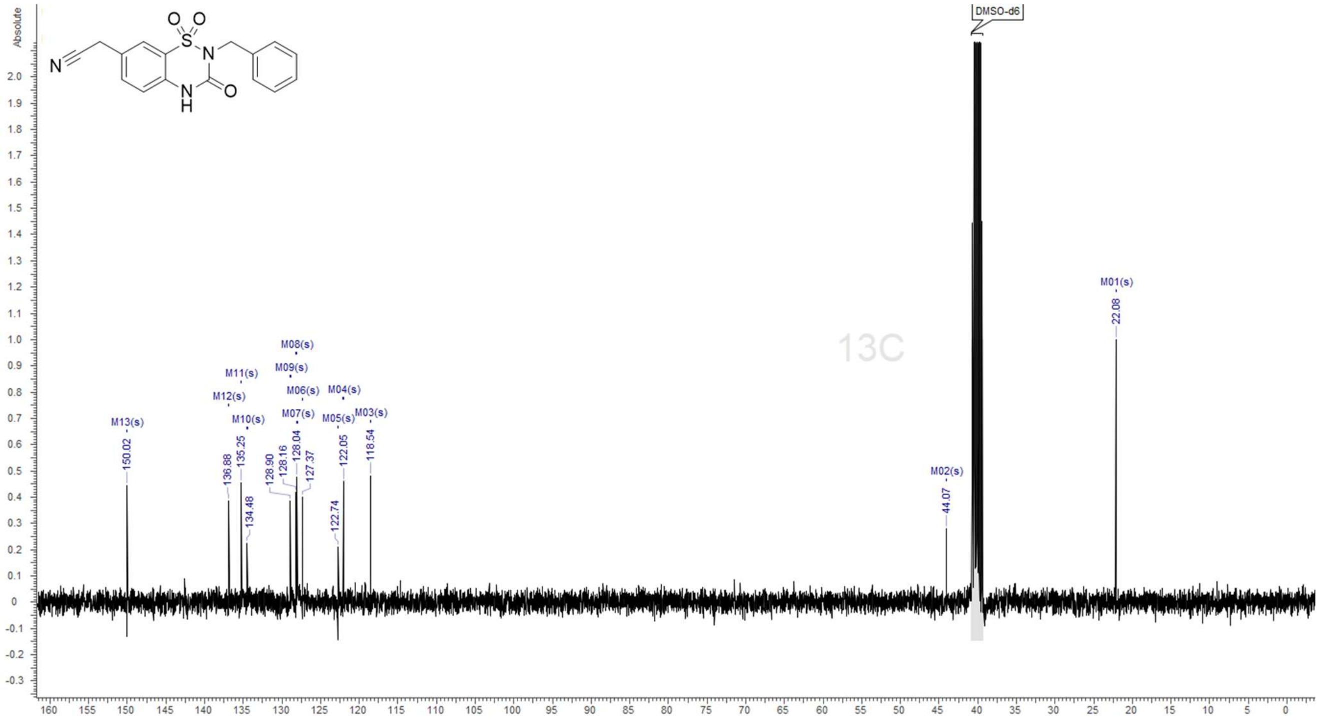

*Figure 26 ^{13}C NMR spectrum (101 MHz, DMSO-*d*₆) of compound **3**.*

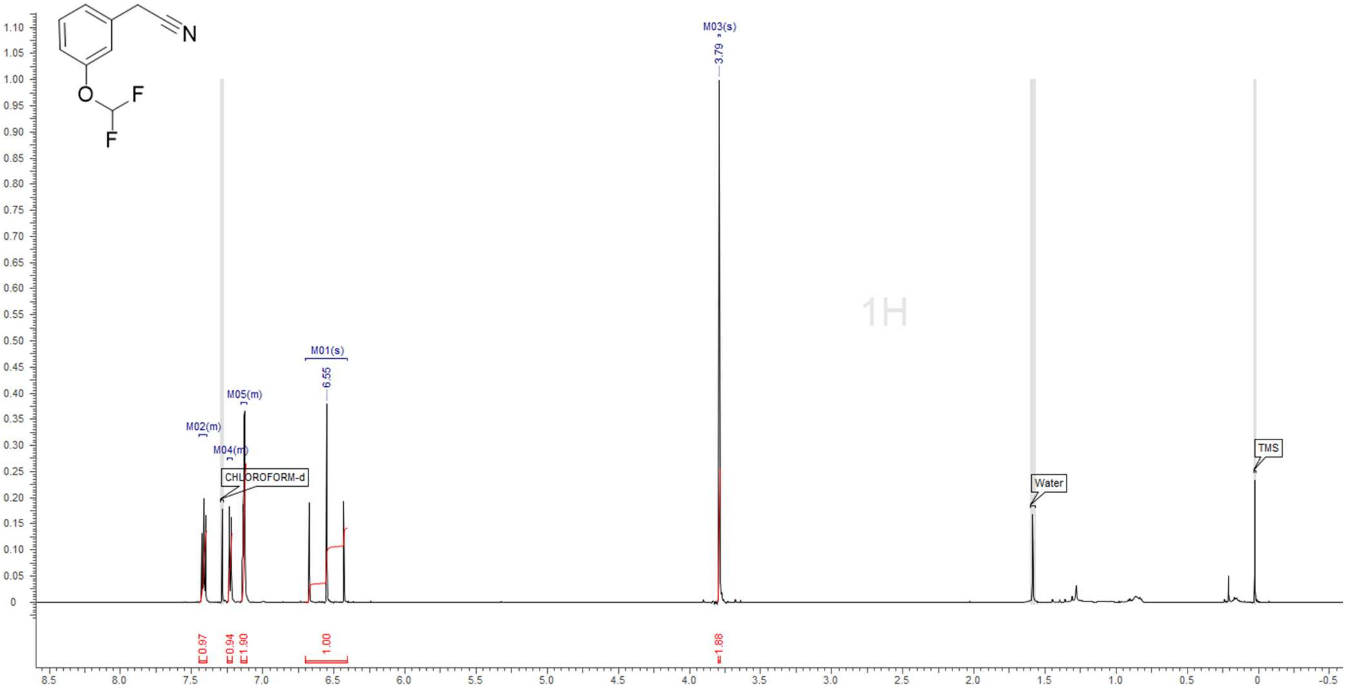

*Figure 27 ^{1}H NMR spectrum (400 MHz, $CDCl_3$) of compound **11**.*

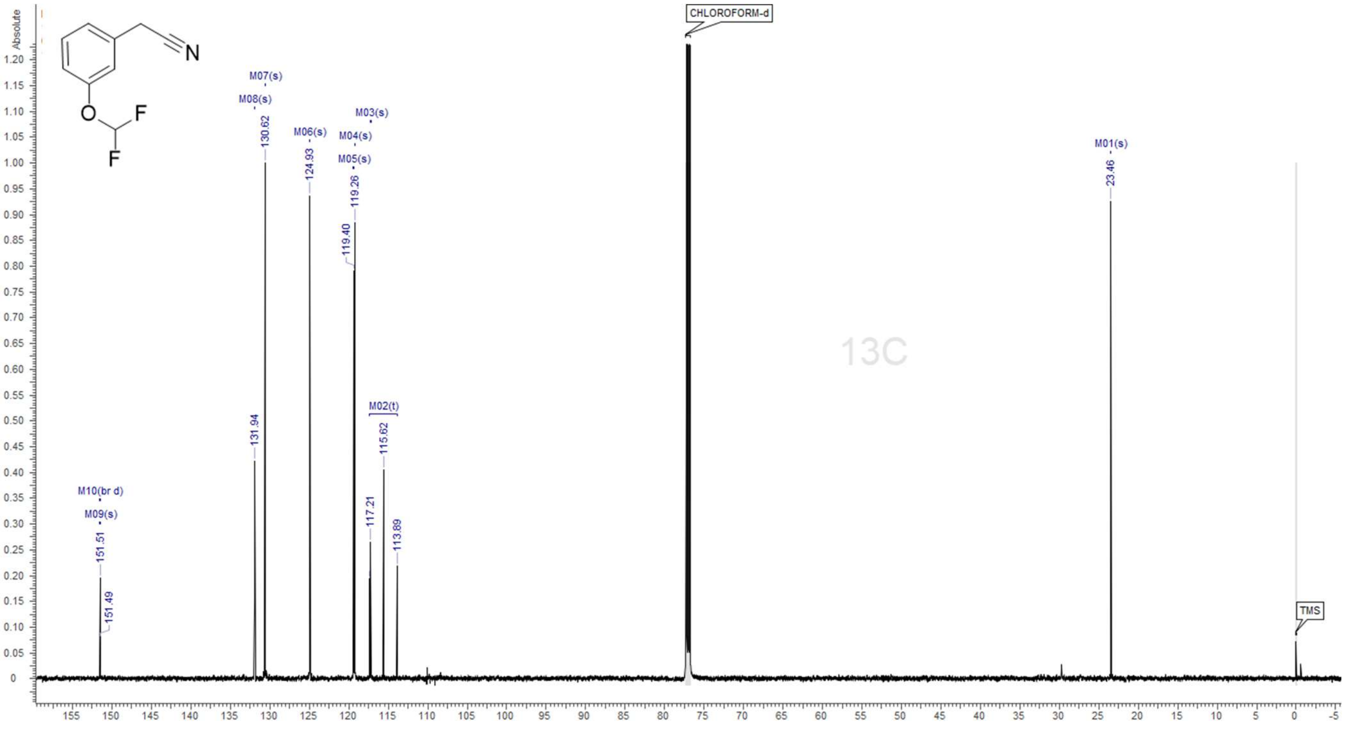

*Figure 28 ^{13}C NMR spectrum (101 MHz, $CDCl_3$) of compound **11**.*

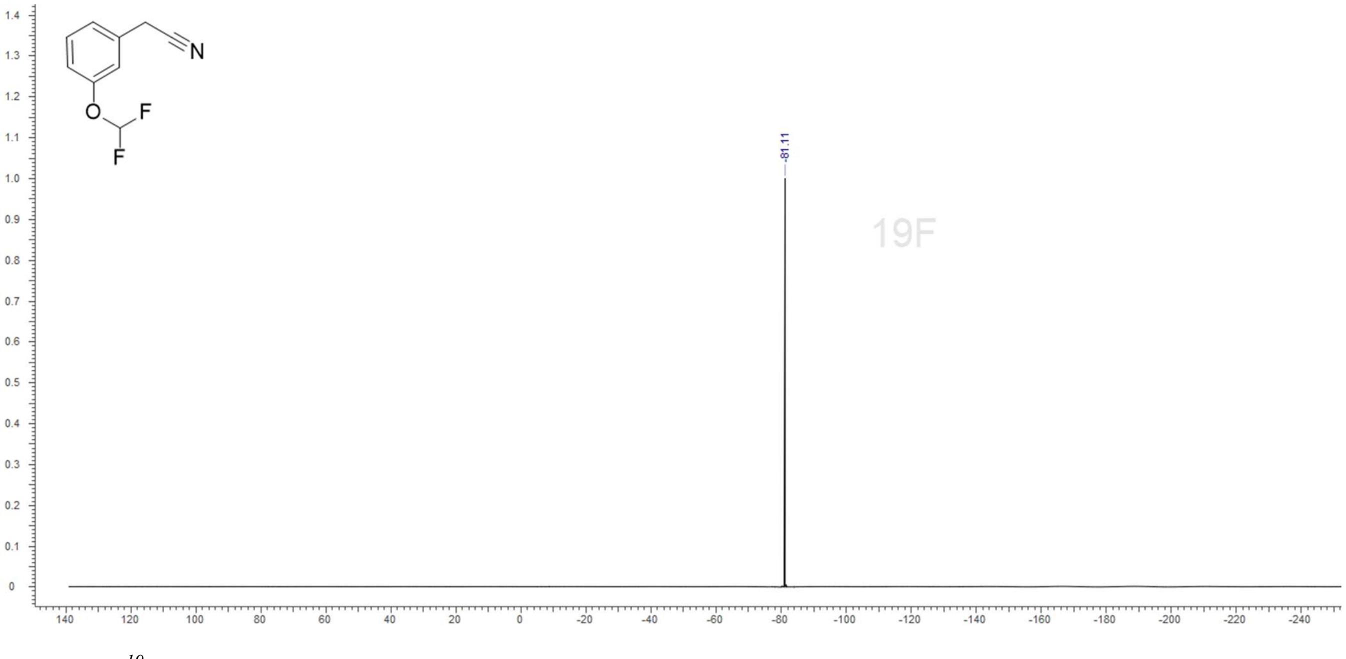

*Figure 29 ^{19}F NMR spectrum (376 MHz, $CDCl_3$) of compound **11**.*

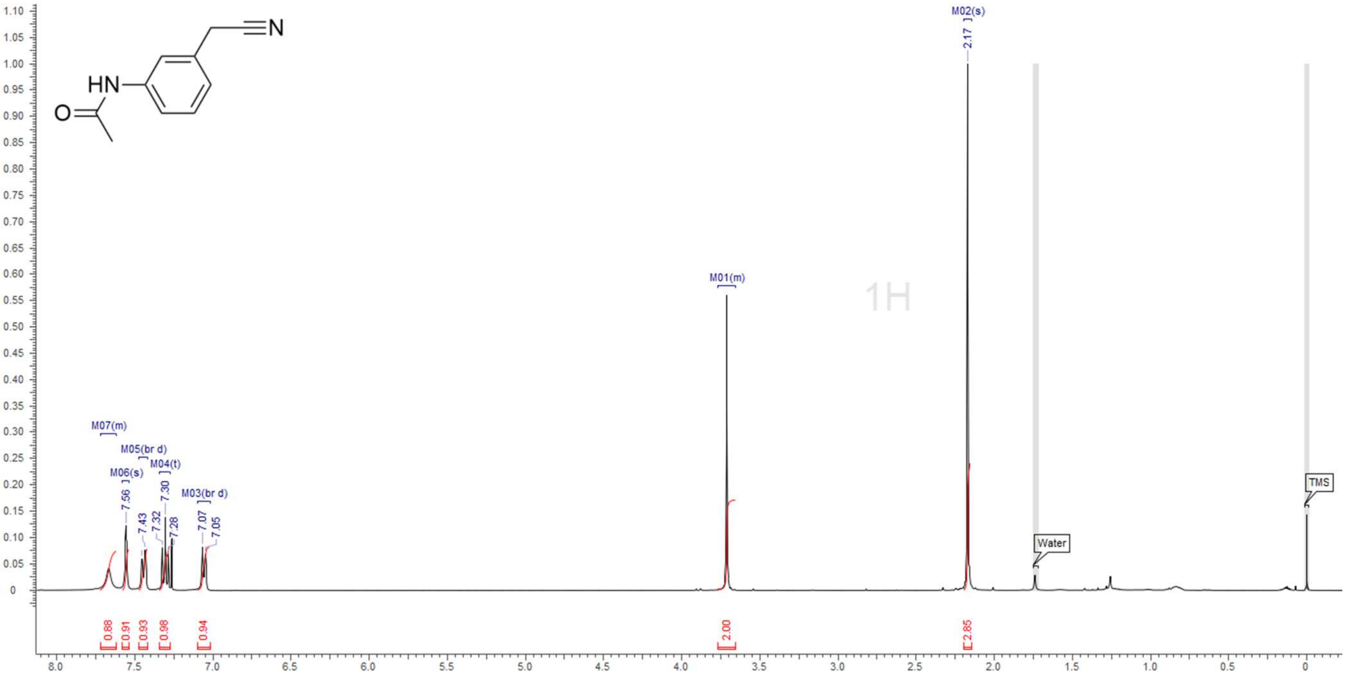

*Figure 30 ^{1}H NMR spectrum (400 MHz, CDCl$_3$) of compound **12**.*

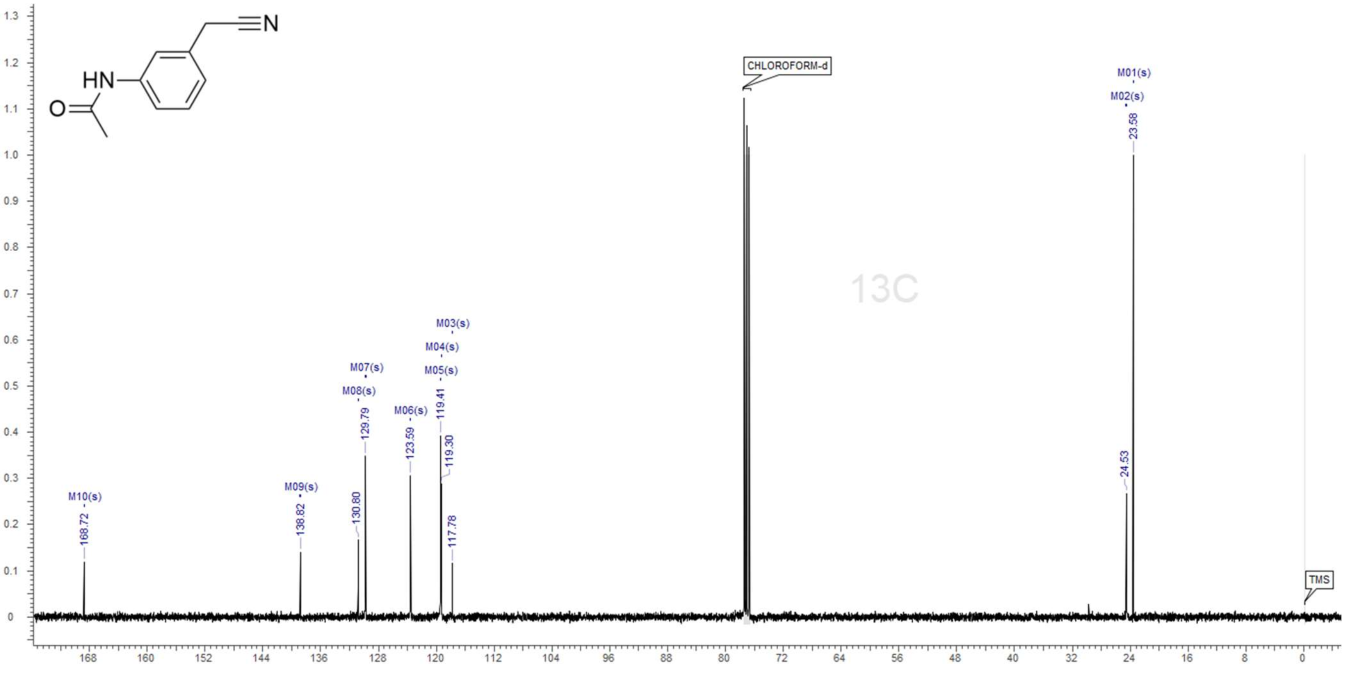

*Figure 31 ^{13}C NMR spectrum (101 MHz, CDCl$_3$) of compound **12**.*

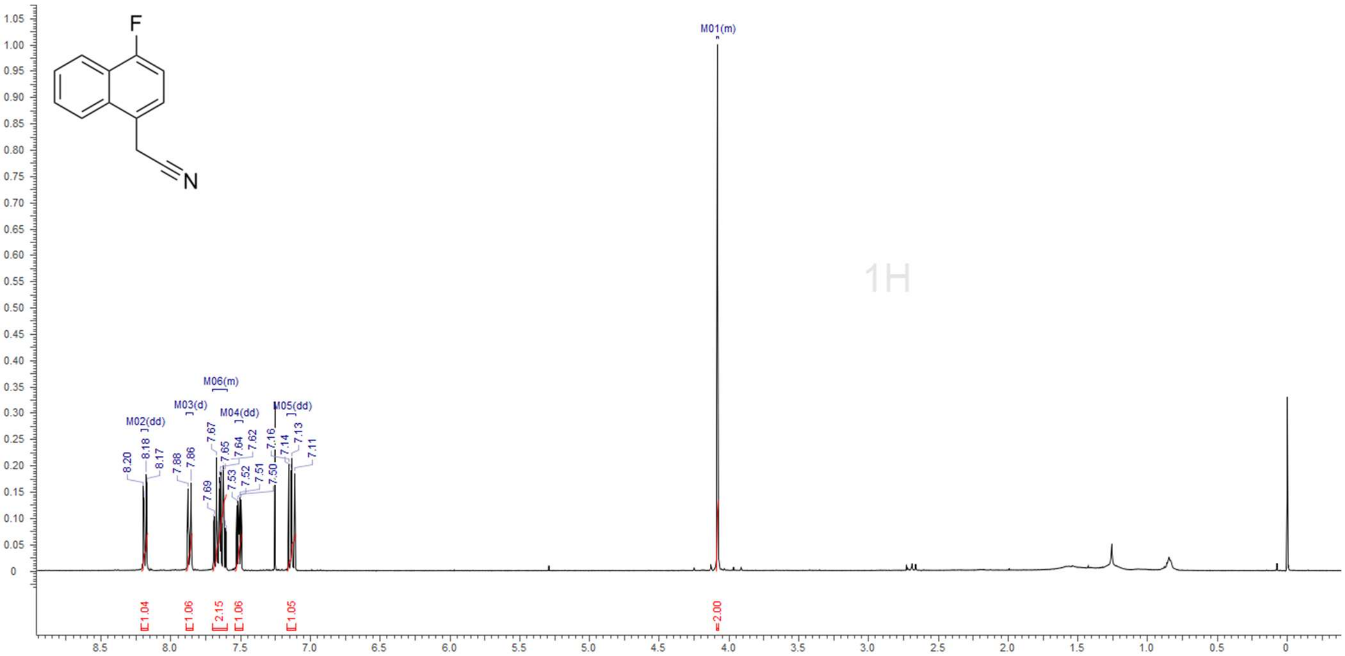

Figure 32 ^{1}H NMR spectrum (400 MHz, $CDCl_3$) of compound **13**.

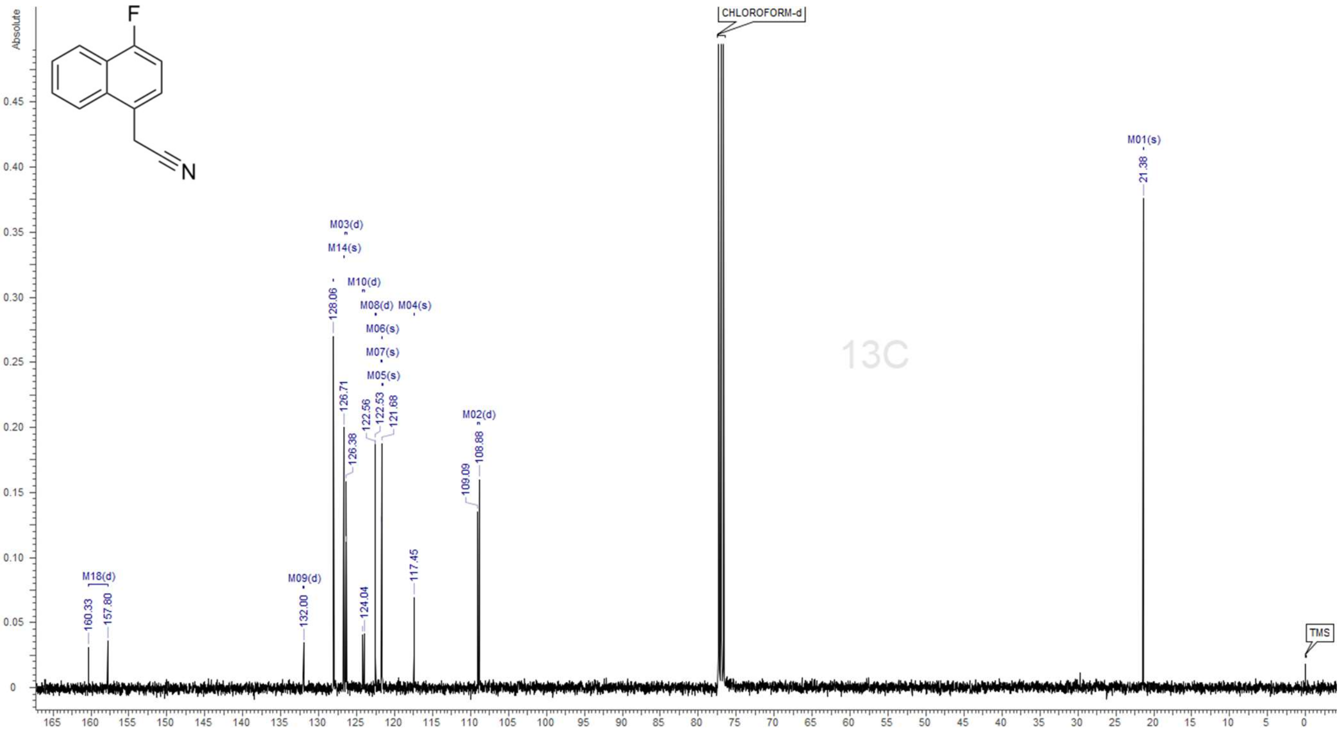

Figure 33 ^{13}C NMR spectrum (101 MHz, $CDCl_3$) of compound **13**.

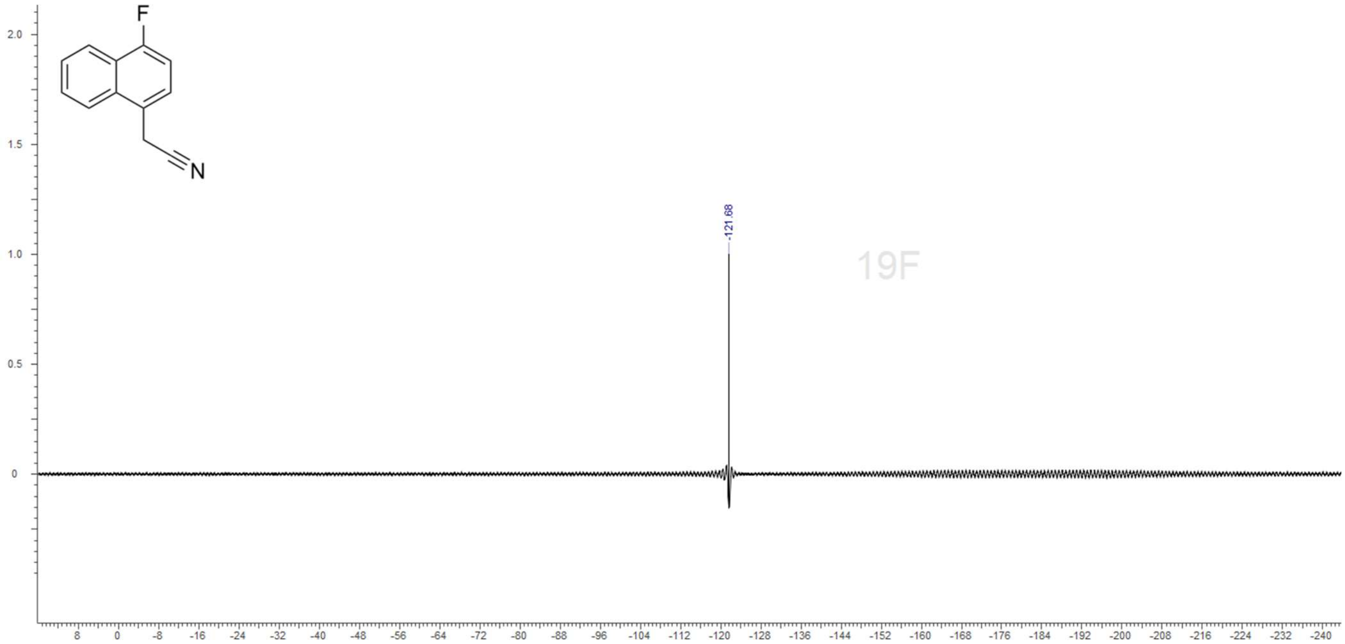

Figure 34 [19]*F NMR spectrum (376 MHz, $CDCl_3$) of compound* ***13***.

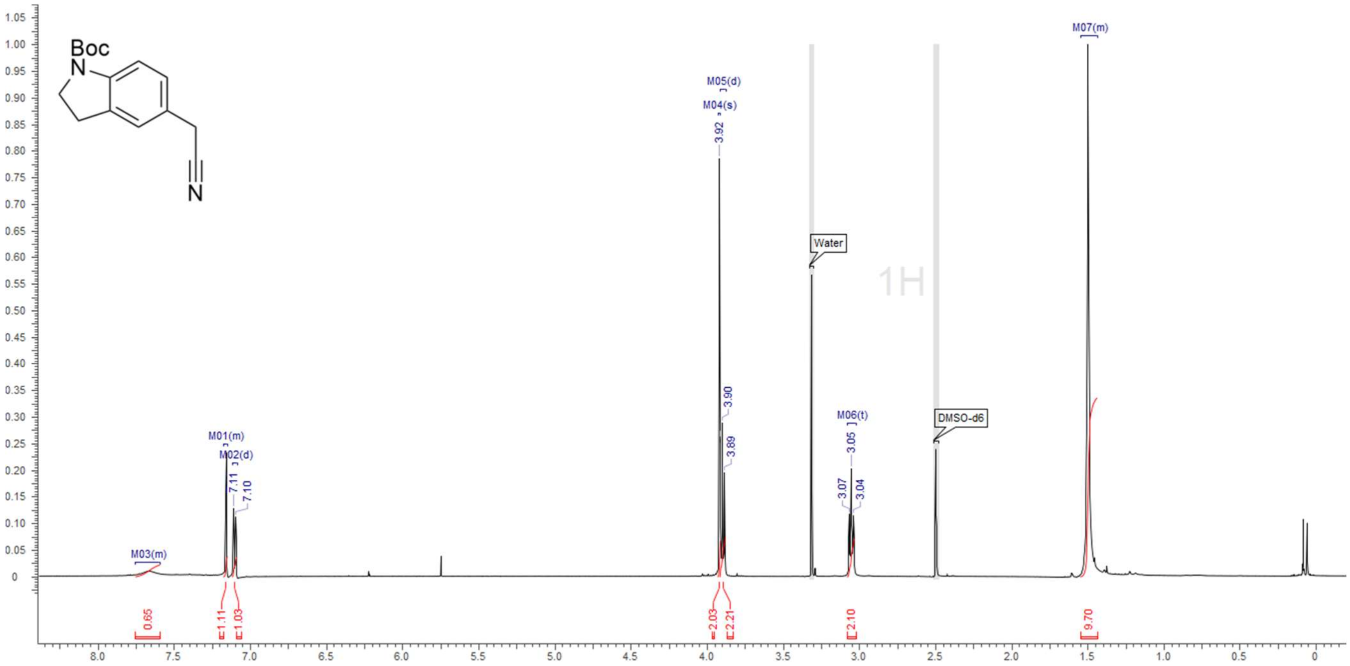

Figure 35 [1]*H NMR spectrum (600 MHz, DMSO-*d$_6$*, 298 K) of compound* ***14***.

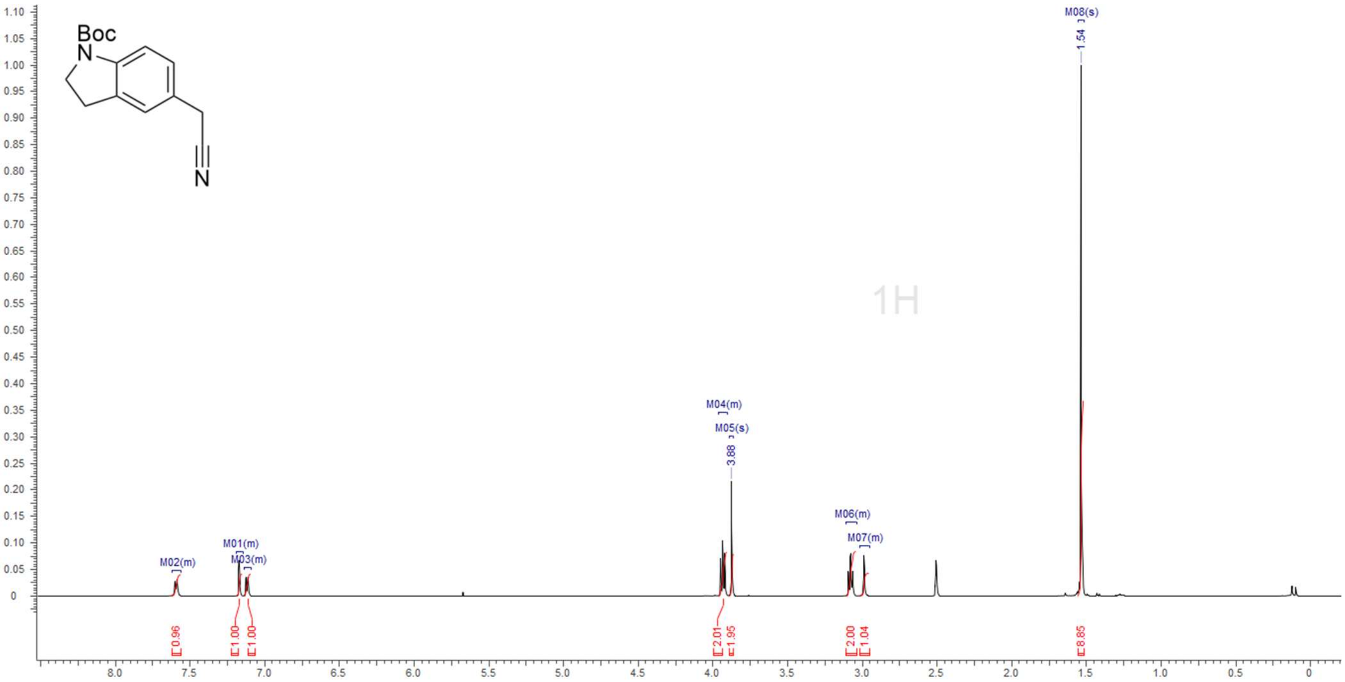

*Figure 36 ^{1}H NMR spectrum (600 MHz, DMSO-*d*$_6$, 373 K) of compound* ***14***.

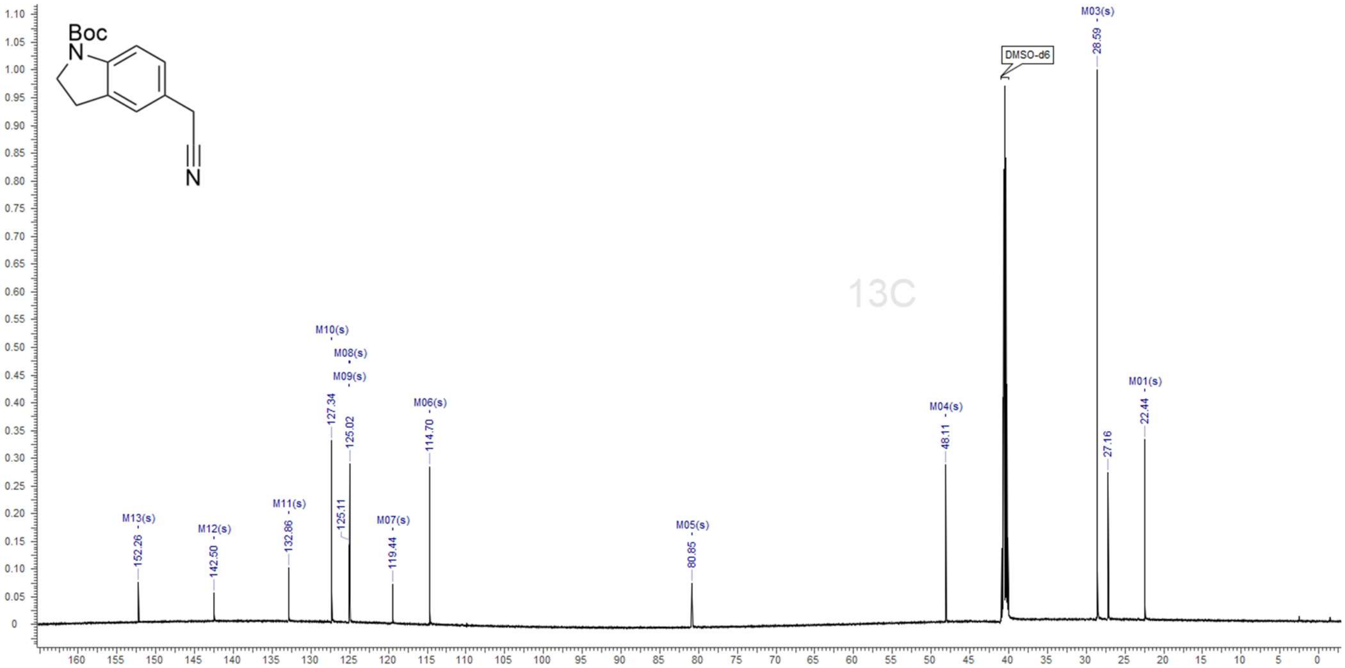

*Figure 37 ^{13}C NMR spectrum (101 MHz, DMSO-*d*$_6$) of compound* ***14***.

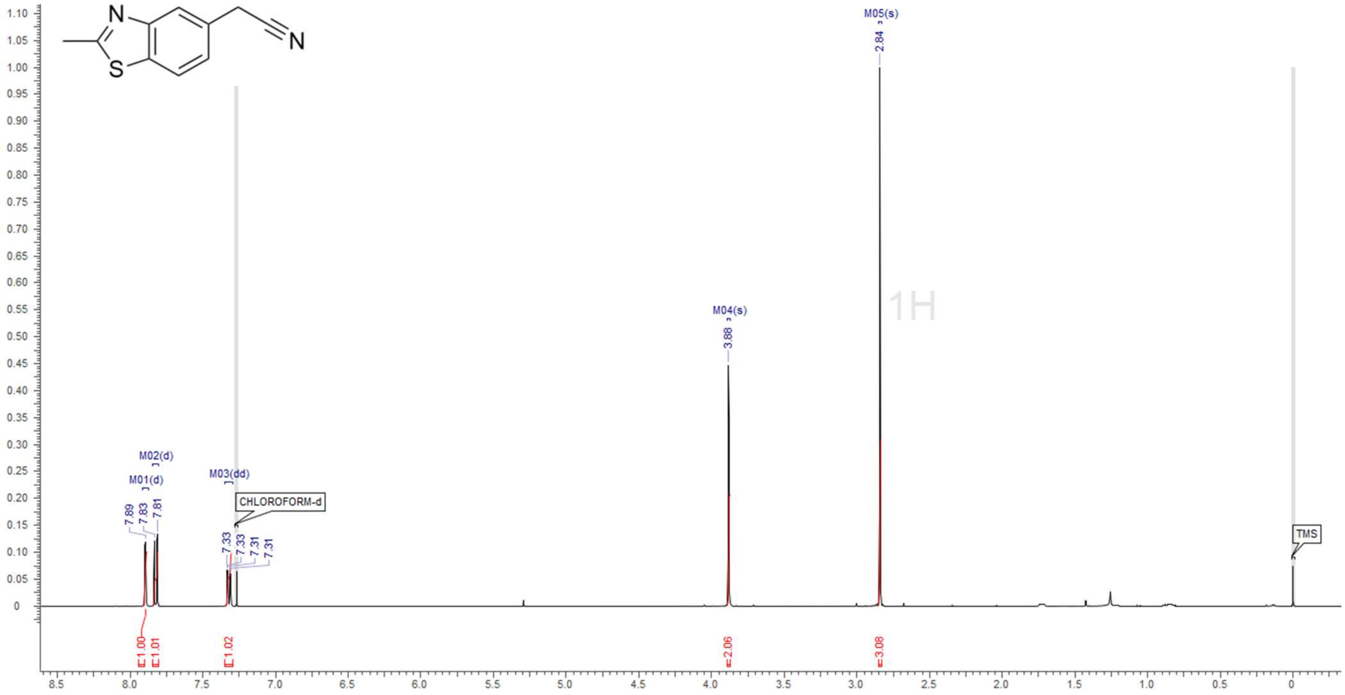

*Figure 38 ^{1}H NMR spectrum (400 MHz, $CDCl_3$) of compound **15**.*

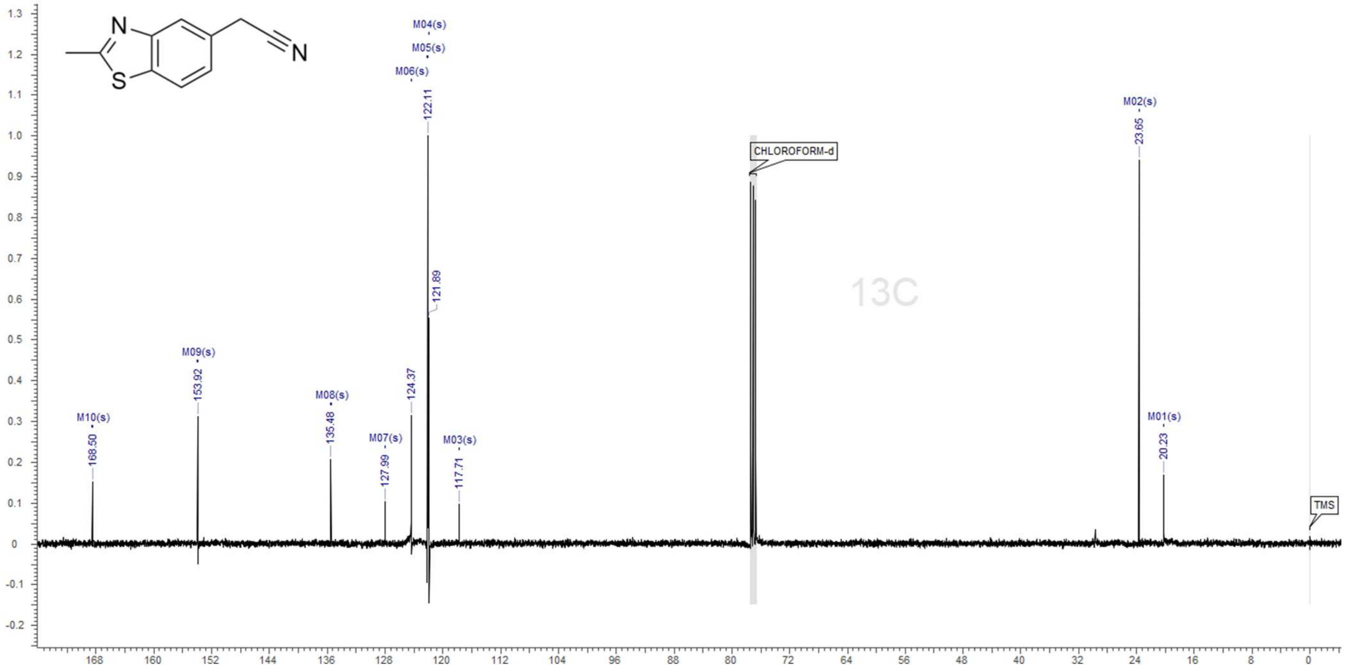

*Figure 39 ^{13}C NMR spectrum (101 MHz, $CDCl_3$) of compound **15**.*

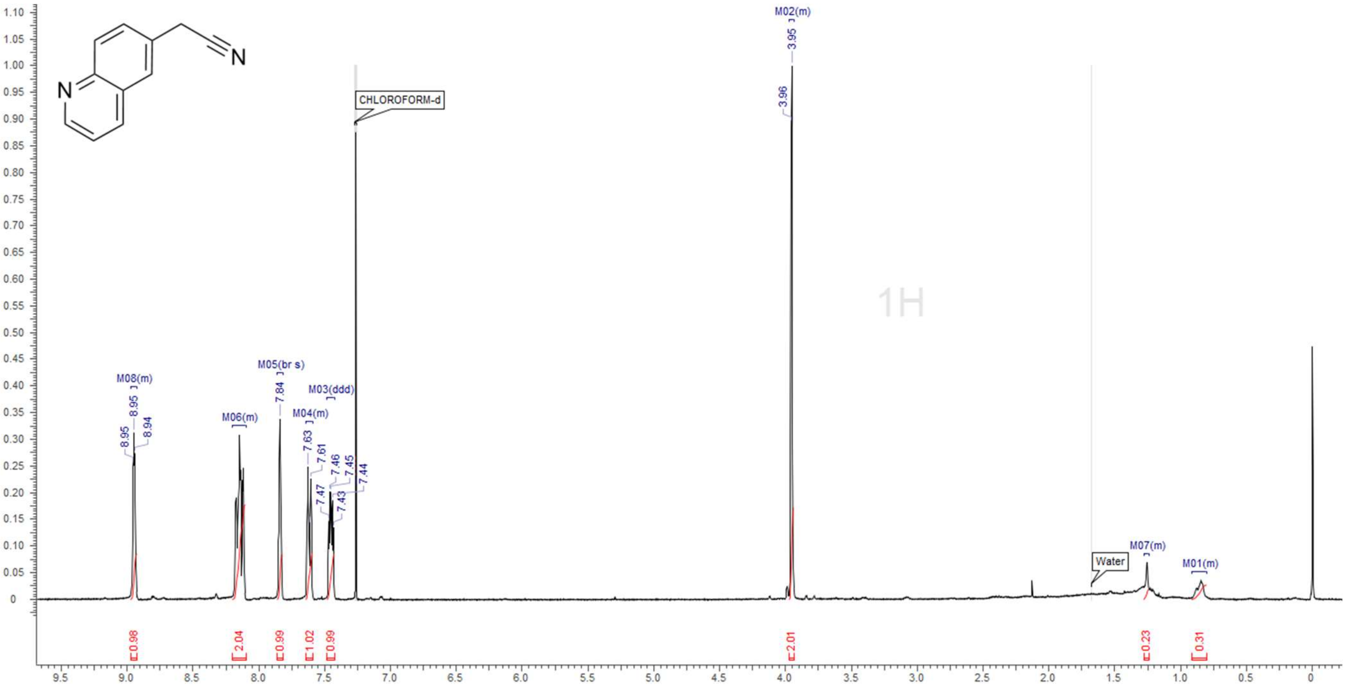

Figure 40 ¹H NMR spectrum (400 MHz, $CDCl_3$) of compound ***16****.*

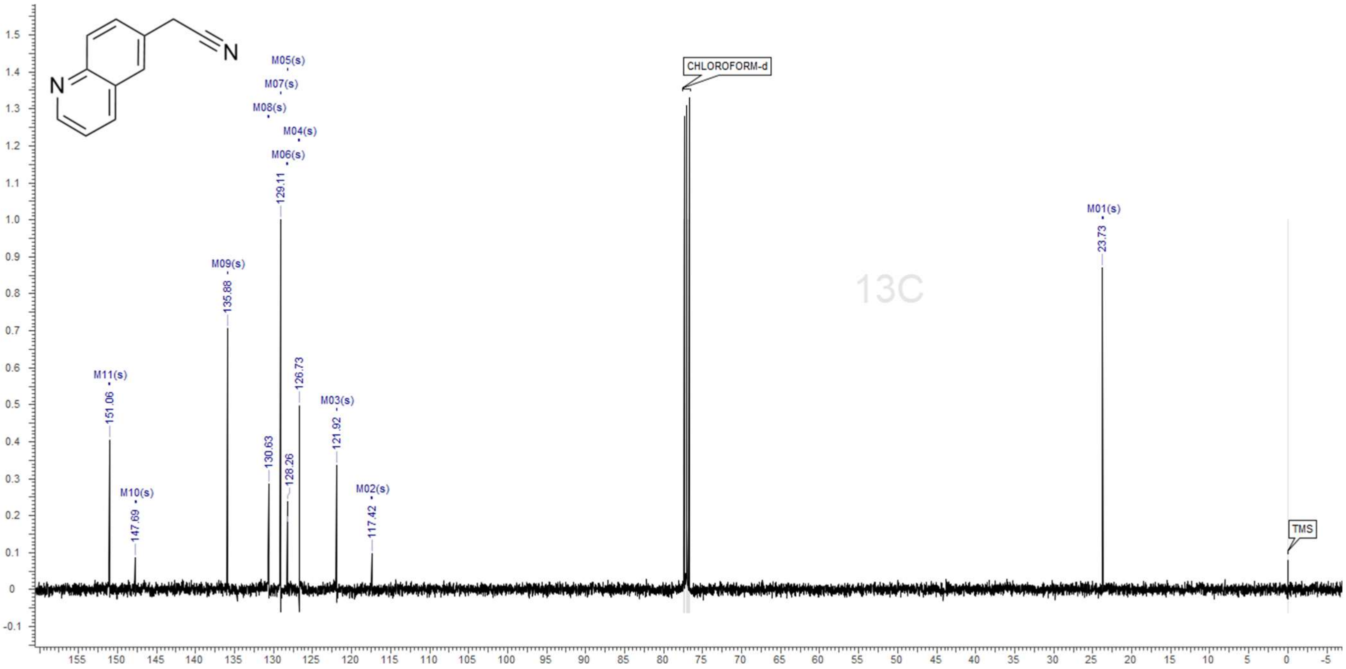

Figure 41 ¹³C NMR spectrum (101 MHz, $CDCl_3$) of compound ***16****.*

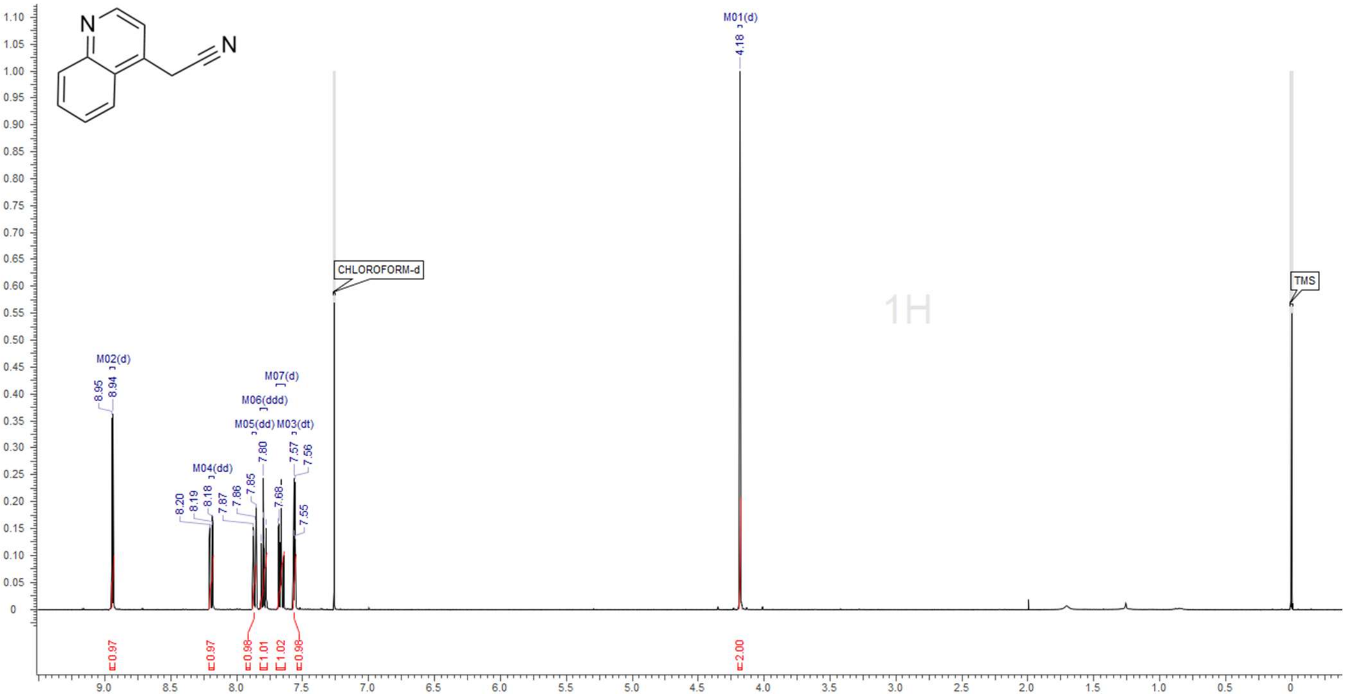

Figure 42 ^{1}H NMR spectrum (400 MHz, $CDCl_3$) of compound ***17****.*

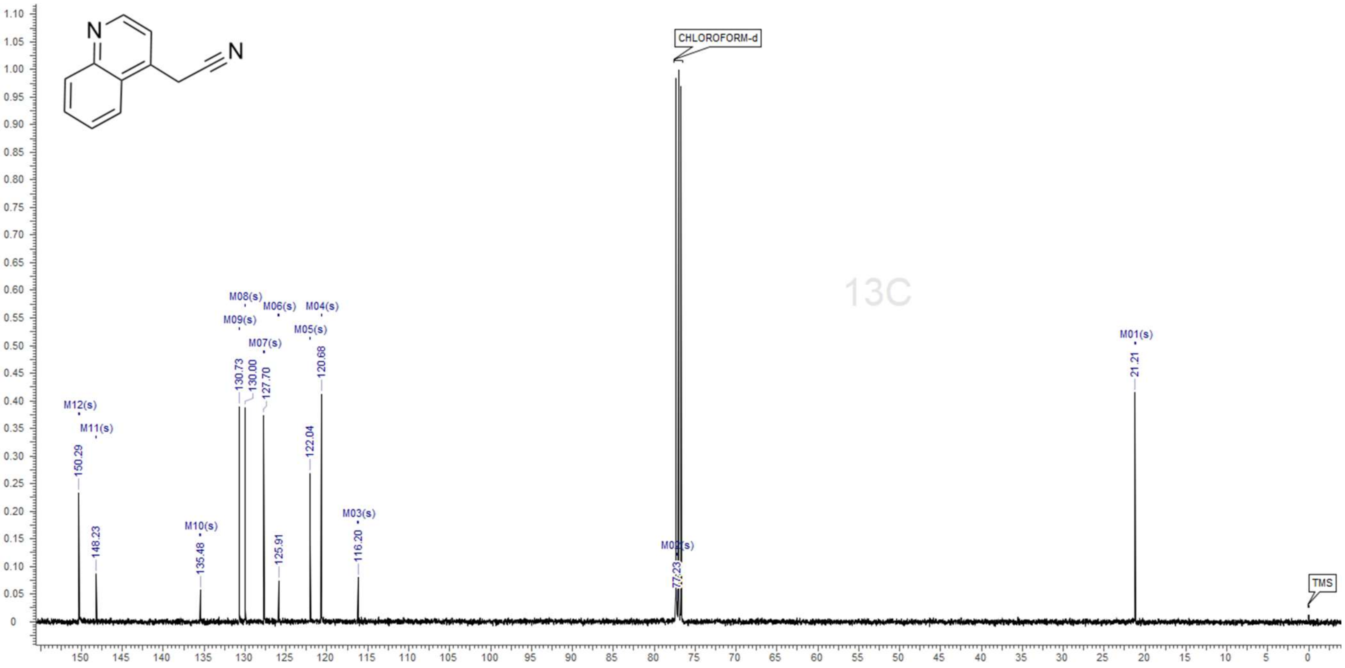

Figure 43 ^{13}C NMR spectrum (101 MHz, $CDCl_3$) of compound ***17****.*

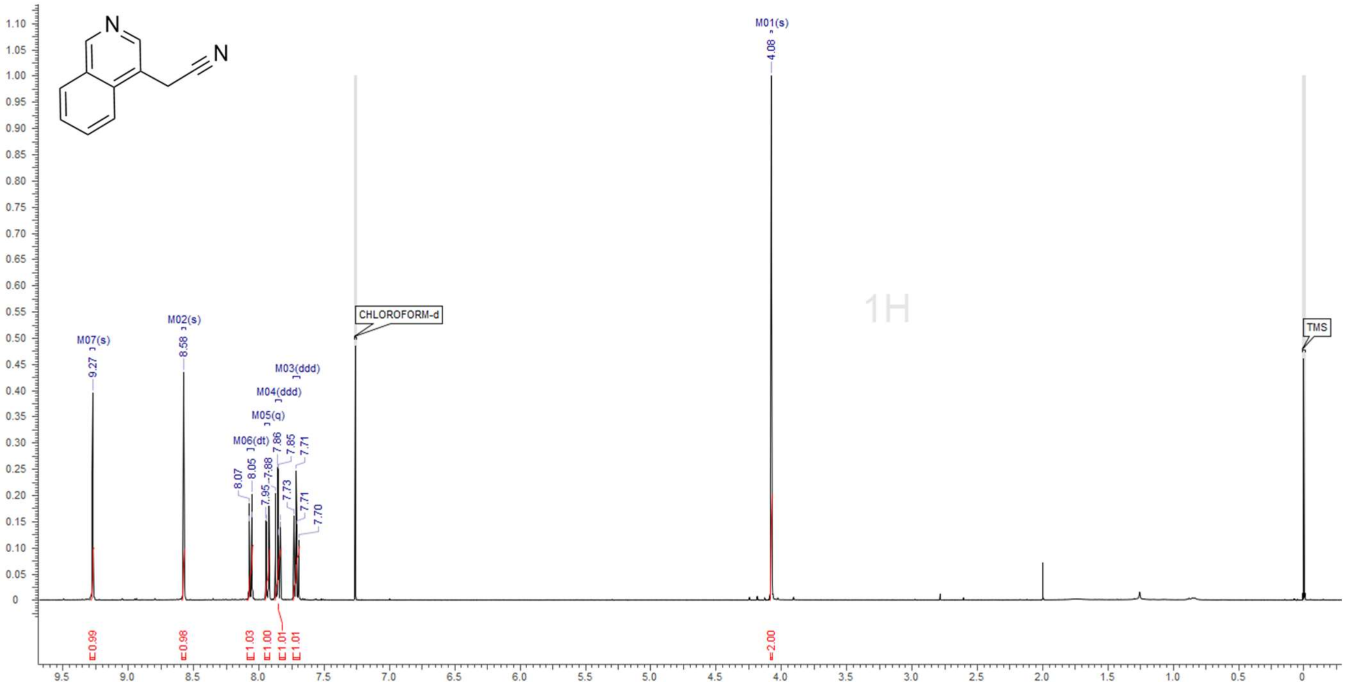

Figure 44 [1]*H NMR spectrum (400 MHz,* $CDCl_3$*) of compound* ***18****.*

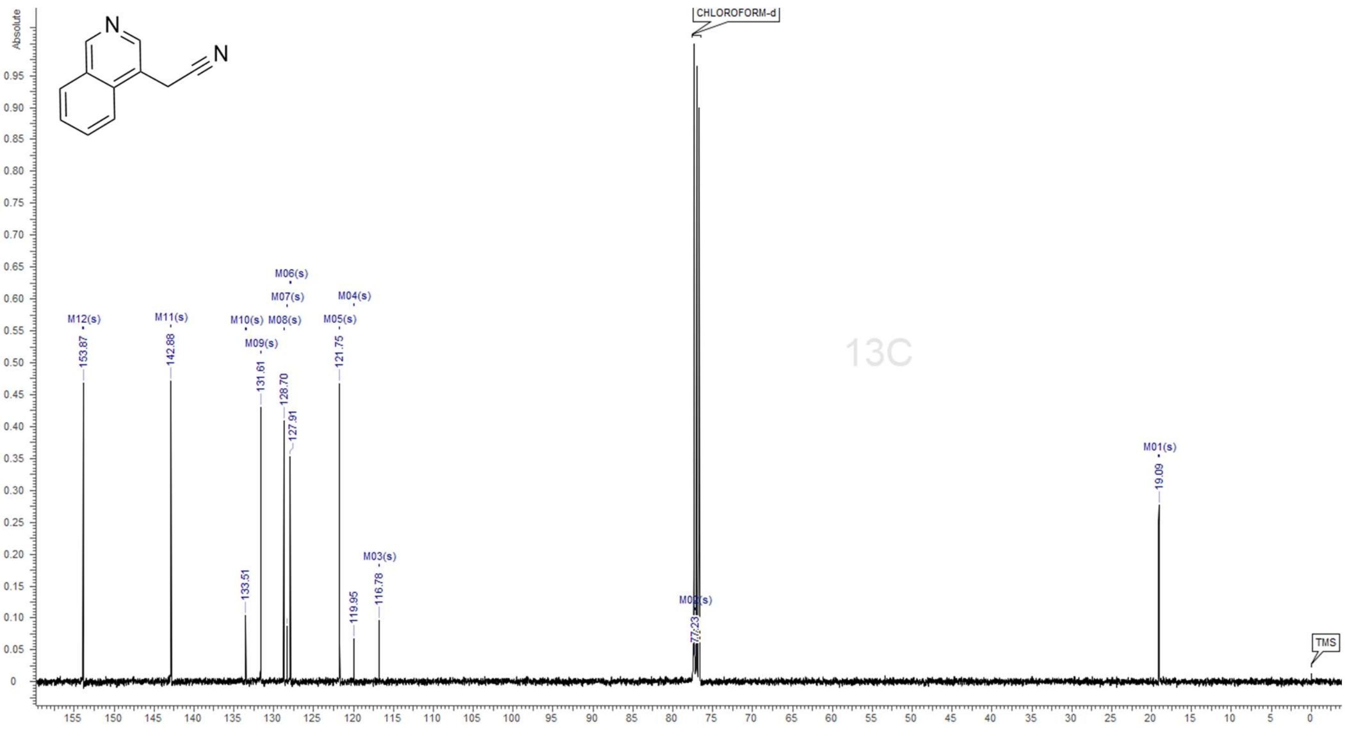

Figure 45 [13]*C NMR spectrum (101 MHz,* $CDCl_3$*) of compound* ***18****.*

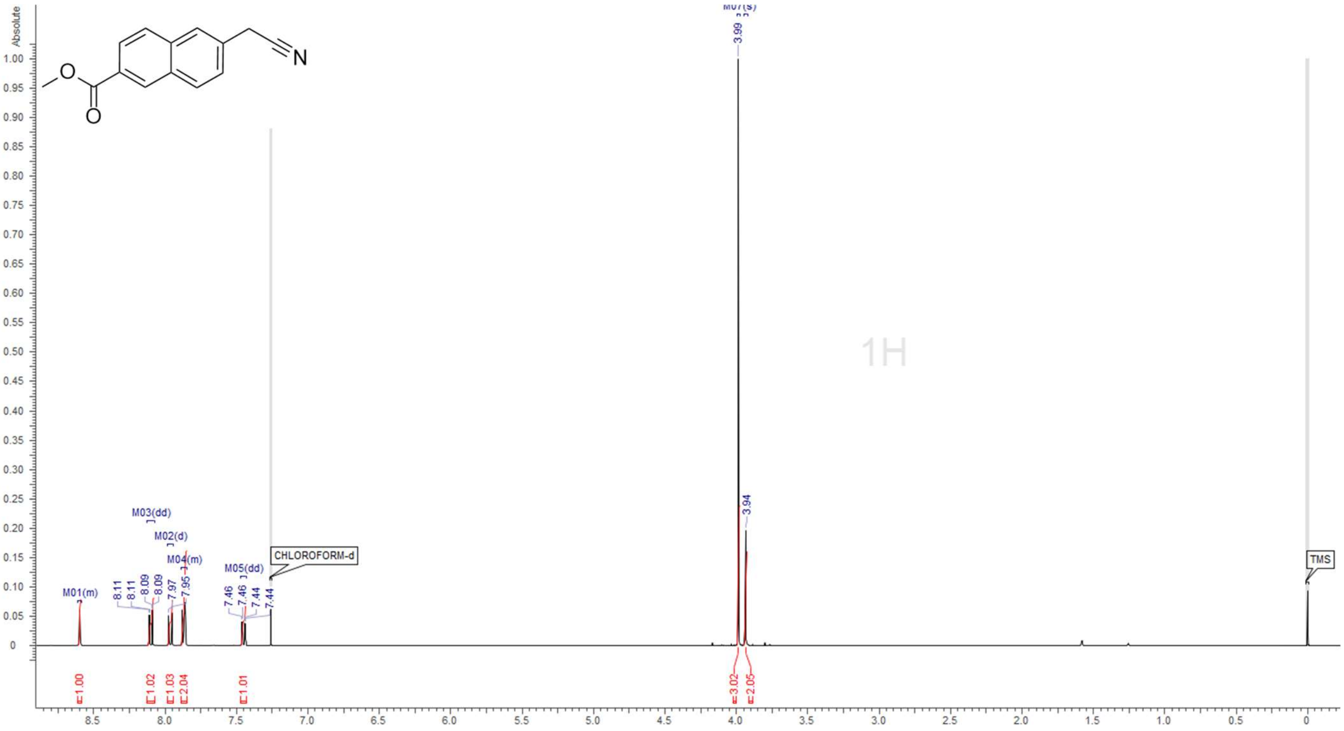

Figure 46 ^{1}H NMR spectrum (400 MHz, $CDCl_3$) of compound **19**.

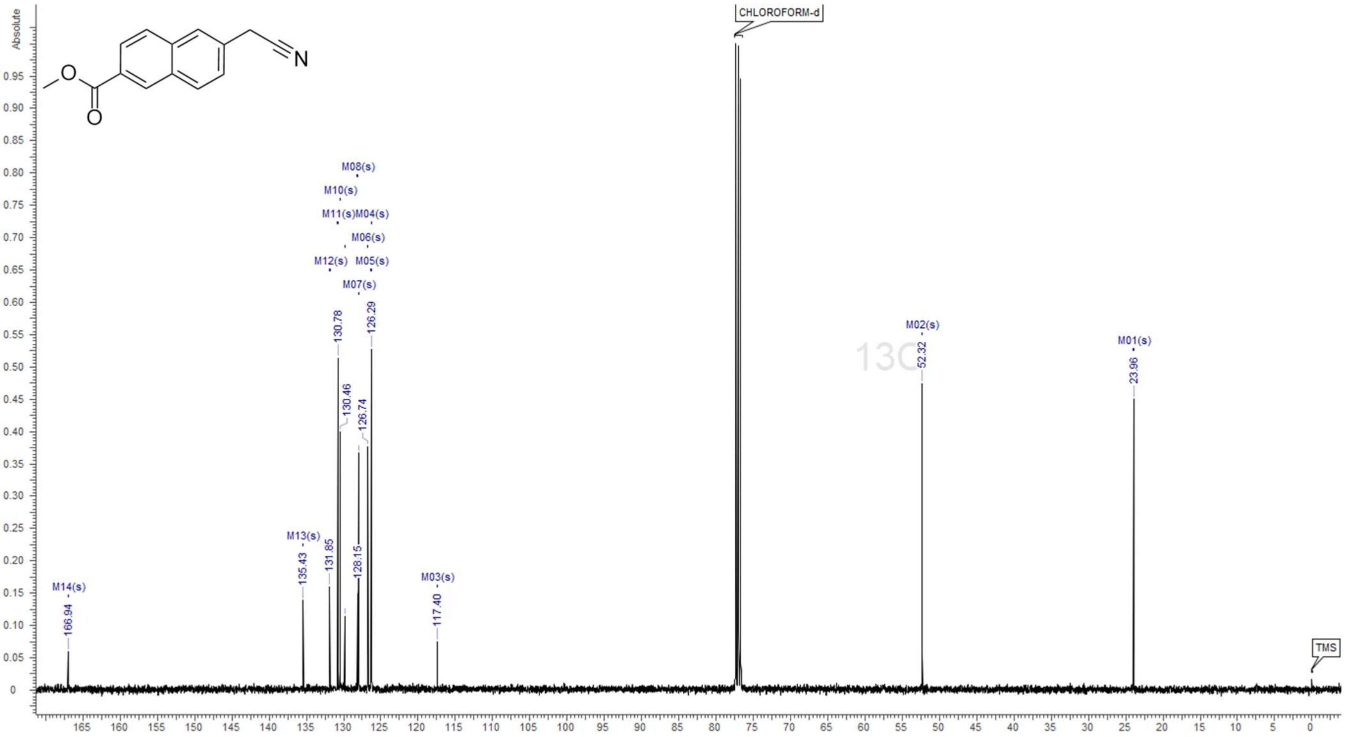

Figure 47 ^{13}C NMR spectrum (101 MHz, $CDCl_3$) of compound **19**.

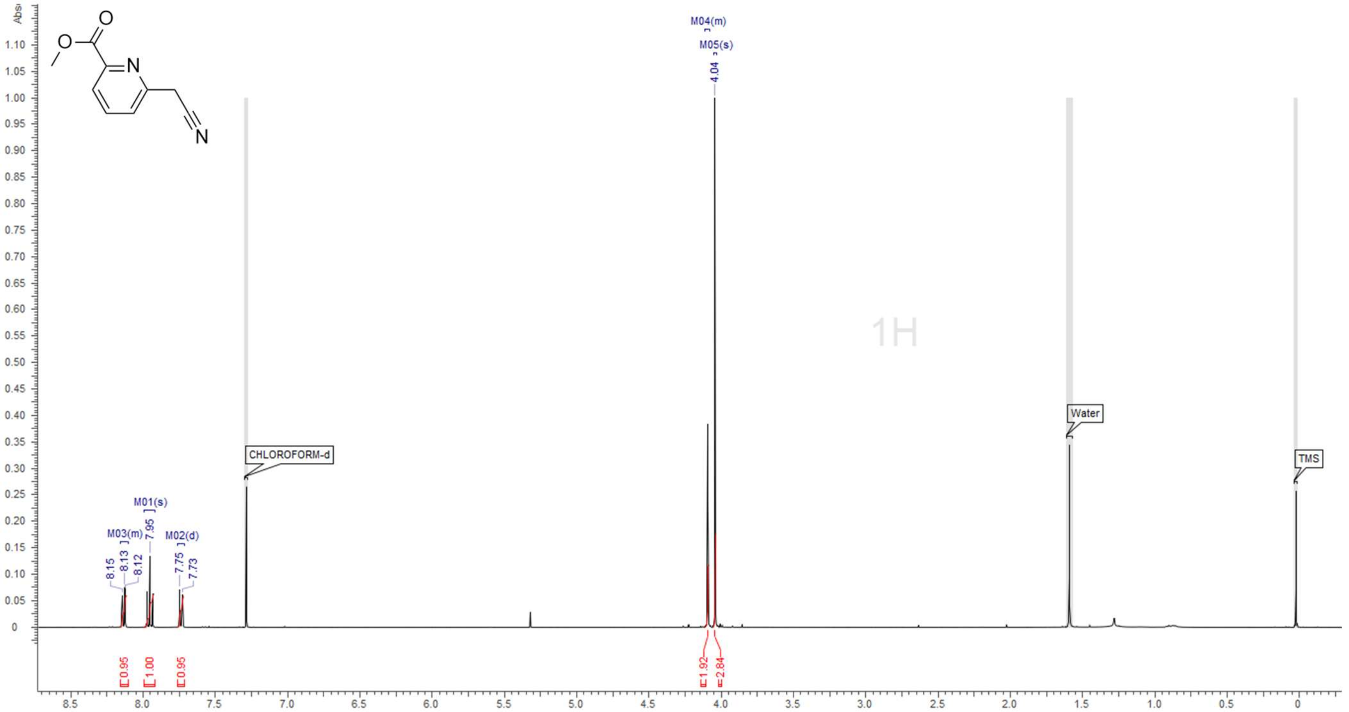

*Figure 48 ^{1}H NMR spectrum (400 MHz, $CDCl_3$) of compound **20**.*

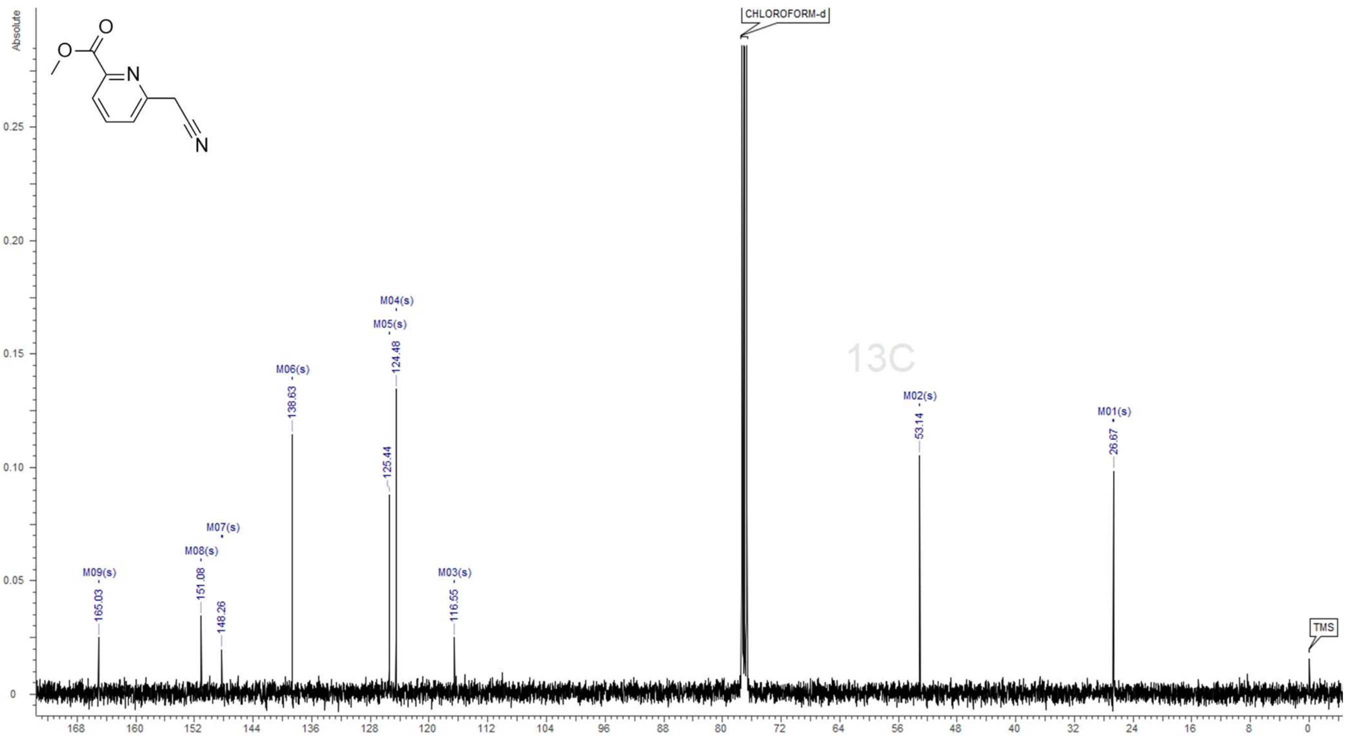

*Figure 49 ^{13}C NMR spectrum (101 MHz, $CDCl_3$) of compound **20**.*

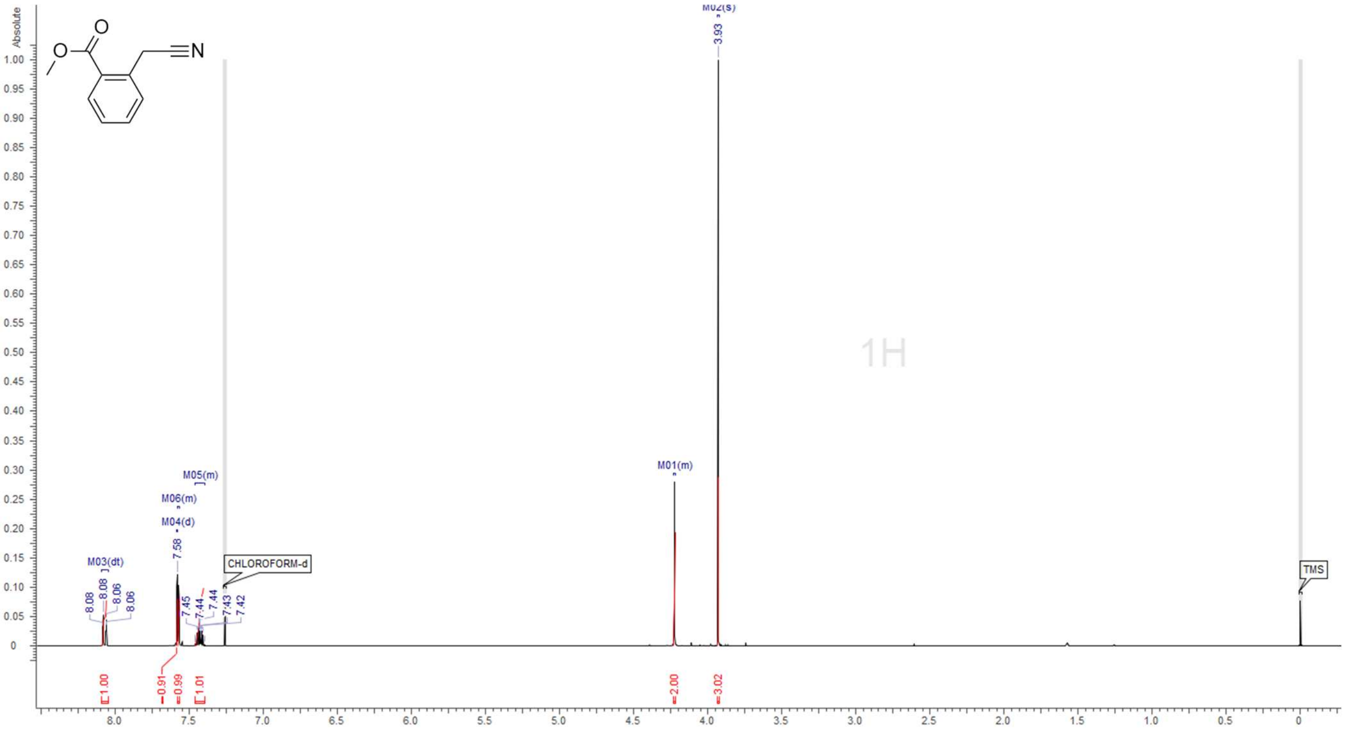

*Figure 50 ^{1}H NMR spectrum (400 MHz, $CDCl_3$) of compound **21**.*

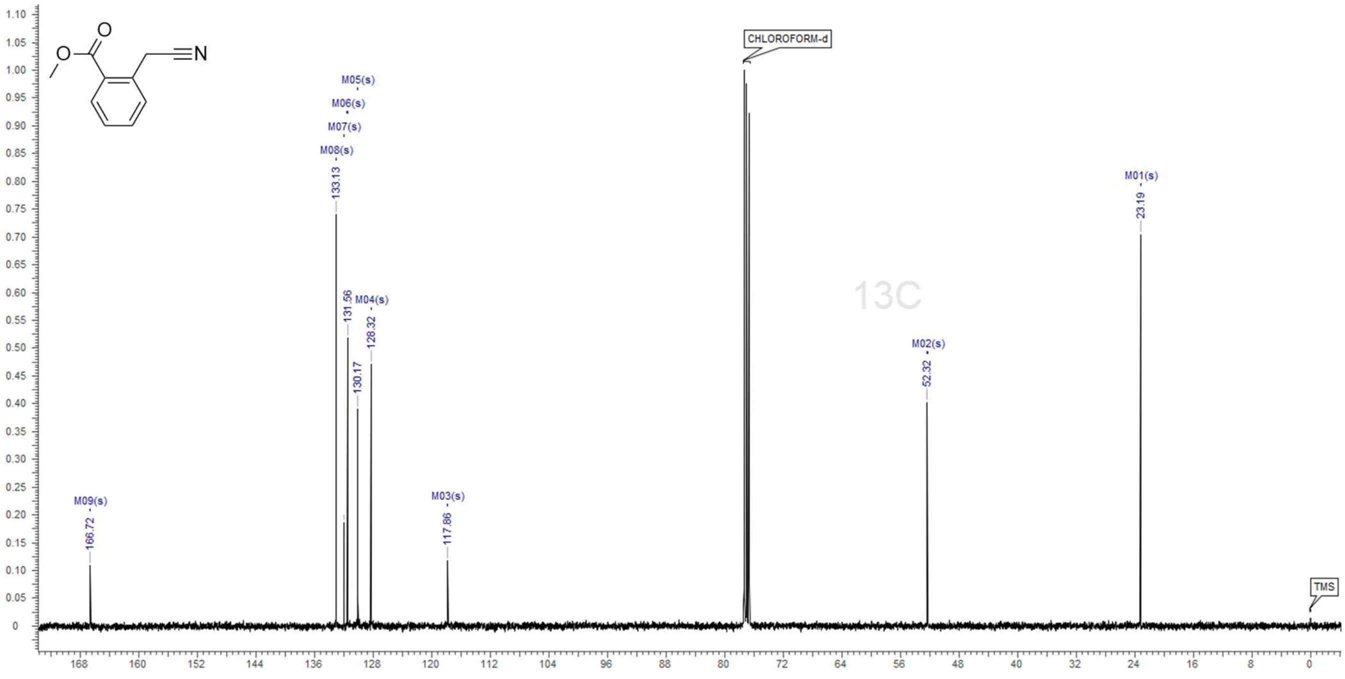

*Figure 51 ^{13}C NMR spectrum (101 MHz, $CDCl_3$) of compound **21**.*

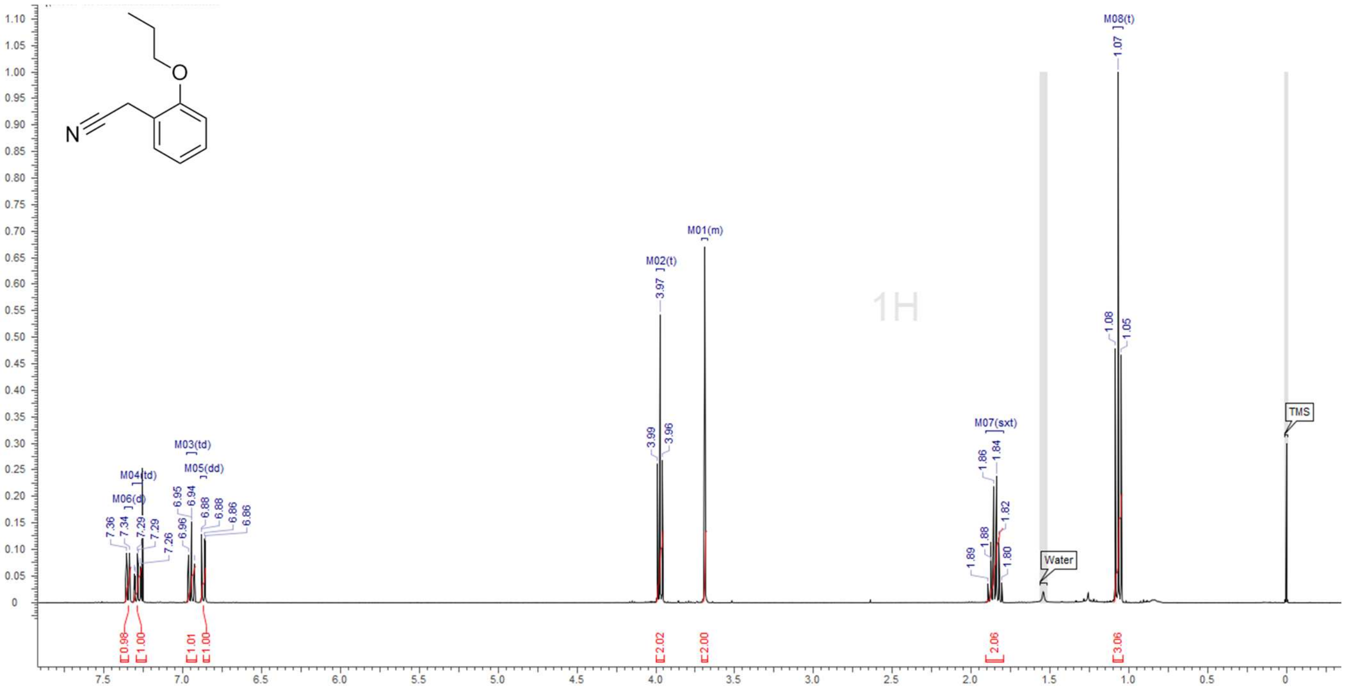

Figure 52 [1]*H NMR spectrum (400 MHz, CDCl*$_3$*) of compound* ***22***.

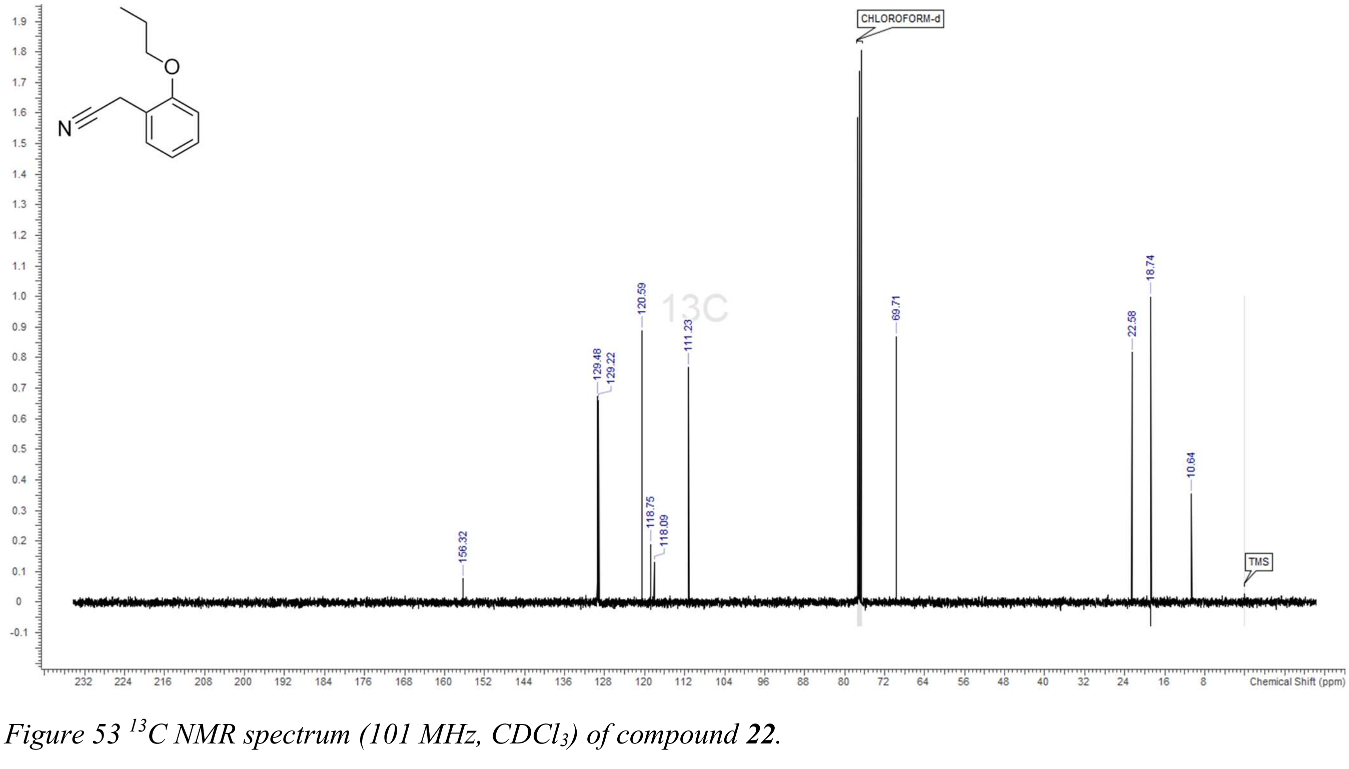

Figure 53 [13]*C NMR spectrum (101 MHz, CDCl*$_3$*) of compound* ***22***.

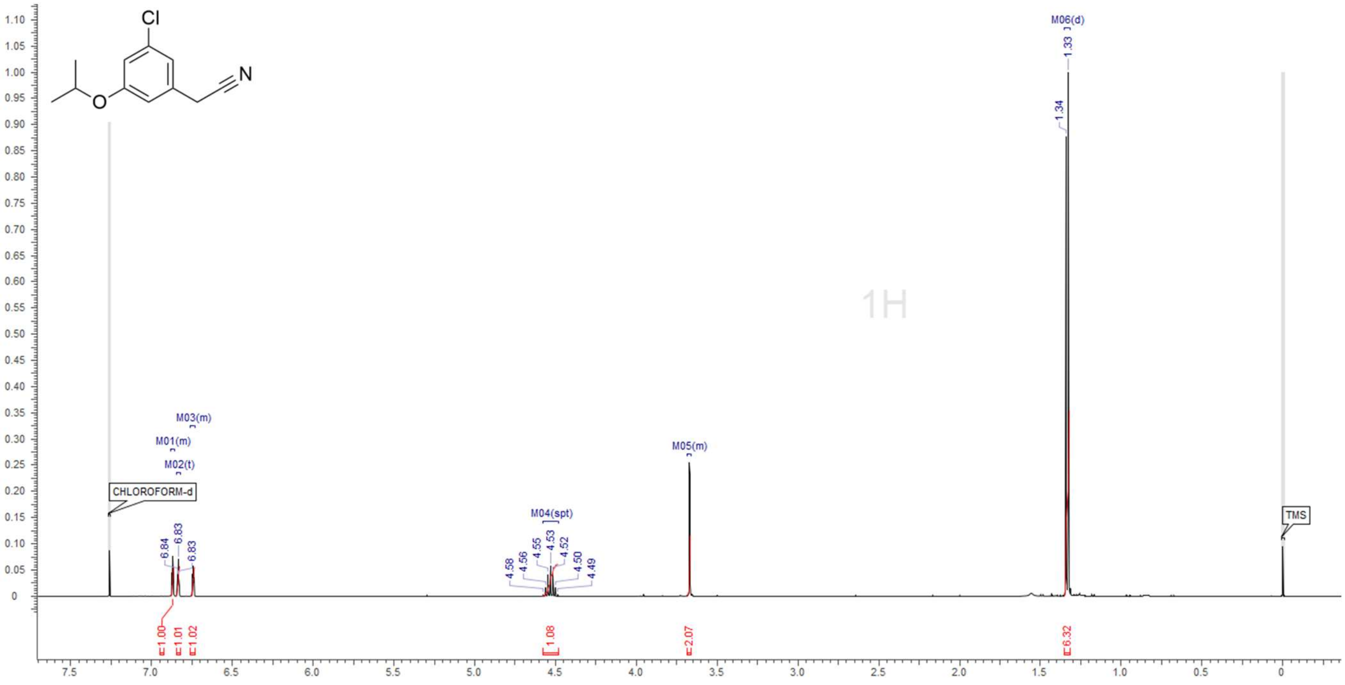

*Figure 54 ^{1}H NMR spectrum (400 MHz, $CDCl_3$) of compound **23**.*

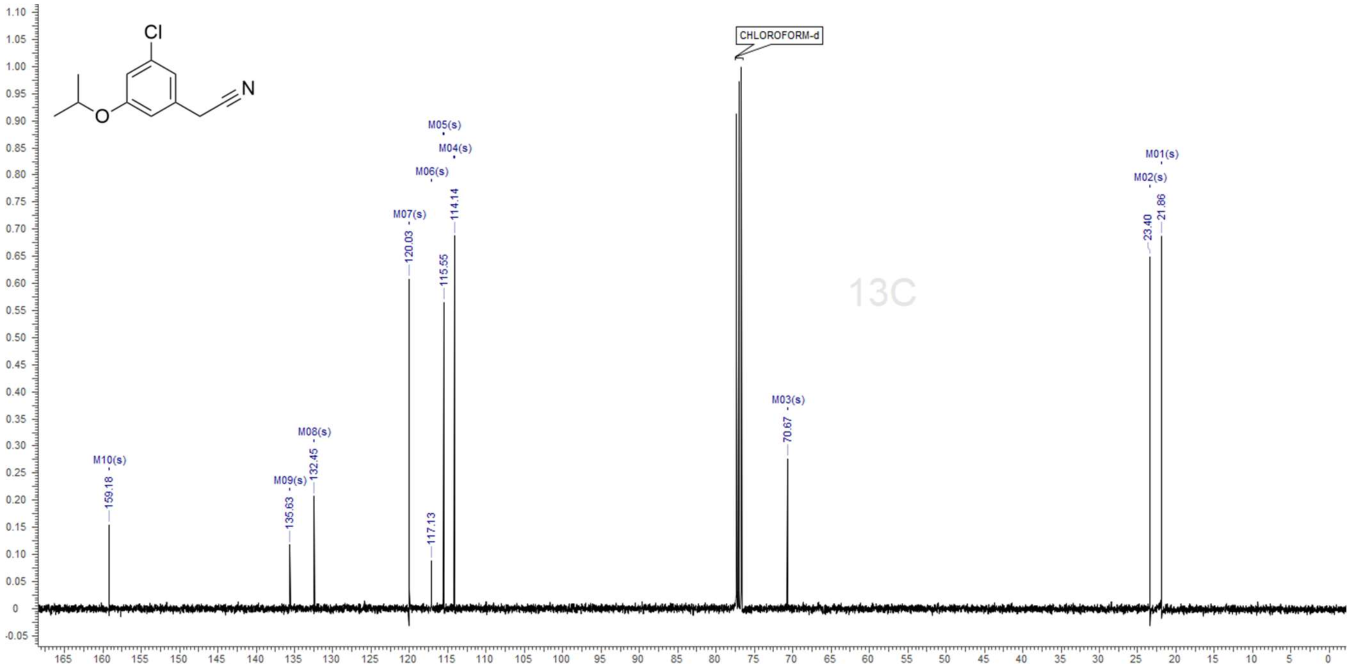

*Figure 55 ^{13}C NMR spectrum (101 MHz, $CDCl_3$) of compound **23**.*

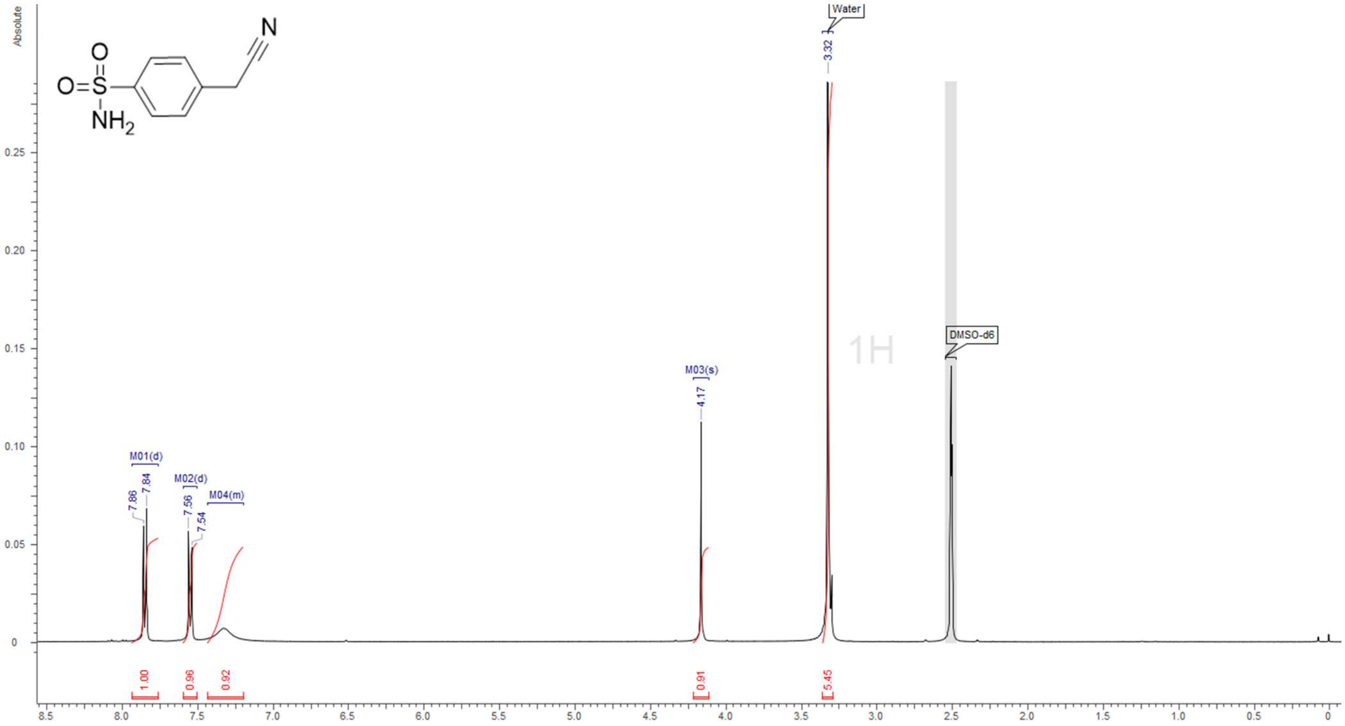

*Figure 56 ^{1}H NMR spectrum (400 MHz, DMSO-*d$_6$*) of compound* ***24****.*

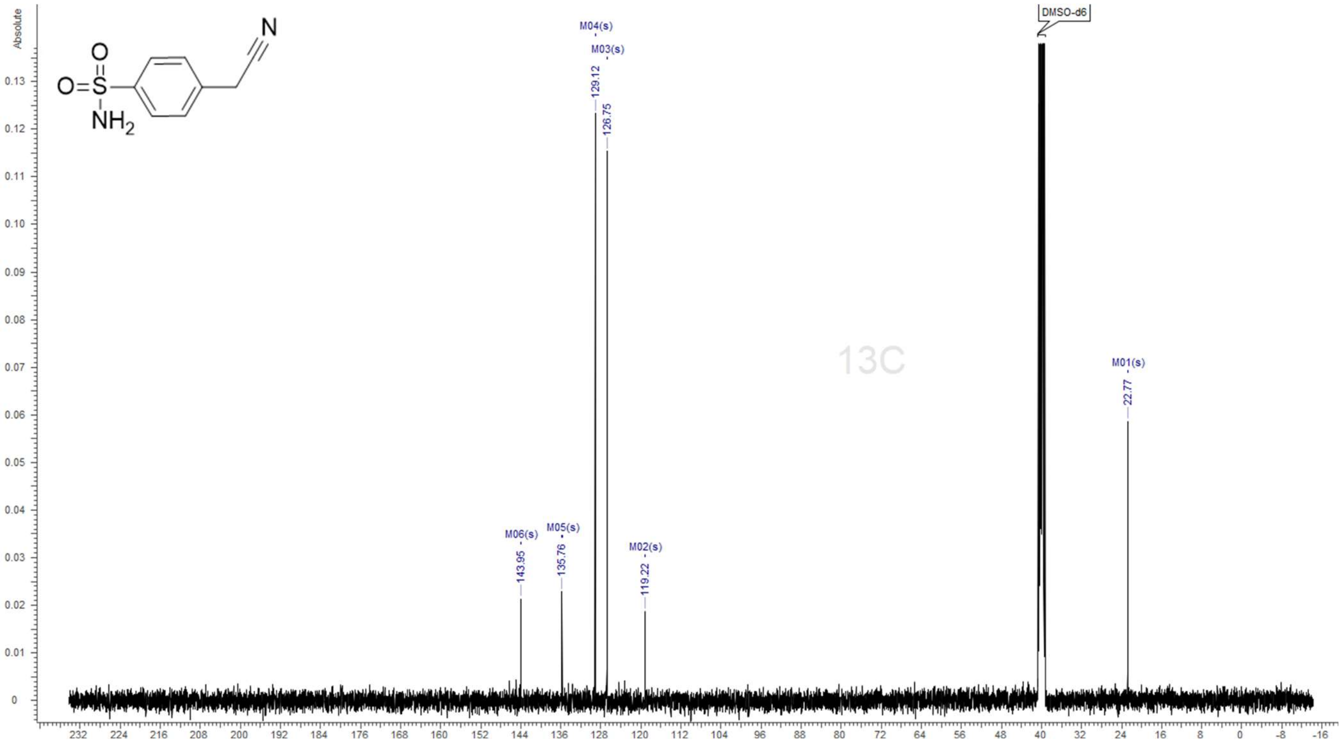

*Figure 57 ^{13}C NMR spectrum (101 MHz, DMSO-*d$_6$*) of compound* ***24****.*

7. References

[1] Katalyst D2D. “Advanced Chemistry Development, Inc.”, can be found under https://www.acdlabs.com/products/spectrus-platform/katalyst-d2d/ (accessed: 24/09/2024).
[2] a) N. A. Romero, D. A. Nicewicz, “Organic Photoredox Catalysis.” *Chem. Rev.* **2016**, *116*, 10075-10166.
b) E. Speckmeier, T. G. Fischer, K. A. Zeitler, “A Toolbox Approach To Construct Broadly Applicable Metal-Free Catalysts for Photoredox Chemistry: Deliberate Tuning of Redox Potentials and Importance of Halogens in Donor–Acceptor Cyanoarenes.” *J. Am. Chem. Soc.* **2018**, *140*, 15353-15365.
[3] C. K. Prier, D. A. Rankic, D. W. C. MacMillan, “Visible Light Photoredox Catalysis with Transition Metal Complexes: Applications in Organic Synthesis.” *Chem. Rev.* **2013**, *113*, 5322-5363.
[4] J.-H. Shon, T. S. Teets, “Photocatalysis with Transition Metal Based Photosensitizers.” *Comments Inorg. Chem* **2020**, *40*, 53-85.
[5] J. W. Beatty, C. R. J. Stephenson, “Amine Functionalization *via* Oxidative Photoredox Catalysis: Methodology Development and Complex Molecule Synthesis.” *Acc. Chem. Res.* **2015**, *48*, 1474-1484.
[6] E. Vinter, “LightMap.” SourceForge, can be found under https://sourceforge.net/projects/lightmap/ (accessed: 28/12/2023).
[7] G. F. Pauli, S.-N. Chen, C. Simmler, D. C. Lankin, T. Gödecke, B.U. Jaki, J. B. Friesen, J. B. McAlpine, J. G. Napolitano, “Correction to Importance of Purity Evaluation and the Potential of Quantitative ^{1}H NMR as a Purity Assay.” *J. Med. Chem.* **2015**, *58*, 9061-9061.
[8] Y. Chen, L. Xu, Y. Jiang, D. Ma, “Assembly of α-(Hetero)aryl Nitriles *via* Copper-Catalyzed Coupling Reactions with (Hetero)aryl Chlorides and Bromides.” *Angew. Chem. Int. Ed.* **2021**, *60*, 7082-7086.
[9] a) K. Kim, S. Lee, S. H. Hong, “Direct C(sp^3)–H Cyanation Enabled by a Highly Active Decatungstate Photocatalyst.” *Org. Lett.* **2021**, *23*, 5501-5505.
b) L. Yang, Z. Huang, G. Li, W. Zhang, R. Cao, C. Wang, J. Xiao, D. Xue, “Synthesis of Phenols: Organophotoredox/Nickel Dual Catalytic Hydroxylation of Aryl Halides with Water.” *Angew. Chem., Int. Ed.* **2018**, *57*, 1968-1972.
[10] X. Meng, D. Chen, R. Liu, P. Jiang, S. Huang, “Synthesis of 2-(Cyanomethyl)benzoic Esters *via* Carbon–Carbon Bond Cleavage of Indanones.” *J. Org. Chem.* **2021**, *86*, 10852-10860.
[11] A. Rovira, M. Pujals, A. Gandioso, M. López-Corrales, M. Bosch, V. Marchán, “Modulating Photostability and Mitochondria Selectivity in Far-Red/NIR Emitting Coumarin Fluorophores through Replacement of Pyridinium by Pyrimidinium.” *J. Org. Chem.* **2020**, *85*, 6086-6097.
[12] Y. Huang, Y. Yu, Z. Zhu, C. Zhu, J. Cen, X. Li, W. Wu, H. Jiang, “Copper-Catalyzed Cyanation of N-Tosylhydrazones with Thiocyanate Salt as the “CN” Source.” *J. Org. Chem.* **2017**, *82*, 7621-7627.
[13] D. S. Novikova, F. Darwish, T. A. Grigoreva, V. G. Tribulovich, “Development of a Reproducible and Scalable Method for the Synthesis of Biologically Active Pyrazolo[1,5-a]pyrimidine Derivatives.” *Russ. J. Gen. Chem.* **2023**, *93*, 1040-1049.
[14] H. D. Pickford, J. Nugent, B. Owen, J. J. Mousseau, R. C. Smith, E. A. Anderson, “Twofold Radical-Based Synthesis of N,C-Difunctionalized Bicyclo[1.1.1]pentanes.” *J. Am. Chem. Soc.* **2021**, *143*, 9729-9736.
[15] a) R. J. Wiles, J. P. Phelan, G. A. Molander, “Metal-free defluorinative arylation of trifluoromethyl alkenes *via* photoredox catalysis.” *Chem. Comm.* **2019**, *55*, 7599-7602.
b) G. H. Lovett, S. Chen, X.-S. Xue, K. N. Houk, D. W. C. MacMillan, “Open-Shell Fluorination of Alkyl Bromides: Unexpected Selectivity in a Silyl Radical-Mediated Chain Process.” *J. Am. Chem. Soc.* **2019**, *141*, 20031-20036.
c) P. Bellotti, H.-M. Huang, T. Faber, R. Laskar, F. Glorius, “Catalytic defluorinative ketyl–olefin coupling by halogen-atom transfer.” *Chem. Sci.* **2022**, *13*, 7855-7862.

[16] T. U. Connell, C. L. Fraser, M. L. Czyz, Z. M. Smith, D. J. Hayne, E. H. Doeven, J. Agugiaro, D. J. D. Wilson, J. L. Adcock, A. D. Scully, D. E. Gómez, N. W. Barnett, A. Polyzos, P. S. Francis, “The Tandem Photoredox Catalysis Mechanism of $[Ir(ppy)_2(dtbpy)]^+$ Enabling Access to Energy Demanding Organic Substrates.” *J. Am. Chem. Soc.* **2019**, *141*, 17646-17658.
[17] P. Zhang, C. C. Le, D. W. C. MacMillan, “Silyl Radical Activation of Alkyl Halides in Metallaphotoredox Catalysis: A Unique Pathway for Cross-Electrophile Coupling.” *J. Am. Chem. Soc.* **2016**, *138*, 8084-8087.
[18] D. DiRocco, “Merck photocatalysis chart.” can be found under https://macmillan1.wpengine.com/wp-content/uploads/Merck-Photocatalysis-Chart.pdf, **2014** (accessed: 26/08/2024).
[19] M. Kudisch, R. X. Hooper, L. K. Valloli, J. D. Earley, A. Zieleniewska, J. Yu, S. DiLuzio, R. W. Smaha, H. Sayre, X. Zhang, M. J. Bird, A. A. Cordones, G. Rumbles, O. G. Reid, “Photolytic activation of $Ni^{(II)}X_2L$ explains how Ni-mediated cross coupling begins.” *Nat. Commun.* **2025**, *16*, 5530.
[20] S. DiLuzio, L. K. Valloli, M. Kudisch, D. T. Chambers, G. Rumbles, O.G. Reid, M. J. Bird, H. J. Sayre, “Reconceptualizing the Ir^{III} Role in Metallaphotoredox Catalysis: From Strong Photooxidant to Potent Energy Donor.” *ACS Catal*. **2024**, *14*, 11378-11388.
